# Asymmetric 1,4-Michael Addition Reaction of Azadienes with α-Thiocyanoindanones Catalyzed by Bifunctional Chiral Squaramide

**DOI:** 10.3390/molecules26175146

**Published:** 2021-08-25

**Authors:** Xiao-Yan Dong, Da-Ming Du

**Affiliations:** School of Chemistry and Chemical Engineering, Beijing Institute of Technology, Beijing 100081, China; 18332766507@163.com

**Keywords:** asymmetric catalysis, organocatalysis, 1,4-Michael addition, azadiene, benzofuran

## Abstract

In this paper, the organocatalytic asymmetric 1,4-Michael addition reaction of azadienes and α-thiocyanoindanones was investigated. A series of chiral benzofuran compounds containing thiocyano group and quaternary carbon center were synthesized in moderate yields with good enantioselectivities (up to 90:10 *er*) and high diastereoselectivities (up to >95:5 *dr*). This is the first case of 1,4-Michael addition reaction using α-thiocyanoindanones to obtain a series of chiral thiocyano compounds and further broaden the scope of application of azadiene substrates. In addition, a possible reaction mechanism is also described in the article.

## 1. Introduction

**Figure 1:**
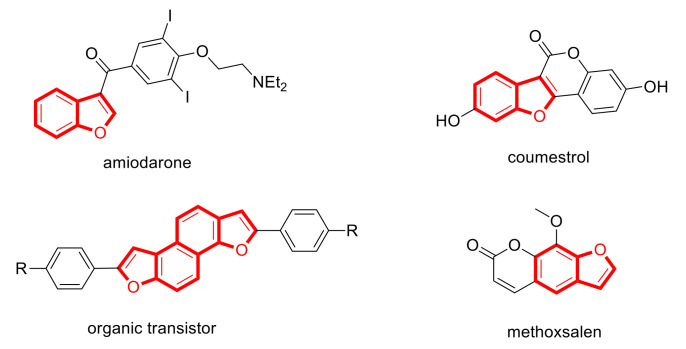
Selected biologically active benzofuran derivatives.

Benzofuran derivatives are an important class of heterocyclic compounds in many biologically active natural products [1,2,3,4]. Compounds containing benzofuran structural motifs exhibit excellent anticancer, antioxidation, and antifungal activities, and they can also be used as important structural motifs for some organic materials and drug molecules (Figure 1) [5,6,7,8]. The development of efficient and convenient ways to obtain compounds containing benzofuran structural motifs is the common goal pursued by many organic chemists. As far as we know, azadienes derived from benzofurans can undergo 1,4-Michael addition reactions with suitable nucleophiles due to their high reactivity to quickly construct multisubstituted benzofuran derivatives, and a variety of benzofuran derivatives have been successfully obtained by using azadienes [9,10,11,12,13,14].

**Figure 2:**
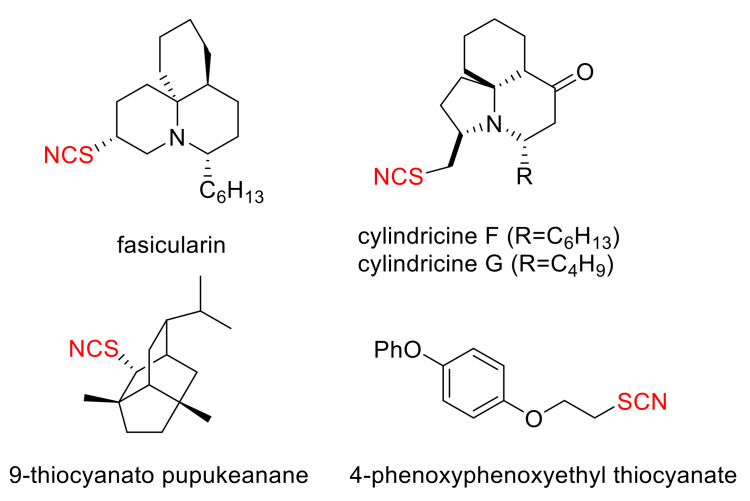
Selected biologically active molecules containing thiocyano group.

**Scheme 1:**
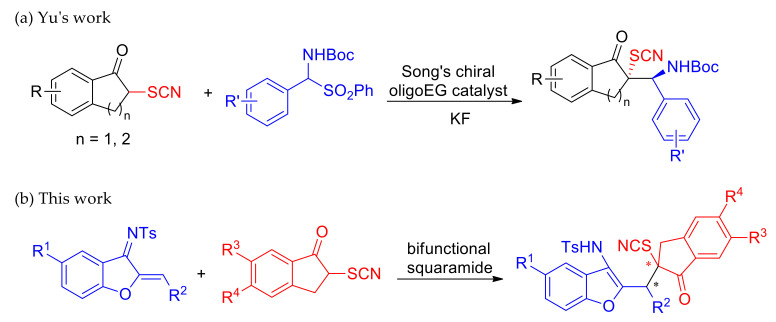
The asymmetric Mannich reaction of α-thiocyanoindanones and this work.

In addition, thiocyanate compounds have various biological activities such as being anticancer, antimicrobial, and insecticidal; they have a wide range of applications in the fields of medicine, pesticides, and materials (Figure 2). Thiocyanate compounds are also important synthetic intermediates for obtaining sulfur-containing compounds [15,16,17,18,19,20]. Therefore, thiocyanate compounds have attracted the attention of more and more organic chemists. As far as we know, thiocyanate or trimethylsilyl alkyl isothiocyanates can react with substrates with leaving groups to obtain thiocyano compounds. Mainly include ^−^SCN nucleophilic substitution reaction, ^+^SCN electrophilic substitution reaction, and •SCN radical reaction. In addition, the reaction of cyanating reagents with sulfur-containing substrates is also an important method for obtaining thiocyanate compounds. Mainly include ^−^CN nucleophilic substitution reaction, ^+^CN electrophilic substitution reaction, and •CN radical reaction [21,22,23,24,25,26]. In recent years, the application research of thiocyanate compounds has also developed rapidly. However, in the development process, it also faces problems such as a low utilization rate of substrate atoms and poor economy [27,28]. α-Thiocyanoindanone is a nucleophile with a thiocyanato functional group; notably, by using α-thiocyanoindanone as a nucleophile, thiocyanate compounds can be obtained efficiently and conveniently. However, at present, the development of α-thiocyanoindanone in the asymmetric field is still relatively limited. To the best of our knowledge, there are few reports on the asymmetric catalytic reaction of α-thiocyanoindanones. In 2017, Yu and coworkers used α-thiocyanoindanones and α-aminosulfones to undergo a Mannich reaction, which obtained a series of optically active 2-thiocyanato-2-(1-aminoalkyl)-substituted 1-tetralones and 1-indanones (Scheme 1a) [29]. Regrettably, there has been no report about a series of optically active thiocyanate compounds by using α-thiocyanoindanone as a Michael donor to undergo a 1,4-addition asymmetric reaction.

Therefore, in this paper, we envisioned a 1,4-Michael addition reaction between azadienes and α-thiocyanoindanones, which could afford a series of optically active compounds containing benzofuran motif and thiocyano group with excellent diastereoselectivities and good enantioselectivities. This reaction not only provides a new strategy for the synthesis of thiocyanate compounds, but also the types of nucleophiles which react with azadienes are further expanded (Scheme 1b).

## 2. Results and Discussion

**Figure 3:**
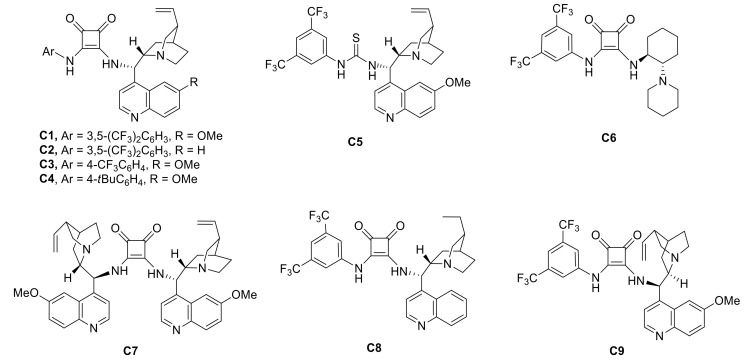
The screened organocatalysts.

In the beginning, a series of organic catalysts were used to select the optimal catalyst for the 1,4-Michael addition reaction between azadienes and α-thiocyanoindanones. We first used a quinine-derived squaramide **C1** to catalyze 1,4-Michael addition reaction of azadiene **1a** and α-thiocyanoindanone **2a** in dichloromethane solution. Fortunately, the target product **3aa** was obtained with moderate yield and enantioselectivity (Table 1, entry 1). Encouraged by this result, several other catalysts **C2**–**C9** (Figure 3) were screened to catalyze the 1,4-Michael addition reaction (Table 1, entries 2–9). However, no results better than **C1** were obtained. Therefore, we still use **C1** as the optimal catalyst.

Next, the influence of the solvent effect on the reaction was examined. A total of 14 solvents (Table 1, entries 10–23) was evaluated. Surprisingly, when ethyl acetate was used, although the product yield was lower than that using dichloromethane, the enantioselectivity and stereoselectivity were better than using dichloromethane. Therefore, ethyl acetate was chosen as the best solvent. After the optimal solvent was determined, we believed that temperature and catalyst loading may also have influence on the reaction outcome. As expected, when the catalyst loading was reduced to 2.5 mol%, although the enantioselectivity was increased to 86:14 *er*, the diastereoselectivity decreased to 89:11 *dr* (Table 1, entry 24). Considering that reducing the catalyst loading can increase the enantioselectivity, we decided to further reduce the catalyst loading to 1 mol%. Regrettably, both the *er* and *dr* values decreased (Table 1, entry 25). When the catalyst loading was increased to 10 mol%, the enantioselectivity increased to 87:13 *er*, and the diastereoselectivity decreased to 71:29 *dr*. (Table 1, entry 26); therefore, we decided to still use 5 mol% catalyst loading. We also tried to lower the temperature to 0, −10, and −20 °C (Table 1, entries 27–29). Fortunately, when the temperature was lowered to −10 °C, the *er* value could be increased to 90:10 *er*. In addition, we reduced the concentration of the reaction solution and put azadiene and α-thiocyanoindanone in 2 mL ethyl acetate. Compared with entry 28, the results showed that the yield, enantioselectivity, and diastereoselectivity decreased (Table 1, entry 30). Therefore, the best result was obtained using 0.05 mmol **1a**, 0.04 mmol **2a**, and 5 mol% **C1** in EtOAc at −10 °C.

After the optimal reaction conditions were determined, the influence of different substituents on the substrates on the effect of this reaction was investigated. Firstly, the influence of different substituents in the azadienes **1** on the reaction was investigated, and the results are listed in Table 2 (entries 1–13). Regardless of whether R^2^ was an aromatic ring substituted with para-electron-withdrawing group or electron-donating group (Table 2, entries 2–7), the reaction of azadienes **1** with α-thiocyanoindanone **2a** could basically maintain medium yields (58% to 87%), moderate to excellent diastereoselectivity (75:25 *dr* to >95:5 *dr*), and good enantioselectivities (79:21 *er* to 90:10 *er*).

When we used azadienes (R^2^ is a *meta*-substituted aromatic ring) for the reaction (Table 2, entries 8 and 9), the enantioselectivity, yield, and diastereoselectivity of the electron-withdrawing group decreased. By contrast, the electron-donating group could maintain the enantioselectivity, yield, and diastereoselectivity. It is considered that the *meta*-bromo substitution makes the solubility of azadiene in ethyl acetate worse, which leads to a worsening of the chiral control effect. We also investigated the influence of the azadiene (R^2^ is *ortho*-substituted aryl) on the reaction, which was affected by steric hindrance; it caused the reaction product to be only a trace amount. In addition, azadienes bearing 2-naphthyl, 2-thienyl, and 2-pyridinyl groups (Table 2, entries 10–12) also participated in the addition reaction to obtain corresponding products in good yields and *er* value. Finally, we also investigated azadiene when the R^1^ is methyl (Table 2, entries 13). Unfortunately, the enantioselectivity of this substrate has also decreased accordingly. Therefore, we found that the azadienes substituted by aryl groups at different positions have different control effects on the reaction. Generally speaking, the reaction has good universality for azadiene substrates.

In the next part, the effect of different substitutions of α-thiocyanoindanone on the reaction was further investigated. We mainly examined the 6-electron withdrawing/donating group and 5-electron withdrawing/donating group-substituted α-thiocyanoindanones. As shown in Table 3, entries 2–9, all the tried 5- and 6-substituted α-thiocyanoindanones can undergo 1,4-Michael addition with azadiene **1a** to give the target products in moderate yields (43% to 66%). Among them, the electron-donating group substitution of α-thiocyanoindanone is significantly better than the electron-withdrawing group substitution. The 6-position except -OMe-substituted α-thiocyanindanone reacts with azadiene **1a** to maintain excellent diastereoselectivity (>95:5 *dr*). The product obtained by the reaction of α-thiocyanoindanone substituted by the electron-donating group at the 6-position can maintain moderate yields and enantioselectivity (Table 3, entries 3 and 5). However, the enantioselectivities of the products decreased when the 6-fluoro- substituted and 6-bromo-substituted α-thiocyanoindanones reacted with azadiene **1a** (Table 3, entries 2 and 4). The diastereoselectivities and enantioselectivities of the products obtained by reacting of 5-substituted α-thiocyanoindanones with azadiene **1a** were all decreased (Table 3, entries 6–9). From this, we can see that different electron-withdrawing group substitutions of α-thiocyanoindanone generally result in poor reaction effects.

**Figure 4:**
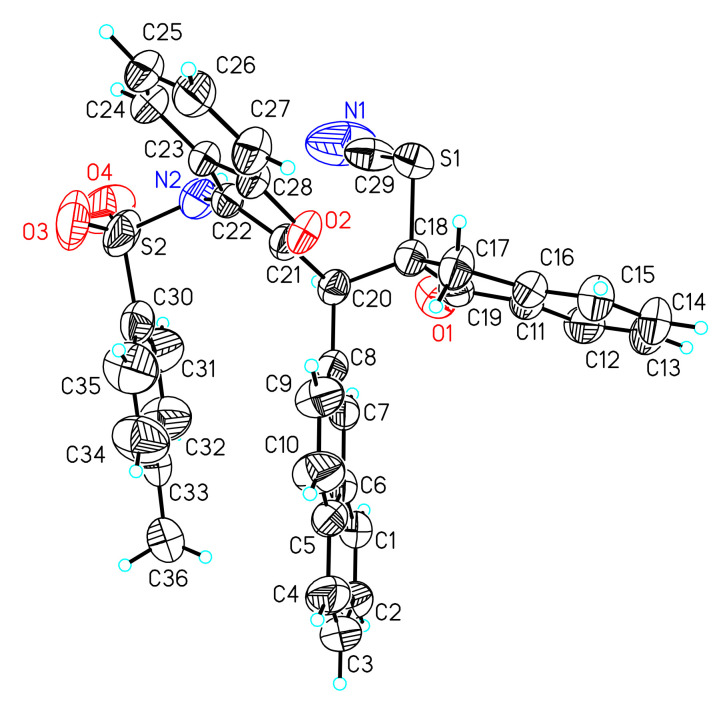
X-ray crystal structure of **3ja**.

The structures of the products obtained by the reaction of azadienes and α-thiocyanoindanones were determined by NMR and HRMS. It is worth mentioning that because the enantioselectivity of **3ja** is not very high (only 90:10 *er*) and cannot improve the enantioselectivity by recrystallization, we only obtained the relative configuration of the product through its single-crystal X-ray analysis (Figure 4) [30]. A similar phenomenon, only relative configuration, was provided owing to low enantioselectivity in Shi’s report [31].

**Scheme 2:**
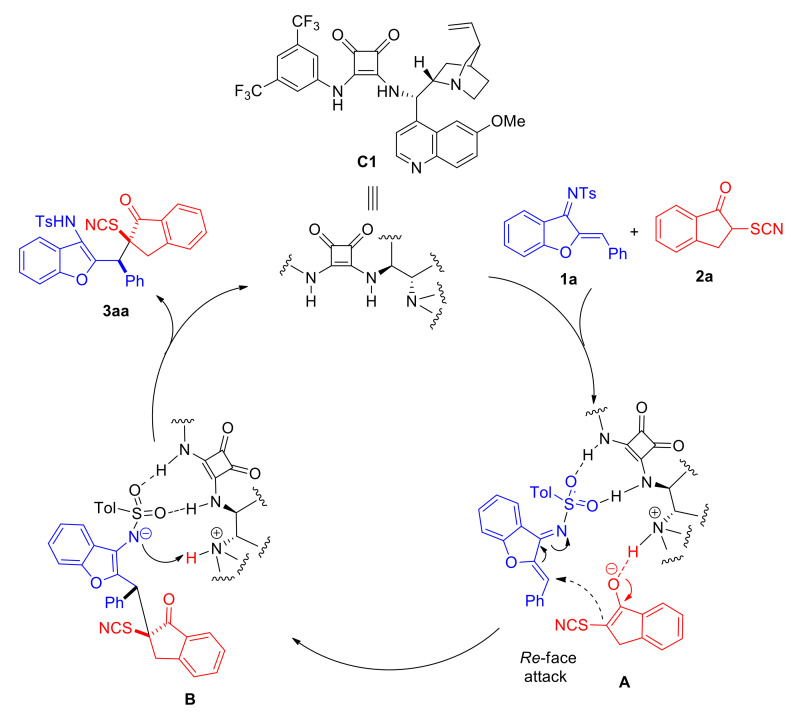
Proposed mechanism for the reaction.

Based on the experimental results and the relative configuration of **3ja**, we proposed a possible reaction pathway for the catalytic asymmetric Michael addition (Scheme 2). The 2-thiocyano-1-indanone **2a** is enolized and deprotonated via quinidine amine of chiral squaramide **C1**. Meanwhile, benzofuran-derived azadiene **1a** is activated by catalyst **C1** via forming hydrogen bonds. This dual activating mode of chiral squaramide **C1** on the two substrates (transition state **A**) led to a stereoselective Michael addition to give an intermediate **B**. Then, an intramolecular protonation of the intermediate **B** occurs to finish the Michael addition and afford product **3aa** with the observed relative configuration.

## 3. Conclusions

All in all, we have successfully established the 1,4-Michael addition reaction of azadienes and α-thiocyanoindanones catalyzed by a chiral bifunctional squaramide, and a series of optically active thiocyano compounds bearing both quaternary and tertiary double stereocenters were obtained. This reaction successfully constructs the thiocyano group into benzofuran derivatives under mild conditions, which provides a new strategy for the development and synthesis of chiral thiocyano compounds. Experimental details for the unsuccessful direct esterification of complex 7, the metathesis reactions and spectroscopic data can be found in the Supplementary Materials.

## 4. Materials and Methods

### 4.1. General Information

Commercially available compounds were used without further purification. Solvents were dried according to standard procedures. Column chromatography was performed with silica gel (200~300 mesh). Melting points were determined with an XT-4 melting-point apparatus and were uncorrected. ^1^H NMR spectra were measured with Bruker Ascend 400 MHz spectrometer; chemical shifts were reported in *δ* (ppm) units relative to tetramethylsilane (TMS) as internal standard. ^13^C NMR spectra were measured at 100 MHz with 400 MHz spectrometer; chemical shifts were reported in ppm relative to tetramethylsilane and referenced to solvent peak (CDCl_3_, *δ* C = 77.00). High-resolution mass spectra (Electron spray ionization) were measured with an Agilent 6520 Accurate-Mass Q-TOF MS system equipped with an electrospray ionization (ESI) source. Optical rotations were measured with a Krüss P8000 polarimeter at the indicated concentration with the units of g/100 mL. Enantiomeric excesses were determined by chiral HPLC analysis using an Agilent 1200 LC instrument with a Daicel Chiralpak IA, IC, or ADH column. ^1^H and ^13^C NMR spectra for newly synthesized compounds, X-ray single crystal data for product **3ja** and copies of the HPLC chromatograms can be found in the Supplementary Materials.

### 4.2. Materials

**1a**‒**1m** were prepared according to the literature report [32,33]. **2a**‒**2i** were prepared according to the literature [29]. The chiral organocatalysts were prepared by following the reported procedures [34,35,36,37].

### 4.3. Procedure for the Synthesis of Racemates of ***3***

To a dried small bottle were added **1** (0.04 mmol), **2** (0.03 mmol), Et_3_N (1.0 mg, 0.01 mmol, 0.2 equiv), and EtOAc (0.5 mL). After stirring at room temperature for 12 h, the reaction mixture was concentrated and directly purified by silica gel column chromatography to afford the racemates of **3**.

### 4.4. Procedure for the Synthesis of Chiral Compounds ***3***

To a dried small bottle were added **1** (0.05 mmol), **2** (0.04 mmol), chiral organocatalyst **C1** (1.3 mg, 0.002 mmol, and 0.05 equiv), and EtOAc (0.5 mL). The mixture was stirred at –10 °C; the reaction mixture was concentrated and directly purified by silica gel column chromatography to afford the desired products **3**.

*4-methyl-N-(2-((1-oxo-2-thiocyanato-2,3-dihydro-1H-inden-2-yl)(phenyl)methyl)benzofuran-3-yl)benzenesulfonamide* (**3aa**)

White solid; Flash column chromatography eluent (ethyl acetate/petroleum ether = 1:5); 16.0 mg (72% yield); m.p. 110–112 °C. HPLC (Daicel Chiralpak IA, *n*-hexane/ethyl acetate = 80:20, flow rate 1.0 mL/min, detection at 254 nm): *t_R_* = 19.0 min (minor), *t_R_* = 22.7 min (major); 90:10 *er*. [α]_D_^25^ = +69.1° (*c* 1.37, CH_2_Cl_2_). ^1^H NMR (400 MHz, CDCl_3_): *δ* 7.77 (d, *J* = 7.6 Hz, 1H, ArH), 7.72 (d, *J* = 8.0 Hz, 1H, ArH), 7.57−7.53 (m, 2H, ArH), 7.46 (d, *J* = 8.4 Hz, 2H, ArH), 7.41−7.30 (m, 4H, ArH), 7.09−7.02 (m, 5H, ArH), 6.86 (d, *J* = 8.4 Hz, 2H, ArH), 6.77 (s, 1H, NH), 5.05(s, 1H, CH), 4.01 (d, *J* = 19.2 Hz, 1H, CH_2_), 3.23 (d, *J* = 18.8 Hz, 1H, CH_2_), 2.21 (s, 3H, CH_3_) ppm. ^13^C NMR (100 MHz, CDCl_3_): *δ*198.4, 153.7, 151.4, 149.7, 143.5, 136.3, 135.9, 134.4, 133.0, 129.4, 129.3, 128.5, 128.4, 127.6, 127.0, 126.1, 125.9, 125.5, 125.4, 123.8, 121.0, 117.1, 111.4, 110.2, 61.7, 44.4, 35.8, 21.5 ppm. HRMS (ESI): *m*/*z* calcd. for C_32_H_2**5**_N_2_O_4_S_2_ [M + H]^+^ 565.1250, found 565.1263.

*N-(2-((4-bromophenyl)(1-oxo-2-thiocyanato-2,3-dihydro-1H-inden-2-yl)methyl)benzofuran-3-yl)-4-methylbenzenesulfonamide* (**3ba**)

White solid; Flash column chromatography eluent (ethyl acetate/petroleum ether = 1:5); 17.5 mg (68% yield); m.p. 84–86 °C. HPLC (Daicel Chiralpak IC, n-hexane/2-propanol = 80:20, flow rate 1.0 mL/min, detection at 254 nm): *t_R_* = 67.0 min (major), *t_R_* = 78.9 min (minor); 89:11 *er*. [α]_D_^25^ = +60° (*c* 0.32, CH_2_Cl_2_).^1^H NMR (400 MHz, CDCl3): δ 7.78 (d, *J* = 8.0 Hz, 1H, ArH), 7.70 (d, *J* = 7.6 Hz, 1H, ArH), 7.61−7.54 (m, 2H, ArH), 7.45 (d, *J* = 8.0 Hz, 2H, ArH), 7.40−7.29 (m, 4H, ArH), 7.16 (d, *J* = 8.4 Hz, 2H, ArH), 6.94–6.85 (m, 5H, ArH + NH), 5.07 (s, 1H, CH), 3.93 (d, J = 18.8 Hz, 1H, CH_2_), 3.23 (d, *J* = 18.8 Hz, 1H, CH_2_), 2.26 (s, 3H, CH_3_) ppm. ^13^C NMR (100 MHz, CDCl_3_): δ 198.2, 153.7, 150.8, 149.5, 143.8, 136.5, 135.9, 133.6, 132.8, 131.6, 131.0, 129.3, 128.7, 127.1, 126.2, 125.7, 125.62, 125.60, 124.0, 122.0, 121.1, 117.4, 111.4, 110.0, 61.4, 43.7, 35.6, 21.6 ppm. HRMS (ESI): *m*/*z* calcd. for C_32_H_24_**^79^**BrN_2_O_4_S_2_ [M + H]^+^ 643.0355, found 643.0348. calcd. for C_32_H_24_^81^BrN_2_O_4_S_2_ [M + H]^+^ 645.0335, found 645.0332.

*4-methyl-N-(2-((1-oxo-2-thiocyanato-2,3-dihydro-1H-inden-2-yl)(p-tolyl)methyl)benzofuran-3-yl)benzenesulfonamide* (**3ca**)

White solid; Flash column chromatography eluent (ethyl acetate/petroleum ether = 1:5); 20.1 mg (87% yield); m.p. 112–114 °C. HPLC (Daicel Chiralpak IA, *n*-hexane/ethyl acetate = 85:15, flow rate 1.0 mL/min, detection at 254 nm): *t_R_* = 41.6 min (minor), *t_R_* = 45.4 min (major); 90:10 *er*. [α]_D_^25^ = +84.7° (*c* 0.78, CH_2_Cl_2_). ^1^H NMR (400 MHz, CDCl_3_): *δ* 7.77 (d, *J* = 7.6 Hz, 1H, ArH), 7.71 (d, *J* = 7.2 Hz, 1H, ArH), 7.58−7.54 (m, 2H, ArH), 7.47 (d, *J* = 8.0 Hz, 2H, ArH), 7.40–7.29 (m, 4H, ArH), 6.91–6.82 (m, 6H, ArH), 6.68 (s, 1H, NH), 5.00 (s, 1H, CH), 4.00 (d, *J* = 18.8 Hz, 1H, CH_2_), 3.22 (d, *J* = 18.8 Hz, 1H, CH_2_), 2.21 (s, 3H, CH_3_), 2.19 (s, 3H, CH_3_) ppm. ^13^C NMR (100 MHz, CDCl_3_): *δ* 198.4, 153.7, 151.7, 149.8, 143.5, 137.3, 136.2, 136.0, 133.0, 131.4, 129.3, 129.2, 129.1, 128.5, 127.1, 126.1, 126.0, 125.5, 125.4, 123.8, 121.0, 116.9, 111.3, 110.2, 61.7, 44.0, 35.8, 21.5, 21.0 ppm. HRMS (ESI): *m*/*z* calcd. for C_33_H_2**7**_N_2_O_4_S_2_ [M + H]^+^ 579.1407, found 579.1403.

*N-(2-((4-fluorophenyl)(1-oxo-2-thiocyanato-2,3-dihydro-1H-inden-2-yl)methyl)benzofuran-3-yl)-4-methylbenzenesulfonamide* (**3da**)

White solid; Flash column chromatography eluent (ethyl acetate/petroleum ether = 1:5); 16.3 mg (70% yield); m.p. 108–110 °C. HPLC (Daicel Chiralpak IC, *n*-hexane/2-propanol = 70:30, flow rate 1.0 mL/min, detection at 254 nm): *t_R_* = 28.5 min (major), *t_R_* = 35.2 min (minor); 88:12 *er*. [α]_D_^25^ = +115.7° (*c* 0.46, CH_2_Cl_2_). ^1^H NMR (400 MHz, CDCl_3_): *δ* 7.78 (d, *J* = 7.6 Hz, 1H, ArH), 7.68 (d, *J* = 8.0 Hz, 1H, ArH), 7.60−7.54 (m, 2H, ArH), 7.47 (d, *J* = 8.4 Hz, 2H, ArH), 7.41−7.29 (m, 4H, ArH), 7.06−7.03 (m, 2H, ArH), 6.90 (d, *J* = 8.0 Hz, 2H, ArH), 6.81 (s, 1H, NH), 6.75−6.71 (m, 2H, ArH), 5.08 (s, 1H, CH), 3.98 (d, *J* = 18.8 Hz, 1H, CH_2_), 3.23 (d, *J* = 18.8 Hz, 1H, CH_2_), 2.24 (s, 3H, CH_3_) ppm. ^13^C NMR (100 MHz, CDCl_3_): *δ* 198.4, 161.9 (d, ^1^*J*_C-F_ = 246.4 Hz), 153.7, 151.1, 149.6, 143.6, 136.5, 136.0, 132.9, 131.2 (d, ^3^*J*_C-F_ = 8.1 Hz), 130.29, 130.26, 129.4, 128.7, 127.1, 126.1, 125.7, 125.6 (d, ^4^*J*_C-F_ = 1.9 Hz), 123.9, 121.0, 117.1, 115.4 (d, ^2^*J*_C-F_ = 21.3 Hz), 111.4, 110.1, 61.5, 43.6, 35.6, 21.4 ppm. HRMS (ESI): *m*/*z* calcd. for C_32_H_24_FN_2_O_4_S_2_ [M + H]^+^ 583.1156, found 583.1143.

*N-(2-((4-chlorophenyl)(1-oxo-2-thiocyanato-2,3-dihydro-1H-inden-2-yl)methyl)benzofuran-3-yl)-4-methylbenzenesulfonamide* (**3ea**)

White solid; Flash column chromatography eluent (ethyl acetate/petroleum ether = 1:5); 15.1 mg (63% yield), m.p. 114–116 °C. HPLC (Daicel Chiralpak IC, *n*-hexane/2-propanol = 80:20, flow rate 1.0 mL/min, detection at 254 nm): *t_R_* = 65.4 min (major), *t_R_* = 75.7 min (minor); 88:12 *er*. [α]_D_^25^ = +60.6° (*c* 1.09, CH_2_Cl_2_). ^1^H NMR (400 MHz, CDCl_3_): δ 7.78 (d, *J* = 7.6 Hz, 1H, ArH), 7.70 (d, *J* = 7.6 Hz, 1H, ArH), 7.61–7.54 (m, 2H, ArH), 7.45 (d, *J* = 8.4 Hz, 2H, ArH), 7.42–7.37 (m, 2H, ArH), 7.35–7.30 (m, 2H, ArH), 7.03–6.97 (m, 4H, ArH), 6.88 (d, *J* = 8.0 Hz, 2H, ArH), 6.79 (s, 1H, NH), 5.07 (s, 1H, CH), 3.94 (d, *J* = 19.2 Hz, 1H, CH_2_), 3.22 (d, *J* = 18.8 Hz, 1H, CH_2_), 2.25 (s, 3H, CH_3_) ppm. ^13^C NMR (100 MHz, CDCl_3_): δ 198.2, 153.8, 151.0, 149.5, 143.8, 136.5, 136.0, 133.8, 133.0, 132.8, 130.8, 129.3, 128.8, 128.6, 127.1, 126.2, 125.8, 125.7, 125.6, 124.0, 121.1, 117.3, 111.4, 110.0, 61.4, 43.6, 35.6, 21.5 ppm. HRMS (ESI): *m*/*z* calcd. for C_32_H_24_ClN_2_O_4_S_2_ [M + H]^+^ 599.0861, found 599.0859.

*4-methyl-N-(2-((1-oxo-2-thiocyanato-2,3-dihydro-1H-inden-2-yl)(4-(trifluoromethyl)phenyl)methyl)benzofuran-3-yl)benzenesulfonamide* (**3fa**)

White solid; Flash column chromatography eluent (ethyl acetate/petroleum ether = 1:5); 15.7 mg (62% yield); m.p. 113–115 °C. HPLC (Daicel Chiralpak IC, *n*-hexane/2-propanol = 85:15, flow rate 1.0 mL/min, detection at 254 nm): *t_R_* = 59.3 min (major), *t_R_* = 70.6 min (minor); 89:11 *er*. [α]_D_^25^ = +52.2° (*c* 0.48, CH_2_Cl_2_).^1^H NMR (400 MHz, CDCl_3_): *δ* 7.79 (d, *J* = 7.6 Hz, 1H, ArH), 7.64−7.55 (m, 3H, ArH), 7.47 (d, *J* = 8.4 Hz, 2H, ArH), 7.41−7.28 (m, 6H, ArH), 7.22 (d, *J* = 8.4 Hz, 2H, ArH), 6.94−6.87 (m, 3H, ArH + NH), 5.21 (s, 1H, CH), 3.93 (d, *J* = 19.2 Hz, 1H, CH_2_), 3.27 (d, *J* = 18.8 Hz, 1H, CH_2_), 2.21 (s, 3H, CH_3_) ppm. ^13^C NMR (100 MHz, CDCl_3_): *δ* 198.0, 153.8, 150.8, 149.3, 143.7, 138.6, 136.6, 136.1, 132.8, 129.85 (q, ^2^*J*_C-F_ = 32.3 Hz), 129.81, 129.4, 128.8, 127.1, 126.2, 125.77, 125.75, 125.71, 125.3 (q, ^3^*J*_C-F_ = 3.7 Hz), 124.1, 123.7 (q, ^1^*J*_C-F_ = 270.5 Hz), 120.9, 117.6, 111.4, 109.9, 61.6, 44.1, 35.7, 21.3 ppm. HRMS (ESI): *m*/*z* calcd. for C_33_H_24_F_3_N_2_O_4_S_2_ [M + H]^+^ 633.1124, found 633.1131.

*4-methyl-N-(2-((1-oxo-2-thiocyanato-2,3-dihydro-1H-inden-2-yl)(4-(oxo-l6-methyl)phenyl)methyl)benzofuran-3-yl)benzenesulfonamide* (**3ga**)

White solid; Flash column chromatography eluent (ethyl acetate/petroleum ether = 1:5); 13.8 mg (58% yield); m.p. 105–107 °C. HPLC (Daicel Chiralpak IC, *n*-hexane/ethyl acetate = 85:15, flow rate 1.0 mL/min, detection at 254 nm): *t_R_* = 68.1 min (major), *t_R_* = 78.5 min (minor); 79:21 *er*. [α]_D_^25^ = +27.1° (*c* 0.51, CH_2_Cl_2_). ^1^H NMR (400 MHz, CDCl_3_): *δ* 7.77 (d, *J* = 7.6 Hz, 1H, ArH), 7.71 (d, *J* = 7.2 Hz, 1H, ArH), 7.58−7.53 (m, 2H, ArH), 7.48 (d, *J* = 8.4 Hz, 2H, ArH), 7.40−7.29 (m, 4H, ArH), 6.96−6.94 (m, 2H, ArH), 6.90 (d, *J* = 8.0 Hz, 2H, ArH ), 6.70 (s, 1H, NH), 6.58−6.54 (m, 2H, ArH), 5.00 (s, 1H, CH), 4.01 (d, *J* = 19.2 Hz, 1H, CH_2_), 3.68 (s, 3H, CH_3_), 3.22 (d, *J* = 19.2 Hz, 1H, CH_2_), 2.22 (s, 3H, CH_3_) ppm. ^13^C NMR (100 MHz, CDCl_3_): *δ* 198.5, 158.8, 153.7, 151.7, 149.8, 143.5, 136.3, 136.0, 133.0, 130.6, 129.4, 128.5, 127.1, 126.4, 126.1, 126.0, 125.5, 125.4, 123.9, 121.0, 116.8, 113.7, 111.3, 110.2, 61.6, 55.0, 43.6, 35.8, 21.4 ppm. HRMS (ESI): *m*/*z* calcd. for C_33_H_26_N_2_O_5_S_2_Na [M + Na]^+^ 617.1175, found 617.1173.

*4-methyl-N-(2-((1-oxo-2-thiocyanato-2,3-dihydro-1H-inden-2-yl)(m-tolyl)methyl)benzofuran-3-yl)benzenesulfonamide* (**3ha**)

White solid; Flash column chromatography eluent (ethyl acetate/petroleum ether = 1:5); 18.3 mg (79% yield); m.p. 102–104 °C. HPLC (Daicel Chiralpak IA, *n*-hexane/ethyl acetate = 80:20, flow rate 1.0 mL/min, detection at 254 nm): *t_R_* = 18.2 min (minor), *t_R_* = 20.2 min (major); 90:10 *er*. [α]_D_^25^ = +64.6° (*c* 0.50, CH_2_Cl_2_). ^1^H NMR (400 MHz, CDCl_3_): *δ* 7.78 (d, *J* = 7.6 Hz, 1H, ArH), 7.74 (d, *J* = 7.6 Hz, 1H, ArH), 7.57−7.54 (m, 2H, ArH), 7.47 (d, *J* = 8.4 Hz, 1H, ArH), 7.41−7.31 (m, 4H, ArH), 6.93−6.82 (m, 6H, ArH), 6.72 (s, 1H, NH), 5.02 (s, 1H, CH), 4.00 (d, *J* = 18.8 Hz, 1H, CH_2_), 3.23 (d, *J* = 19.2 Hz, 1H, CH_2_), 2.20 (s, 3H, CH_3_), 2.15 (s, 3H, CH_3_) ppm. ^13^C NMR (100 MHz, CDCl_3_): *δ* 198.4, 153.7, 151.5, 149.8, 143.4, 138.0, 136.2, 136.0, 134.3, 133.1, 129.9, 129.3, 128.5, 128.4, 128.3, 127.1, 126.4, 126.1, 126.0, 125.5, 125.4, 123.9, 121.1, 117.0, 111.4, 110.2, 61.8, 44.3, 35.9, 21.49, 21.45. ppm. HRMS (ESI): *m*/*z* calcd. for C_33_H_27_N_2_O_4_S_2_ [M + H]^+^ 579.1407, found 579.1419.

*N-(2-((3-bromophenyl)(1-oxo-2-thiocyanato-2,3-dihydro-1H-inden-2-yl)methyl)benzofuran-3-yl)-4-methylbenzenesulfonamide* (**3ia**)

White solid; Flash column chromatography eluent (ethyl acetate/petroleum ether = 1:5); 14.9 mg (58% yield); m.p. 111–113 °C. HPLC (Daicel Chiralpak IA, *n*-hexane/ethyl acetate = 85:15, flow rate 1.0 mL/min, detection at 254 nm): *t*_R_ = 35.0 min (minor), *t*_R_ = 38.9 min (major); 63:37 *er*. [α]_D_^25^ = +51.0° (*c* 0.82, CH_2_Cl_2_). ^1^H NMR (400 MHz, CDCl_3_): *δ* 7.80 (d, *J* = 7.6 Hz, 1H, ArH), 7.73 (d, *J* = 7.6 Hz, 1H, ArH), 7.61−7.56 (m, 2H, ArH), 7.46 (d, *J* = 8.4 Hz, 2H, ArH), 7.43−7.31 (m, 4H, ArH), 7.23−7.18 (m, 2H, ArH), 7.01 (d, *J* = 8.0 Hz, 1H, ArH), 6.94−6.90 (m, 1H, ArH), 6.78 (s, 1H, NH), 5.01 (s, 1H, CH), 3.94 (d, *J* = 18.8 Hz, 1H, CH_2_), 3.23 (d, *J* = 19.2 Hz, 1H, CH_2_), 2.23 (s, 3H, CH_3_) ppm. ^13^C NMR (100 MHz, CDCl_3_): *δ* 198.1, 153.8, 150.5, 149.5, 143.6, 136.7, 136.5, 135.8, 132.9, 132.1, 130.9, 129.9, 129.4, 128.7, 128.1, 127.1, 126.2, 125.68, 125.66, 124.0, 122.5, 121.1, 117.6, 111.5, 109.9, 61.6, 43.9, 35.7, 21.6 ppm. HRMS (ESI): *m*/*z* calcd. for C_32_H_24_^79^BrN_2_O_4_S_2_ [M + H]^+^ 643.0355, found 643.0354. calcd. for C_32_H_24_^81^BrN_2_O_4_S_2_ [M + H]^+^ 645.0335, found 64.0334.

*4-methyl-N-(2-(naphthalen-1-yl(1-oxo-2-thiocyanato-2,3-dihydro-1H-inden-2-yl)methyl)benzofuran-3-yl)benzenesulfonamide* (**3ja**)

White solid; Flash column chromatography eluent (ethyl acetate/petroleum ether = 1:5); 20.4 mg (83% yield); m.p. 118–120 °C. HPLC (Daicel Chiralpak IC, *n*-hexane/ethyl acetate = 85:15, flow rate 1.0 mL/min, detection at 254 nm): *t*_R_ = 52.7 min (major), *t*_R_ = 60.9 min (minor); 90:10 *er*. [α]_D_^25^ = +65.4° (*c* 0.41, CH_2_Cl_2_). ^1^H NMR (400 MHz, CDCl_3_): *δ* 7.85 (d, *J* = 7.6 Hz, 1H, ArH), 7.77 (d, *J* = 7.6 Hz, 1H, ArH), 7.70−7.61 (m, 3H, ArH), 7.52−7.29 (m, 10H, ArH), 7.25 (d, *J* = 7.2 Hz, 1H, ArH), 7.15 (d, *J* = 8.4 Hz, 1H, ArH), 6.87 (s, 1H, NH), 6.53 (d, *J* = 8.0 Hz, 2H, ArH), 5.25 (s, 1H, CH), 4.00 (d, *J* = 18.8 Hz, 1H, CH_2_), 3.24 (d, *J* = 19.2 Hz, 1H, CH_2_), 1.68 (s, 3H, CH_3_) ppm. ^13^C NMR (100 MHz, CDCl_3_): *δ* 198.3, 153.9, 151.2, 149.5, 143.5, 136.3, 135.6, 132.9, 132.8, 132.3, 132.0, 129.1, 129.0, 128.6, 128.2, 128.1, 127.4, 126.9, 126.54, 126.51, 126.4, 126.1, 126.0, 125.6, 125.5, 123.9, 121.4, 117.3, 111.4, 110.3, 61.7, 44.1, 35.7, 20.8 ppm. HRMS (ESI): *m*/*z* calcd. for C_36_H_26_N_2_O_4_S_2_Na [M + Na]^+^ 637.1226, found 637.1228.

*4-methyl-N-(2-((1-oxo-2-thiocyanato-2,3-dihydro-1H-inden-2-yl)(thiophen-2-yl)methyl)benzofuran-3-yl)benzenesulfonamide* (**3ka**)

White solid; Flash column chromatography eluent (ethyl acetate/petroleum ether = 1:5); 17.6 mg (76% yield); m.p. 110–112 °C. HPLC (Daicel Chiralpak IC, *n*-hexane/ethyl acetate = 85:15, flow rate 1.0 mL/min, detection at 254 nm): *t*_R_ = 58.1 min (major), *t*_R_ = 75.1 min (minor); 86:14 *er*. [α]_D_^25^ = +61.5° (*c* 1.03, CH_2_Cl_2_). ^1^H NMR (400 MHz, CDCl_3_): *δ* 7.83 (d, *J* = 7.6 Hz, 1H, ArH), 7.74 (d, *J* = 8.0 Hz, 1H, ArH), 7.60 (t, *J* = 7.6 Hz, 1H, ArH), 7.56−7.53 (m, 3H, ArH), 7.43−7.31 (m, 4H, ArH), 7.00 (dd, *J*_1_ = 5.0 Hz, *J*_2_ = 0.8 Hz, 1H, ArH), 6.95 (d, *J* = 8.0 Hz, 2H, ArH), 6.80 (s, 1H, NH), 6.68–6.66 (m, 1H, ArH), 6.47 (d, *J* = 3.6 Hz, 1H, ArH), 5.22 (s, 1H, CH), 4.05 (d, *J* = 18.8 Hz, 1H, CH_2_), 3.16 (d, *J* = 19.2 Hz, 1H, CH_2_), 2.24 (s, 3H, CH_3_) ppm. ^13^C NMR (100 MHz, CDCl_3_): *δ* 198.1, 153.7, 150.3, 150.0, 143.6, 136.3, 136.0, 135.9, 133.0, 129.4, 128.7, 128.6, 127.1, 126.5, 126.3, 125.9, 125.8, 125.7, 125.6, 124.0, 121.0, 117.1, 111.5, 110.1, 77.3, 77.0, 76.7, 61.2, 40.5, 35.9, 21.6 ppm. HRMS (ESI): *m*/*z* calcd. for C_30_H_23_N_2_O_4_S_3_ [M + H]^+^ 571.0814, found 571.0818.

*4-methyl-N-(2-((1-oxo-2-thiocyanato-2,3-dihydro-1H-inden-2-yl)(pyridin-3-yl)methyl)benzofuran-3-yl)benzenesulfonamide* (**3la**)

White solid; Flash column chromatography eluent (ethyl acetate/petroleum ether = 1:2); 17.6 mg (78% yield); m.p. 104–106 °C. HPLC (Daicel Chiralpak ADH, *n*-hexane/2-propanol = 85:15, flow rate 1.0 mL/min, detection at 254 nm): *t*_R_ = 76.7 min (major), *t*_R_ = 106.2 min (minor); 86:14 *er*. [α]_D_^25^ = +35.3° (*c* 0.68, CH_2_Cl_2_). ^1^H NMR (400 MHz, CDCl_3_): *δ* 8.43–8.37 (m, 2H, ArH), 7.79 (d, *J* = 8.0 Hz, 1H, ArH), 7.60−7.53 (m, 3H, ArH), 7.46 (d, *J* = 8.4Hz, 3H, ArH), 7.40–7.34 (m, 3H, ArH), 7.25 (t, *J* = 7.6 Hz, 1H, ArH), 7.10–7.07 (m, 1H, ArH), 7.00–6.94 (m, 3H, ArH + NH), 5.20 (s, 1H, CH), 4.00 (d, *J* = 19.2 Hz, 1H, CH_2_), 3.29 (d, *J* = 19.2 Hz, 1H, CH_2_), 2.26 (s, 3H, CH_3_) ppm. ^13^C NMR (100 MHz, CDCl_3_): *δ* 197.9, 153.8, 150.3, 149.3, 148.1, 143.9, 138.2, 136.7, 136.0, 132.8, 131.13, 132.11, 129.5, 128.9, 127.2, 126.2, 125.8, 125.7, 125.4, 124.0, 123.5, 120.5, 117.8, 111.5, 109.6, 61.6, 42.8, 35. 9, 21.5 ppm. HRMS (ESI): *m*/*z* calcd. for C_31_H_24_N_3_O_4_S_2_ [M + H]^+^ 566.1203, found 566.1207.

*4-methyl-N-(5-methyl-2-((1-oxo-2-thiocyanato-2,3-dihydro-1H-inden-2-yl)(phenyl)methyl)benzofuran-3-yl)benzenesulfonamide* (**3ma**)

White solid; Flash column chromatography eluent (ethyl acetate/petroleum ether = 1:5); 13.9 mg (60% yield); m.p. 116–118 °C. HPLC (Daicel Chiralpak IC, *n*-hexane/ethyl acetate = 80:20, flow rate 1.0 mL/min, detection at 254 nm): *t_R_* = 20.9 min (minor), *t_R_* = 24.9 min (major); 72:28 *er*. [α]_D_^25^ = +51.8° (*c* 0.84, CH_2_Cl_2_). ^1^H NMR (400 MHz, CDCl_3_): *δ* 7.76 (d, *J* = 7.6 Hz, 1H, ArH), 7.54 (td, *J*_1_ = 7.6 Hz, *J*_2_ = 0.8 Hz, 1H, ArH), 7.48 (d, *J* = 8.4 Hz, 2H, ArH), 7.44−7.42 (m, 2H, ArH), 7.35 (t, *J* = 7.4 Hz, 1H, ArH), 7.30 (d, *J* = 8.0 Hz, 1H, ArH), 7.19 (dd, *J*_1_ = 8.6 Hz, *J*_2_ = 1.4 Hz, 1H, ArH), 7.09−7.00 (m, 5H, ArH), 6.87 (d, *J* = 8.0 Hz, 2H, ArH), 6.64 (s, 1H, NH), 5.01 (s, 1H, CH), 3.99 (d, *J* = 19.2 Hz, 1H, CH_2_), 3.21 (d, *J* = 19.2 Hz, 1H, CH_2_), 2.44 (s, 3H, CH_3_), 2.22 (s, 3H, CH_3_) ppm. ^13^C NMR (100 MHz, CDCl_3_): *δ* 198.4, 152.2, 151.7, 149.7, 143.4, 136.2, 136.1, 134.5, 133.6, 133.0, 129.4, 129.3, 128.5, 128.4, 127.6, 127.1, 126.8, 126.1, 126.0, 125.5, 120.5, 116.8, 110.9, 110.2, 61.8, 44.4, 35.8, 21.5, 21.3 ppm. HRMS (ESI): *m*/*z* calcd. for C_33_H_27_N_2_O_4_S_2_ [M + H]^+^ 579.1407, found 579.1394.

*N-(2-((6-fluoro-1-oxo-2-thiocyanato-2,3-dihydro-1H-inden-2-yl)(phenyl)methyl)benzofuran-3-yl)-4-methylbenzenesulfonamide* (**3ab**)

White solid; Flash column chromatography eluent (ethyl acetate/petroleum ether = 1:5); 14.2 mg (61% yield); m.p. 94–96 °C. HPLC (Daicel Chiralpak IC, *n*-hexane/2-propanol = 80:20, flow rate 1.0 mL/min, detection at 254 nm): *t*_R_ = 63.2 min (minor), *t*_R_ = 80.0 min (major); 78:22 *er*. [α]_D_^25^ = +24.9° (*c* 0.36, CH_2_Cl_2_). ^1^H NMR (400 MHz, CDCl_3_): δ 7.68 (d, *J* = 7.6 Hz, 1H, ArH), 7.55 (d, *J* = 8.0 Hz, 1H, ArH), 7.46 (d, *J* = 8.0 Hz, 2H, ArH), 7.41−7.37 (m, 2H, ArH), 7.33−7.24 (m, 3H, ArH), 7.12−7.01 (m, 5H, ArH), 6.88 (d, *J* = 8.0 Hz, 2H, ArH), 6.71 (s, 1H, NH), 5.04 (s, 1H, CH), 4.00 (d, *J* = 18.8 Hz, 1H, CH_2_), 3.21 (d, *J* = 18.8 Hz, 1H, CH_2_), 2.22 (s, 3H, CH_3_) ppm. ^13^C NMR (100 MHz, CDCl_3_): δ 197.7 (d, ^4^*J*_C-F_ = 3.0 Hz), 162.6 (d, ^1^*J*_C-F_ = 249.0 Hz), 153.7, 151.2, 145.2 (d, *^4^J*_C-F_ = 2.0 Hz), 143.5, 135.9, 134.8 (d, ^3^*J*_C-F_ = 7.6 Hz), 134.2, 129.5, 129.3, 128.5, 127.8, 127.6 (d, ^3^*J*_C-F_ = 8.0 Hz), 127.1, 125.8, 125.5, 124.1 (d, ^2^*J*_C-F_ = 23.6 Hz), 123.9, 120.9, 117.2, 111.4, 111.1, 109.9, 62.4, 44.5, 35.4, 21.5 ppm. HRMS (ESI): *m*/*z* calcd. for C_32_H_24_FN_2_O_4_S_2_ [M + H]^+^ 583.1156, found 583.1161.

*4-methyl-N-(2-((6-methyl-1-oxo-2-thiocyanato-2,3-dihydro-1H-inden-2-yl)(phenyl)methyl)benzofuran-3-yl)benzenesulfonamide* (**3ac**)

White solid; Flash column chromatography eluent (ethyl acetate/petroleum ether = 1:5); 15.3 mg (66% yield); m.p. 106–108 °C. HPLC (Daicel Chiralpak IC, *n*-hexane/ethyl acetate = 70:30, flow rate 1.0 mL/min, detection at 254 nm): *t*_R_ = 9.2 min (minor), *t*_R_ = 11.2 min (major); 87:13 *er*. [α]_D_^25^ = +100.7° (*c* 0.47, CH_2_Cl_2_). ^1^H NMR (400 MHz, CDCl_3_): *δ* 7.73 (d, *J* = 7.6 Hz, 1H, ArH), 7.55 (d, *J* = 8.4 Hz, 2H, ArH), 7.46 (d, *J* = 8.0 Hz, 2H, ArH), 7.41−7.30 (m, 3H, ArH), 7.19 (d, *J* = 8.0 Hz, 1H, ArH), 7.10−7.00 (m, 5H, ArH), 6.86 (d, *J* = 8.4 Hz, 2H, ArH), 6.70 (s, 1H, NH), 5.02 (s, 1H, CH), 3.94 (d, *J* = 18.8 Hz, 1H, CH_2_), 3.16 (d, *J* = 18.8 Hz, 1H, CH_2_), 2.35 (s, 3H, CH_3_), 2.21 (s, 3H, CH_3_) ppm. ^13^C NMR (100 MHz, CDCl_3_): *δ* 198.4, 153.7, 151.5, 147.1, 143.4, 138.7, 137.6, 136.0, 134.5, 133.1, 129.43, 129.36, 128.4, 127.6, 127.0, 126.0, 125.8, 125.41, 125.38, 123.9, 121.0, 117.0, 111.3, 110.3, 62.0, 44.3, 35.5, 21.5, 21.0 ppm. HRMS (ESI): *m*/*z* calcd. for C_33_H_27_N_2_O_4_S_2_ [M + H]^+^ 579.1407, found 579.1396.

*N-(2-((6-bromo-1-oxo-2-thiocyanato-2,3-dihydro-1H-inden-2-yl)(phenyl)methyl)benzofuran-3-yl)-4-methylbenzenesulfonamide* (**3ad**)

White solid; Flash column chromatography eluent (ethyl acetate/petroleum ether = 1:5); 13.6 mg (53% yield); m.p. 198–200 °C. HPLC (Daicel Chiralpak IC, *n*-hexane/ethyl acetate = 80:20, flow rate 1.0 mL/min, detection at 254 nm): *t*_R_ = 20.7 min (minor), *t*_R_ = 22.9 min (major); 70:30 *er*. [α]_D_^25^ = +35.5° (*c* 0.45, CH_2_Cl_2_). ^1^H NMR (400 MHz, CDCl_3_): *δ* 7.89 (s, 1H, ArH), 7.69−7.63 (m, 2H, ArH), 7.54 (d, *J* = 8.0 Hz, 1H, ArH), 7.46 (d, *J* = 8.4 Hz, 1H, ArH), 7.39 (t, *J* = 7.8 Hz, 1H, ArH), 7.31(t, *J* = 7.6 Hz, 1H, ArH), 7.20 (d, *J* = 8.0 Hz, 1H, ArH), 7.13−7.00 (m, 5H, ArH), 6.88 (d, *J* = 8.0 Hz, 1H, ArH), 6.69 (s, 1H, NH), 5.04 (s, 1H, CH), 3.96 (d, *J* = 19.2 Hz, 1H, CH_2_), 3.19 (d, *J* = 19.2 Hz, 1H, CH_2_), 2.22 (s, 3H, CH_3_) ppm. ^13^C NMR (100 MHz, CDCl_3_): *δ* 197.1, 153.7, 151.2, 148.3, 143.5, 139.1, 136.0, 134.8, 134.2, 129.5, 129.3, 128.6, 128.3, 127.8, 127.6, 127.1, 125.9, 125.5, 123.9, 122.7, 121.0, 117.2, 111.4, 109.8, 62.0, 44.4, 35.6, 21.5 ppm. HRMS (ESI): *m*/*z* calcd. for C_32_H_24_^79^BrN_2_O_4_S_2_ [M + H]^+^ 643.0355, found 643.0361; calcd. for C_32_H_24_^81^BrN_2_O_4_S_2_ [M + H]^+^ 645.0335, found 645.0345.

*N-(2-((6-methyoxy-1-oxo-2-thiocyanato-2,3-dihydro-1H-inden-2-yl)(phenyl)methyl)benzofuran-3-yl)-4-methylbenzenesulfonamide* (**3ae**)

White solid; Flash column chromatography eluent (ethyl acetate/petroleum ether = 1:5); 10.2 mg (43% yield); m.p. 118–120 °C. HPLC (Daicel Chiralpak IA, n-hexane/ethyl acetate = 85:15, flow rate 1.0 mL/min, detection at 254 nm): *t_R_* = 43.3 min (minor), *t_R_* = 67.2 min (major); 86:14 *er*. [α]_D_^25^ = +105.7° (*c* 1.46, CH_2_Cl_2_). ^1^H NMR (400 MHz, CDCl_3_): δ 7.71 (d, *J* = 8.0 Hz, 1H, ArH), 7.55 (d, *J* = 8.4 Hz, 1H, ArH), 7.47−7.45 (m, 2H, ArH), 7.38 (td, *J*_1_ = 7.6 Hz, *J*_2_ = 1.2 Hz, 1H, ArH), 7.32−7.29 (m, 1H, ArH), 7.207.17 (m, 2H, ArH), 7.14−7.01 (m, 6H, ArH), 6.85 (d, *J* = 8.0 Hz, 1H, ArH), 6.78 (s, 1H, NH), 5.05 (s, 1H, CH), 3.94 (d, *J* = 18.8 Hz, 1H, CH_2_), 3.80 (s, 3H, CH_3_), 3.14 (d, *J* = 18.4 Hz, 1H, CH_2_), 2.20 (s, 3H, CH_3_) ppm. ^13^C NMR (100 MHz, CDCl_3_): δ 198.4, 160.0, 153.7, 151.4, 143.4, 142.6, 135.9, 134.4, 134.2, 129.4, 129.3, 128.4, 127.6, 127.0, 126.8, 125.9, 125.8, 125.4, 123.8, 121.0, 117.0, 111.3, 110.3, 106.3, 62.4, 55.6, 44.4, 35.2, 21.5 ppm. HRMS (ESI): *m*/*z* calcd. for C_33_H_27_N_2_O_5_S_2_ [M + H]^+^ 595.1356, found 595.1347.

*N-(2-((5-fluoro-1-oxo-2-thiocyanato-2,3-dihydro-1H-inden-2-yl)(phenyl)methyl)benzofuran-3-yl)-4-methylbenzenesulfonamide* (**3af**)

White solid; Flash column chromatography eluent (ethyl acetate/petroleum ether = 1:5); 10.5 mg (45% yield); m.p. 113–115 °C. HPLC (Daicel Chiralpak IA, *n*-hexane/ethyl acetate = 85:15, flow rate 1.0 mL/min, detection at 254 nm): *t*_R_ = 42.1 min (minor), *t*_R_ = 48.7min (major); 74:26 *er*. [α]_D_^25^ = +65.8° (*c* 1.2, CH_2_Cl_2_). ^1^H NMR (400 MHz, CDCl_3_): *δ* 7.78 (dd, *J*_1_ = 8.4 Hz, *J*_2_ = 5.2 Hz, 1H, ArH), 7.68 (d, *J* = 7.6 Hz, 1H, ArH), 7.55 (d, *J* = 8.4 Hz, 1H, ArH), 7.46 (d, *J* = 8.0 Hz, 2H, ArH), 7.39 (td, *J*_1_ = 7.8 Hz, *J*_2_ = 1.2 Hz, 1H, ArH), 7.32−7.29 (m, 1H, ArH), 7.11−7.02 (m, 6H, ArH), 6.87 (d, *J* = 8.0 Hz, 2H, ArH), 6.71 (s, 1H, NH), 5.05 (s, 1H, CH), 4.03 (d, *J* = 19.2 Hz, 1H, CH_2_), 3.25 (d, *J* = 19.2 Hz, 1H, CH_2_), 2.21 (s, 3H, CH_3_) ppm. ^13^C NMR (100 MHz, CDCl_3_): *δ* 196.7, 167.9 (d, ^1^*J*_C-F_ = 258.3 Hz), 153.7, 152.8 (d, ^3^*J*_C-F_ = 10.6 Hz), 151.2, 143.5, 135.9, 134.2, 129.4, 129.3, 128.5, 128.0 (d, ^3^*J*_C-F_ = 10.6 Hz), 127.8, 127.1, 125.8, 125.5, 123.9, 120.9, 117.2, 117.0, 113.0 (d, ^2^*J*_C-F_ = 22.9 Hz), 111.4, 109.9, 62.0, 44.5, 35.9, 21.5 ppm. HRMS (ESI): *m*/*z* calcd. for C_32_H_24_FN_2_O_4_S_2_ [M + H]^+^ 583.1156, found 583.1148.

*N-(2-((5-chloro-1-oxo-2-thiocyanato-2,3-dihydro-1H-inden-2-yl)(phenyl)methyl)benzofuran-3-yl)-4-methylbenzenesulfonamide* (**3ag**)

White solid; Flash column chromatography eluent (ethyl acetate/petroleum ether = 1:5); 14.6 mg (61% yield); m.p. 128–130 °C. HPLC (Daicel Chiralpak IA, *n*-hexane/2-propanol = 90:10, flow rate 1.0 mL/min, detection at 254 nm): *t*_R_ = 42.9 min (minor), *t*_R_ = 67.4 min (major); 62:38 *er*. [α]_D_^25^ = +24.0° (*c* 0.56, CH_2_Cl_2_). ^1^H NMR (400 MHz, CDCl_3_): *δ* 7.69 (t, *J* = 8.0 Hz, 2H, ArH), 7.55 (d, *J* = 8.0 Hz, 1H, ArH), 7.46 (d, *J* = 8.4 Hz, 2H, ArH), 7.41−7.37 (m, 1H, ArH), 7.34−7.30 (m, 3H, ArH), 7.12-7.00 (m, 6H, ArH), 6.87 (d, *J* = 8.0 Hz, 2H, ArH), 6.63 (s, 1H, NH), 5.04 (s, 1H, CH), 4.00 (d, *J* = 19.2 Hz, 1H, CH_2_), 3.21 (d, *J* = 19.2 Hz, 1H, CH_2_), 2.22 (s, 3H, CH_3_) ppm. ^13^C NMR (100 MHz, CDCl_3_): *δ* 197.1, 153.7, 151.23, 151.15, 143.5, 143.0, 136.0, 134.2, 131.5, 129.5, 129.3, 128.6, 127.8, 127.1, 126.6, 126.4, 125.9, 125.5, 123.9, 121.0, 117.2, 111.4, 109.8, 61.8, 44.4, 35.6, 21.6 ppm. HRMS (ESI): *m*/*z* calcd. for C_32_H_24_ClN_2_O_4_S_2_ [M + H]^+^ 599.0861, found 599.0851.

*N-(2-((5-bromo-1-oxo-2-thiocyanato-2,3-dihydro-1H-inden-2-yl)(phenyl)methyl)benzofuran-3-yl)-4-methylbenzenesulfonamide* (**3ah**)

White solid; Flash column chromatography eluent (ethyl acetate/petroleum ether = 1:5); 16.5 mg (64% yield); m.p. 106–108 °C. HPLC (Daicel Chiralpak IA, *n*-hexane/ethyl acetate = 70:30, flow rate 1.0 mL/min, detection at 254 nm): *t*_R_ = 26.7 min (minor), *t*_R_ = 39.4 min (major); 69:31 *er*. [α]_D_^25^ = +44.8° (*c* 0.88, CH_2_Cl_2_). ^1^H NMR (400 MHz, CDCl_3_): *δ* 7.67 (d, *J* = 7.6 Hz, 1H, ArH), 7.62 (d, *J* = 8.4 Hz, 1H, ArH), 7.55 (d, *J* = 8.4 Hz, 1H, ArH), 7.50−7.45 (m, 4H, ArH), 7.39 (td, *J*_1_ = 7.6 Hz, *J*_2_ = 1.2 Hz, 1H, ArH), 7.33-7.29 (m, 1H, ArH), 7.12−7.01 (m, 5H, ArH), 6.87 (d, *J* = 8.4 Hz, 2H, ArH), 6.68 (s, 1H, NH), 5.05 (s, 1H, CH), 4.01 (d, *J* = 19.2 Hz, 1H, CH_2_), 3.22 (d, *J* = 18.8 Hz, 1H, CH_2_), 2.21 (s, 3H, CH_3_) ppm. ^13^C NMR (100 MHz, CDCl_3_): *δ* 197.3, 153.7, 151.2, 151.1, 143.5, 135.9, 134.2, 132.3, 132.0 131.9, 129.4, 129.3, 128.6, 127.8, 127.1, 126.6, 125.8, 125.5, 123.9, 120.9, 117.2, 111.4, 109.8, 61.8, 44.4, 35.6, 21.5 ppm. HRMS (ESI): *m*/*z* calcd. for C_32_H_24_^79^BrN_2_O_4_S_2_ [M + H]^+^ 643.0355, found 643.0359; calcd. for C_32_H_24_^81^BrN_2_O_4_S_2_ [M + H]^+^ 645.0335, found 645.0342.

*4-methyl-N-(2-((1-oxo-5-(oxo-l6-methyl)-2-thiocyanato-2,3-dihydro-1H-inden-2-yl)(phenyl)methyl)benzofuran-3-yl)benzenesulfonamide* (**3ai**)

White solid; Flash column chromatography eluent (ethyl acetate/petroleum ether = 1:5); 15.2 mg (64% yield); m.p. 103–105 °C. HPLC (Daicel Chiralpak IA, *n*-hexane/ethyl acetate = 70:30, flow rate 1.0 mL/min, detection at 254 nm): *t*_R_ = 10.1 min (minor), *t*_R_ = 12.7 min (major); 85:15 *er*. [α]_D_^25^ = +78.6° (*c* 1.12, CH_2_Cl_2_). ^1^H NMR (400 MHz, CDCl_3_): *δ* 7.74 (d, *J* = 7.2 Hz, 1H, ArH), 7.68 (d, *J* = 8.4 Hz, 1H, ArH), 7.54 (d, *J* = 8.4 Hz, 1H, ArH), 7.46 (d, *J* = 8.4 Hz, 2H, ArH), 7.41−7.36 (m, 1H, ArH), 7.36−7.30 (m, 1H, ArH), 7.10−7.02 (m, 5H, ArH), 6.87−6.84 (m, 3H, ArH), 6.75−6.71 (m, 2H, ArH + NH), 5.00 (s, 1H, CH), 3.96 (d, *J* = 18.8 Hz, 1H, CH_2_), 3.81 (s, 3H, CH_3_), 3.19 (d, *J* = 18.8 Hz, 1H, CH_2_), 2.20 (s, 3H, CH_3_) ppm. ^13^C NMR (100 MHz, CDCl_3_): *δ* 196.5, 166.5, 153.7, 153.0, 151.4, 143.4, 135.9, 134.5, 129.4, 129.3, 128.4, 127.6, 127.3, 127.0, 126.1, 126.0, 125.4, 123.8, 121.0, 117.1, 116.6, 111.3, 110.4, 109.3, 62.3, 55.7, 44.5, 35.9, 21.5 ppm. HRMS (ESI): *m*/*z* calcd. for C_33_H_27_N_2_O_5_S_2_ [M + H]^+^ 595.1356, found 595.1364.

## Figures and Tables

**Table 1:** Establishment of optimal reaction conditions for azadiene and α-thiocyanoindanone ^*a*^.

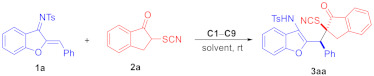					
Entry	Solvent	Catalyst	Yield *^b^* (%)	*dr ^c^*	*er* *^d^*
1	CH_2_Cl_2_	**C1**	77	90:10	83:17
2	CH_2_Cl_2_	**C2**	50	89:11	80:20
3	CH_2_Cl_2_	**C3**	54	86:14	73:27
4	CH_2_Cl_2_	**C4**	63	88:12	76:24
5	CH_2_Cl_2_	**C5**	53	86:14	71:29
6	CH_2_Cl_2_	**C6**	24	86:14	67:33
7	CH_2_Cl_2_	**C7**	53	93:7	81:19
8	CH_2_Cl_2_	**C8**	35	84:16	56:44
9	CH_2_Cl_2_	**C9**	49	88:12	67:33
10	PhMe	**C1**	88	>95:5	81:19
11	THF	**C1**	56	>95:5	82:18
12	MTBE	**C1**	45	>95:5	78:22
13	EtOAc	**C1**	68	>95:5	86:14
14	1,4-dioxane	**C1**	54	>95:5	86:14
15	PhCF_3_	**C1**	82	>95:5	80:20
16	CHCl_3_	**C1**	60	>95:5	79:21
17	PhCl	**C1**	67	>95:5	82:18
18	CH_3_CN	**C1**	28	>95:5	81:19
19	acetone	**C1**	46	>95:5	79:21
20	ClCH_2_CH_2_Cl	**C1**	83	76:24	81:19
21	CCl_4_	**C1**	81	>95:5	70:30
22	dimethylbenzene	**C1**	36	76:24	63:37
23	MeOH	**C1**	24	>95:5	45:55
24 *^e^*	EtOAc	**C1**	41	89:11	86:14
25 *^f^*	EtOAc	**C1**	36	79:21	63:37
26 *^g^*	EtOAc	**C1**	80	71:29	87:13
27 *^h^*	EtOAc	**C1**	54	90:10	86:14
**28 *^i^***	**EtOAc**	**C1**	**72**	**>95**:**5**	**90**:**10**
29 *^j^*	EtOAc	**C1**	86	93:7	85:15
30 *^k^*	EtOAc	**C1**	49	92:8	84:16

*^a^* Unless otherwise specified, reactions were carried out with **1a** (0.05 mmol), **2a** (0.04 mmol) and catalyst (5 mol%) in solvent (0.5 mL) at room temperature. *^b^* Isolated yield after column chromatography purification. *^c^* Determined by ^1^H NMR analysis. *^d^* Enantiometric ratio was determined by HPLC analysis. *^e^* 2.5 mol% catalyst was used. *^f^* 1 mol% catalyst was used. *^g^* 10 mol% catalyst was used. *^h^* The reaction was performed at 0 °C. *^i^* The reaction was performed at −10 °C. *^j^* The reaction was performed at −20 °C. *^k^* The reaction was carried out with **1a** (0.05 mmol), **2a** (0.04 mmol), and **C1** (5 mol%) in EtOAc (2 mL) at −10 °C.

**Table 2:** Substrate scope of azadienes ^*a*^.

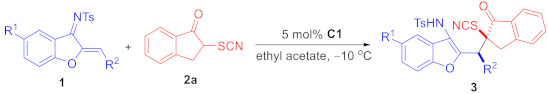					
Entry	R^1^/R^2^ (1)	3	Yield *^b^* (%)	*dr ^c^*	*er ^d^*
1	H/Ph (**1a**)	**3aa**	72	>95:5	90:10
2	H/*p*-BrC_6_H_4_ (**1b**)	**3ba**	68	80:20	89:11
3	H/*p*-MeC_6_H_4_ (**1c**)	**3ca**	87	>95:5	90:10
4	H/*p*-FC_6_H_4_ (**1d**)	**3da**	70	85:15	88:12
5	H/*p*-ClC_6_H_4_ (**1e**)	**3ea**	63	80:20	88:12
6	H/*p*-CF_3_C_6_H_4_ (**1f**)	**3fa**	62	75:25	89:11
7	H/*p*-MeOC_6_H_4_ (**1g**)	**3ga**	58	>95:5	79:21
8	H/*m*-MeC_6_H_4_ (**1h**)	**3ha**	79	>95:5	90:10
9	H/*m*-BrC_6_H_4_ (**1i**)	**3ia**	58	70:30	63:37
10	H/2-naphthyl (**1j**)	**3ja**	83	>95:5	90:10
11	H/2-thienyl (**1k**)	**3ka**	76	86:14	86:14
12	H/2-pyridinyl (**1l**)	**3la**	78	>95:5	86:14
13	Me/Ph (**1m**)	**3ma**	60	>95:5	72:28

*^a^* Unless otherwise noted, all reactions were carried out using **1** (0.05 mmol), **2a** (0.04 mmol), and catalyst **C1** (5 mol%) in solvent (0.5 mL) at −10 °C. *^b^* Isolated yield after column chromatography purification.^*c*^ The *dr* values were determined by ^1^H NMR analysis. *^d^* Enantiometric ratio was determined by HPLC analysis.

**Table 3:** Substrate scope of α-thiocyanoindanones ^*a*^.

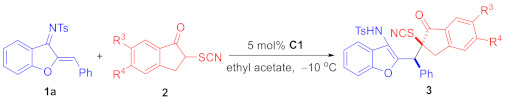					
Entry	R^3^/R^4^ (2)	3	Yield *^b^* (%)	*dr ^c^*	*er ^d^*
1	H/H (**2a**)	**3aa**	72	>95:5	90:10
2	F/H (**2b**)	**3ab**	61	>95:5	78:22
3	Me/H (**2c**)	**3ac**	66	>95:5	87:13
4	Br/H (**2d**)	**3ad**	53	>95:5	70:30
5	MeO/H(**2e**)	**3ae**	43	87:13	86:14
6	H/F (**2f**)	**3af**	45	86:14	74:26
7	H/Cl (**2g**)	**3ag**	61	84:16	62:38
8	H/Br (**2h**)	**3ah**	64	83:17	69:31
9	H/MeO (**2i**)	**3ai**	64	83:17	85:15

*^a^* Unless otherwise noted, all reactions were carried out using **1** (0.05 mmol), **2a** (0.04 mmol), and catalyst C1 (5 mol%) in ethyl acetate (0.5 mL) at −10 °C. *^b^* Isolated yield after column chromatography purification. ^*c*^ The *dr* values were determined by ^1^H NMR analysis. *^d^* Enantiometric ratio was determined by HPLC analysis.

## Data Availability

Not applicable.

## Supplementary Materials

Spectroscopic data (^1^H and ^13^C NMR) and chiral HPLC chromatograms for all new compounds **3**.

## Sample Availability

Samples of the compounds **3** are available from the authors.

## References

[1] Dawood K.M. (2013). Benzofuran derivatives: A patent review. Expert Opin. Ther. Pat..

[2] Radadiya A., Shah A. (2015). Bioactive benzofuran derivatives: An insight on lead developments, radioligands and advances of the last decade. Eur. J. Med. Chem..

[3] Masubuchi M., Kawasaki K., Ebiike H., Ikeda Y., Tsujii S., Sogabe S., Fujii T., Sakata K., Shiratori Y., Aoki Y. (2001). Design and synthesis of novel benzofurans as new class of antifungal agents targeting fungal N-myristoyltransferase. Part 1. Bioorg. Med. Chem. Lett..

[4] Nevagi R.J., Dighe S.N. (2015). Biological and medicinal significance of benzofuran. Eur. J. Med. Chem..

[5] Hiremathad A., Patil M.R., Chethana K.R., Chand K., Santos M.A., Keri R.S. (2015). Benzofuran: An emerging scaffold for antimicrobial agents. RSC Adv..

[6] Mitsui C., Soeda J., Miwa K., Tsuji H., Takeya J., Nakamura E. (2012). Naphtho[2,1-b:6,5-b’]difuran: A versatile motif available for solution-processed single-crystal organic field-effect transistors with high hole mobility. J. Am. Chem. Soc..

[7] Bickoff E.M., Booth A.N., Lyman R.L., Livingston A.L., Thompson C.R., Deeds F. (1958). Coumestrol, a new estrogen isolated from forage crops. Science.

[8] Xu Z., Zhao S.J., Lv Z.S., Feng L.S., Wang Y.L., Zhang F., Bai L.Y., Deng J.L. (2019). Benzofuran derivatives and their anti-tubercular, anti-bacterial activities. Eur. J. Med. Chem..

[9] Gu Z., Zhou J., Jiang G.F., Zhou Y.G. (2018). Synthesis of chiral γ-aminophosphonates through the organocatalytic hydrophosphonylation of azadienes with phosphites. Org. Chem. Front..

[10] Gu Z., Xie J.J., Jiang G.F., Zhou Y.G. (2018). Catalytic asymmetric conjugate addition of tritylthiol to azadienes with a bifunctional organocatalyst. Asian J. Org. Chem..

[11] Lin W., Zhang C., Xu W., Cheng Y.Y., Li P.F., Li W.J. (2019). Organocatalytic asymmetric Michael addition of rhodanines to azadienes for assembling of sulfur-containing tetrasubstituted carbon stereocenters. Adv. Synth. Catal..

[12] Lin W., Lin X., Cheng Y.Y., Chang X.Y., Zhou S., Li P.F., Li W.J. (2019). Organocatalytic enantioselective direct vinylogous Michael addition of γ-substituted deconjugate butenolides to azadienes. Org. Chem. Front..

[13] Li S.F., Zhang C., Cheng Y.Y., Jia Q.F., Li W.J., Liu K., Li P.F. (2020). Enantioselective construction of vicinal sulfur-functionalized quaternary and tertiary stereocenters via organocatalytic Michael addition of 5H-Thiazol-4-ones to 1-azadienes. Asian J. Org. Chem..

[14] Yan J.Z., Li X.P., Chen Y.Z., Li Y., Chen W.W., Zhan R.T., Huang H.C. (2020). Organocatalytic 1,4-addition of azadienes with 3-homoacyl coumarins toward highly enantioenriched benzofuran coumarin skeletons. J. Org. Chem..

[15] Brown P.D., Morra M.J. (1993). Fate of ionic thiocyanate (SCN–) in soil. J. Agric. Food Chem..

[16] Garson M.J., Simpson J.S. (2004). Marine isocyanides and related natural products—Structure, biosynthesis and ecology. Nat. Prod. Rep..

[17] Elhalem E., Bailey B.N., Docampo R., Ujváry I., Szajnman B.H., Rodriguez J. (2002). Design, synthesis, and biological evaluation of aryloxyethyl thiocyanate derivatives against trypanosoma cruzi. J. Med. Chem..

[18] Dutta S., Abe H., Aoyagi S., Kibayashi C., Gates K.S. (2005). DNA Damage by fasicularin. J. Am. Chem. Soc..

[19] Yasman, Edrada A.R., Wray V., Proksch P. (2003). New 9-thiocyanatopupukeanane sesquiterpenes from the nudibranch phyllidia varicosa and its sponge-prey axinyssa aculeate. J. Nat. Prod..

[20] Chandler J.D., Day B.J. (2012). Thiocyanate: A potentially useful therapeutic agent with host defense and antioxidant properties. Biochem. Pharmacol..

[21] Zhu N., Wang F., Chen P.H., Ye J.X., Liu G.S. (2015). Copper-catalyzed intermolecular trifluoromethylazidation and trifluoromethylthiocyanation of allenes: Efficient access to CF_3_-containing allyl azides and thiocyanates. Org. Lett..

[22] Chen Q., Lei Y.J., Wang Y.F., Wang C., Wang Y.N., Xu Z.Q., Wang H., Wang R. (2017). Direct thiocyanation of ketene dithioacetals under transition-metal-free conditions. Org. Chem. Front..

[23] Yang H., Duan X.H., Zhao J.F., Guo L.N. (2015). Transition-metal-free tandem radical thiocyanooxygenation of olefinic amides: A new route to SCN-containing heterocycles castanheiro. Org. Lett..

[24] Castanheiro T., Gulea M., Donnard M., Suffert J. (2014). Practical access to aromatic thiocyanates by CuCN-mediated direct aerobic oxidative cyanation of thiophenols and diaryl disulfides. Eur. J. Org. Chem..

[25] Zhu D., Chang D.H., Shi L. (2015). Transition-metal-free cross-coupling of thioethers with aryl(cyano)iodonium triflates: A facile and efficient method for the one-pot synthesis of thiocyanates. Chem. Commun..

[26] Teng F., Yu J.T., Yang H.T., Jiang Y., Cheng J. (2014). Copper-catalyzed cyanation of disulfides by azobisisobutyronitrile leading to thiocyanates. J. Chem. Commun..

[27] Xu Q., Zhang L.Y., Feng G.F., Jin C.G. (2019). Progress on the synthesis and applications of thiocyanates. Chin. J. Org. Chem..

[28] Castanherio T., Suffert J., Donnard M., Gulea M. (2016). Recent advances in the chemistry of organic thiocyanates. Chem. Soc. Rev..

[29] Yu L., Wu X.Y., Kim J.M., Vaithiyanathan V., Liu Y.D., Tan Y., Qin W.L., Song C.E., Yan H.L. (2017). Asymmetric synthesis of 2-thiocyanato-2-(1-aminoalkyl)-substituted 1-tetralones and 1-indanones with tetrasubstituted carbon stereogenic centers via cooperative cation-binding catalysis. Adv. Synth. Catal..

[30] CCDC 2100823 Contains the Supplementary Crystallographic Data for This Paper These Data Can Be Obtained Free of Charge via. http://www.ccdc.cam.ac.uk/conts/retrieving.html.

[31] Wang C.S., Li T.Z., Cheng Y.C., Zhou J., Mei G.J., Shi F. (2019). Catalytic asymmetric [4 + 1] cyclization of benzofuran-derived azadienes with 3-chlorooxindoles. J. Org. Chem..

[32] Shi Z.G., Tong Q.J., Leong W.W.Y., Zhong G.F. (2012). [4+2] Annulation of vinyl ketones initiated by a phosphine-catalyzed Aza-Rauhut–Currier reaction: A practical access to densely functionalized tetrahydropyridines. Chem. Eur. J..

[33] Rong Z.Q., Wang M., Chow C.H.E., Zhao Y. (2016). A catalyst-enabled diastereodivergent Aza-Diels–Alder reaction: Complementarity of N-Heterocyclic Carbenes and chiral amines. Chem. Eur. J..

[34] Zhu Y., Malerich J.P., Rawal V.H. (2010). Squaramide-catalyzed enantioselective Michael addition of diphenyl phosphite to nitroalkenes. Angew. Chem. Int. Ed..

[35] Yang W., Du D.M. (2010). Highly enantioselective Michael addition of nitroalkanes to chalcones using chiral squaramides as hydrogen bonding organocatalysts. Org. Lett..

[36] Yang W., Du D.M. (2011). Chiral squaramide-catalyzaed highly enanioselective Michael addition of 2-hydroxy-1,4-naphthoquinones to nitroalkenes. Adv. Synth. Catal..

[37] Vakulya B., Varga S., Csampai A., Soós T. (2005). Highly enantioselective conjugate addition of nitromethane to chalconesusing bifunctional cinchona organocatalysts. Org. Lett..

