# Supplementary material for: Asymmetric 1,4-Michael Addition Reaction of Azadienes with α-Thiocyanoindanones Catalyzed by Bifunctional Chiral Squaramide
Source: Molecules. 2021 Aug 25;26(17):5146. doi: 10.3390/molecules26175146 (PMC8456331; doi:10.3390/molecules26175146)
Supplement: Supplementary file 1 [file molecules-26-05146-s001.zip › molecules-1347265-supplementary.pdf]

Asymmetric 1,4-Michael addition reaction of azadienes with α-thiocyanindanones catalyzed by bifunctional chiral squareamide

Xiao-Yan Dong, Da-Ming Du*

[1]*School of Chemistry and Chemical Engineering, Beijing Institute of Technology, 5 South Zhongguancun Street, Beijing 100081, People's Republic of China*

E-mail: dudm@bit.edu.cn

Supporting Information

Contents

1. Copies of ^{1}H and ^{13}C NMR spectra of new compounds…………….................................S1
2. X-ray single-crystal data for product **3ja**……………………..…..……………………… .S43
3. Copies of HPLC chromatograms……………………..…..…………………………….. S44

1. Copies of ^{1}H and ^{13}C NMR spectra of new compounds

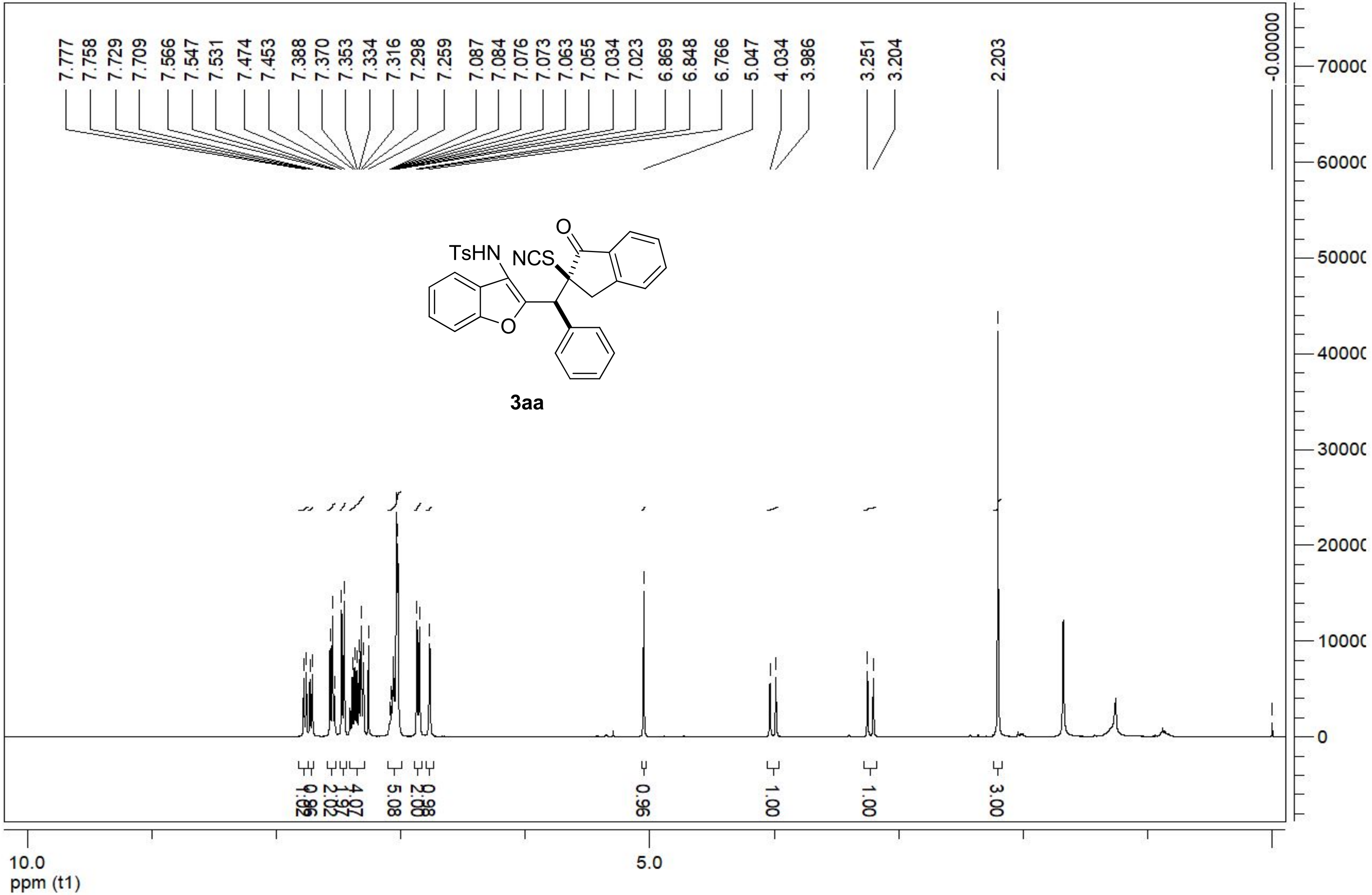

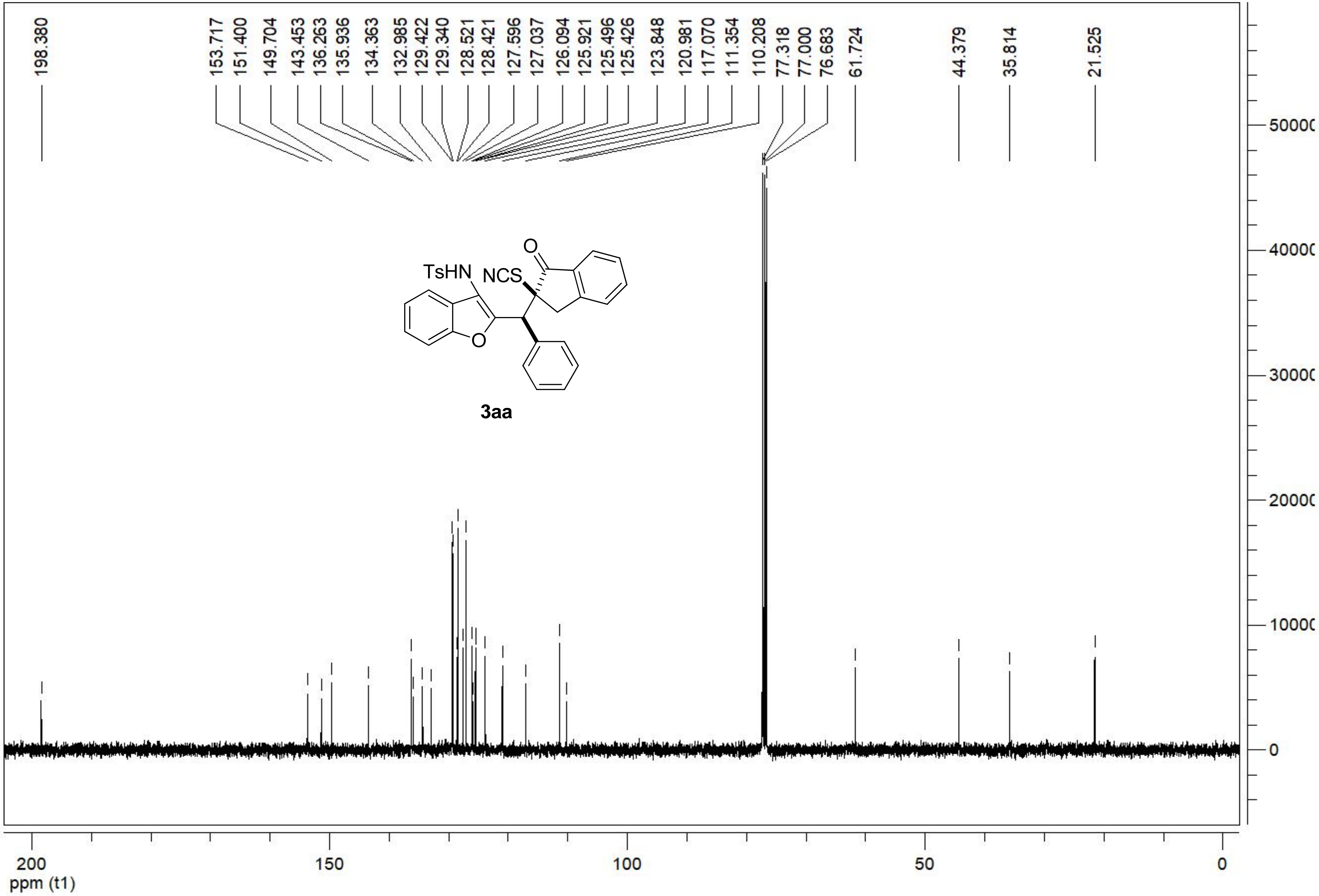
198.380
153.717
151.400
149.704
143.453
136.263
135.936
134.363
132.985
129.422
129.340
128.521
128.421
127.596
127.037
126.094
125.921
125.496
125.426
123.848
120.981
117.070
111.354
110.208
77.318
77.000
76.683
61.724
44.379
35.814
21.525
TsHN
NCS
O
O
3aa
50000
40000
30000
20000
10000
0
200
150
100
50
0
ppm (t1)

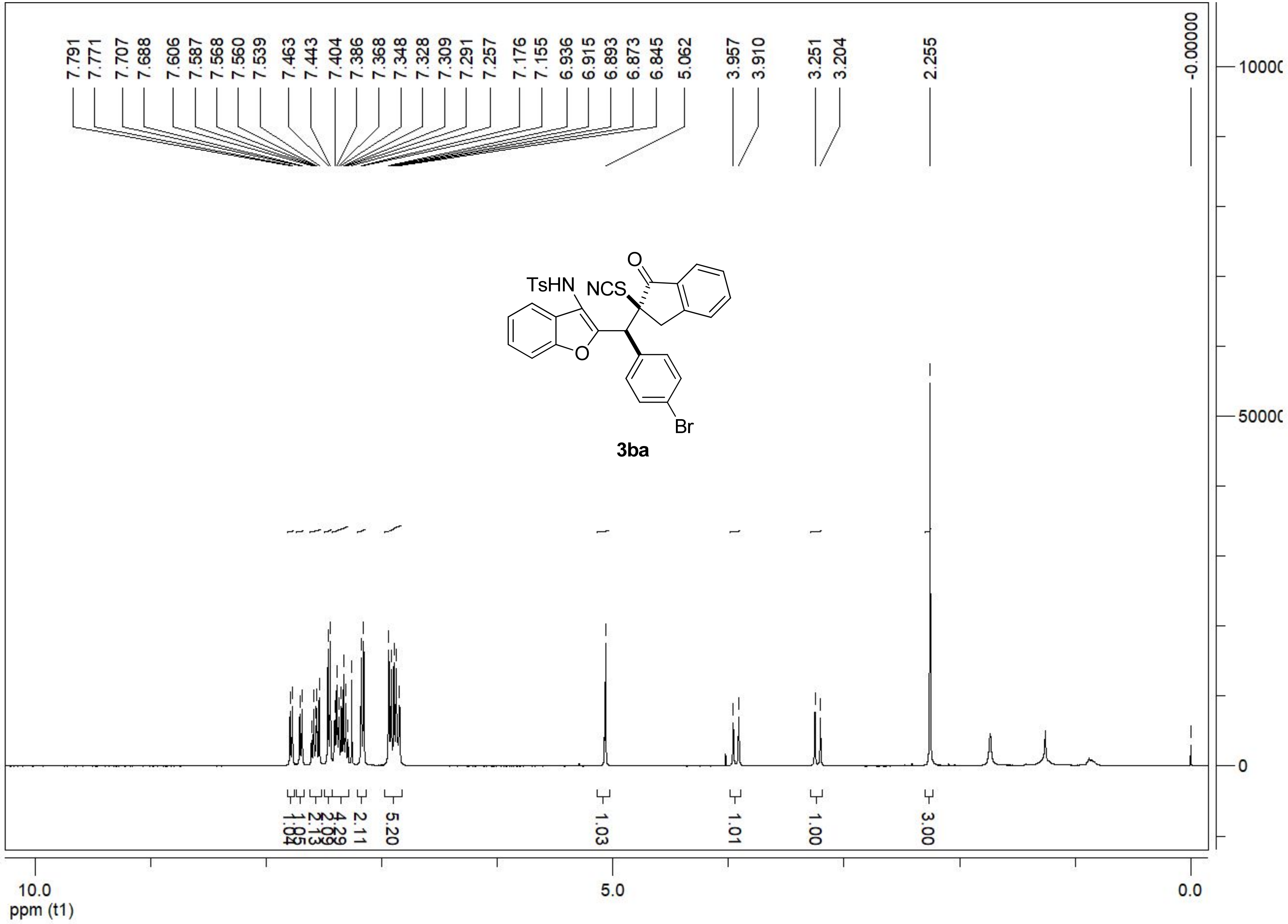
7.791 7.771 7.707 7.688 7.606 7.587 7.568 7.560 7.539 7.463 7.443 7.404 7.386 7.368 7.348 7.328 7.309 7.291 7.257 7.176 7.155 6.936 6.915 6.893 6.873 6.845 5.062
3.957 3.910
3.251 3.204
2.255
-0.00000
TsHN
NCS
O
O
Br
3ba
1.04 1.05 2.13 2.09 4.29 2.11 5.20
1.03
1.01
1.00
3.00
10.0
ppm (t1)
5.0
0.0
0
50000
100000

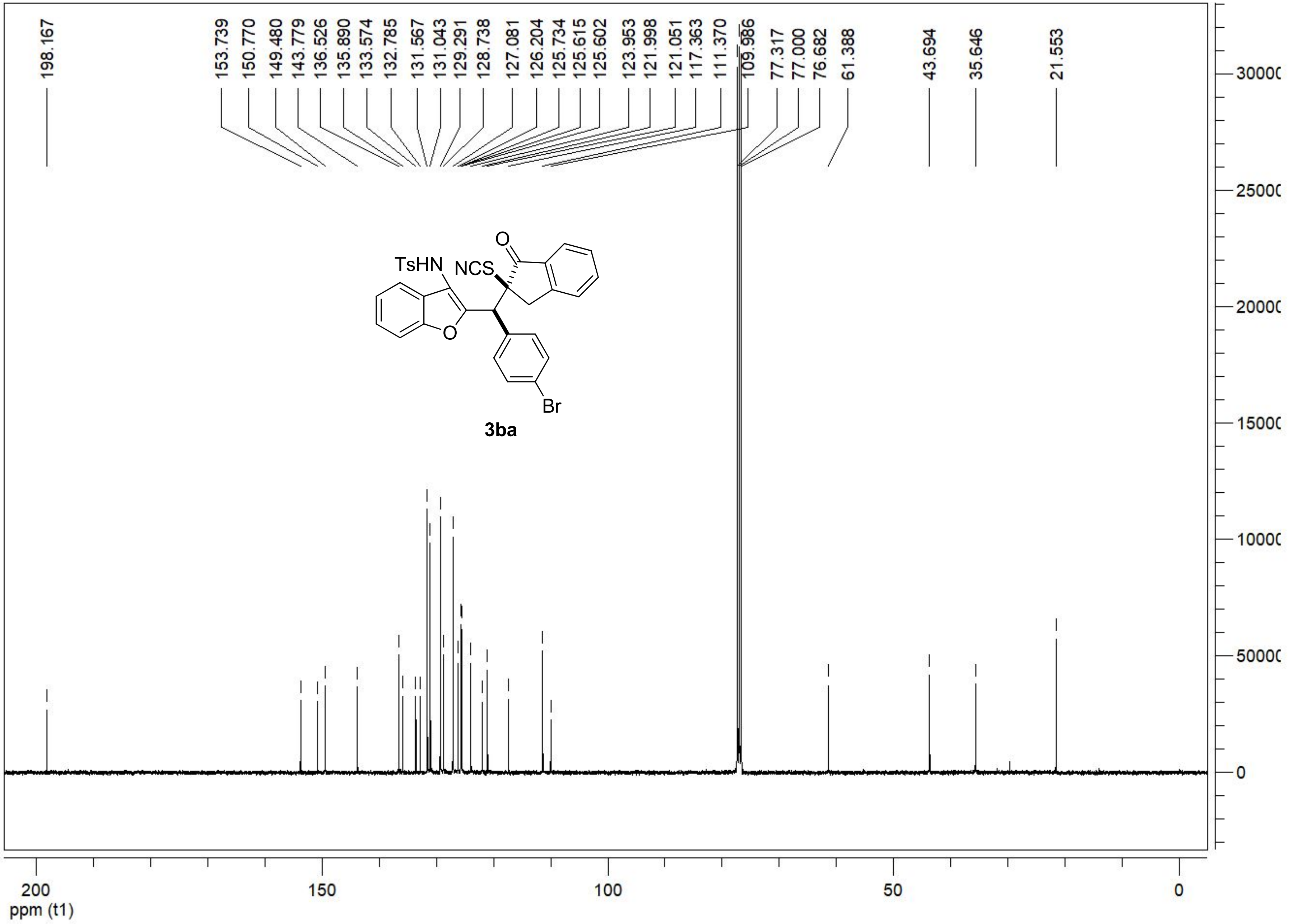
198.167
153.739
150.770
149.480
143.779
136.526
135.890
133.574
132.785
131.567
131.043
129.291
128.738
127.081
126.204
125.734
125.615
125.602
123.953
121.998
121.051
117.363
111.370
109.586
77.317
77.000
76.682
61.388
43.694
35.646
21.553
TsHN
NCS
O
Br
3ba
200
150
100
50
0
ppm (t1)
30000
25000
20000
15000
10000
50000
0

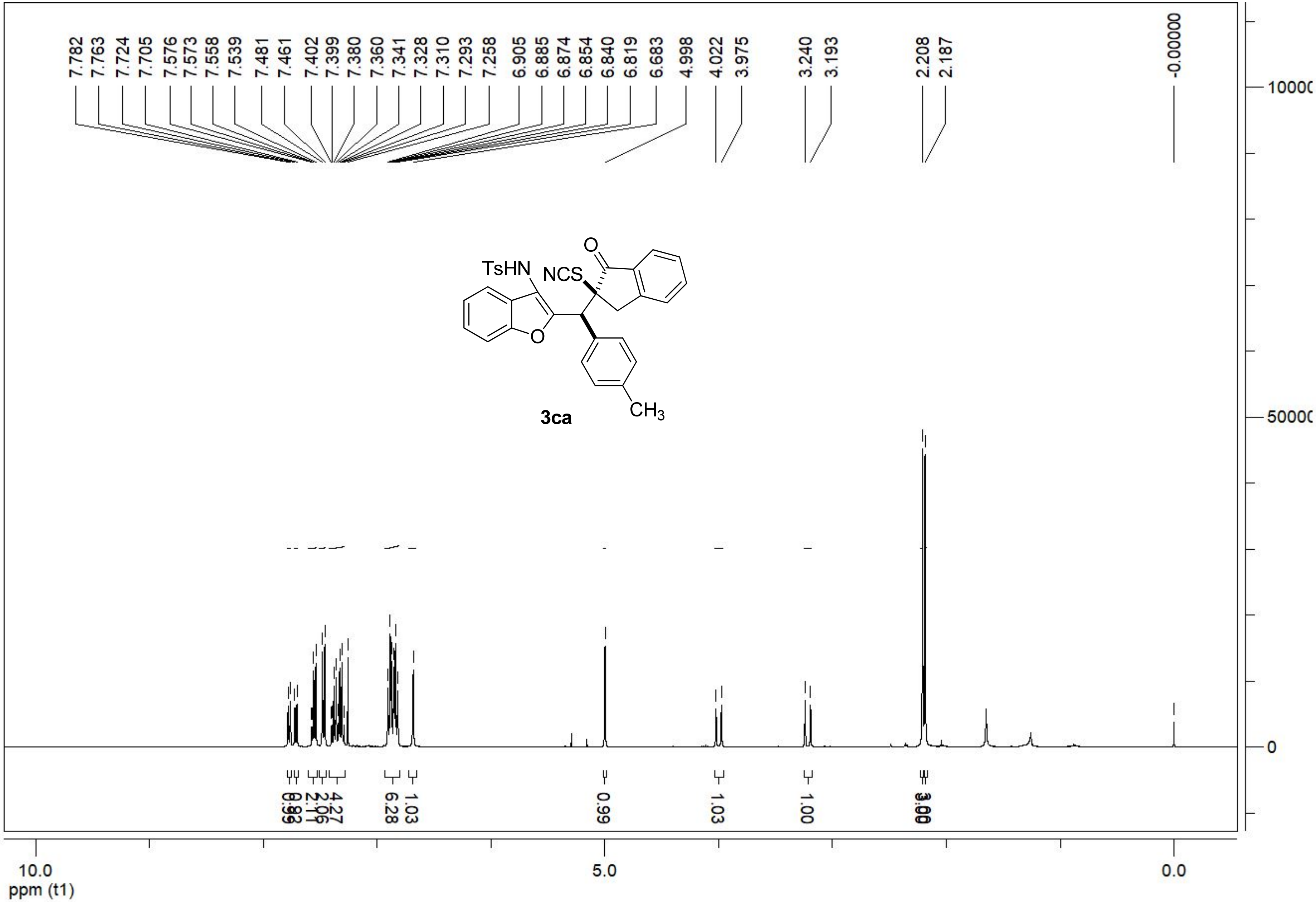
7.782
7.763
7.724
7.705
7.576
7.573
7.558
7.539
7.481
7.461
7.402
7.399
7.380
7.360
7.341
7.328
7.310
7.293
7.258
6.905
6.885
6.874
6.854
6.840
6.819
6.683
4.998
4.022
3.975
3.240
3.193
2.208
2.187
-0.00000
TsHN
NCS
O
CH3
3ca
2.11
2.06
4.27
6.28
1.03
0.99
1.03
1.00
10000
50000
0
10.0
5.0
0.0
ppm (t1)

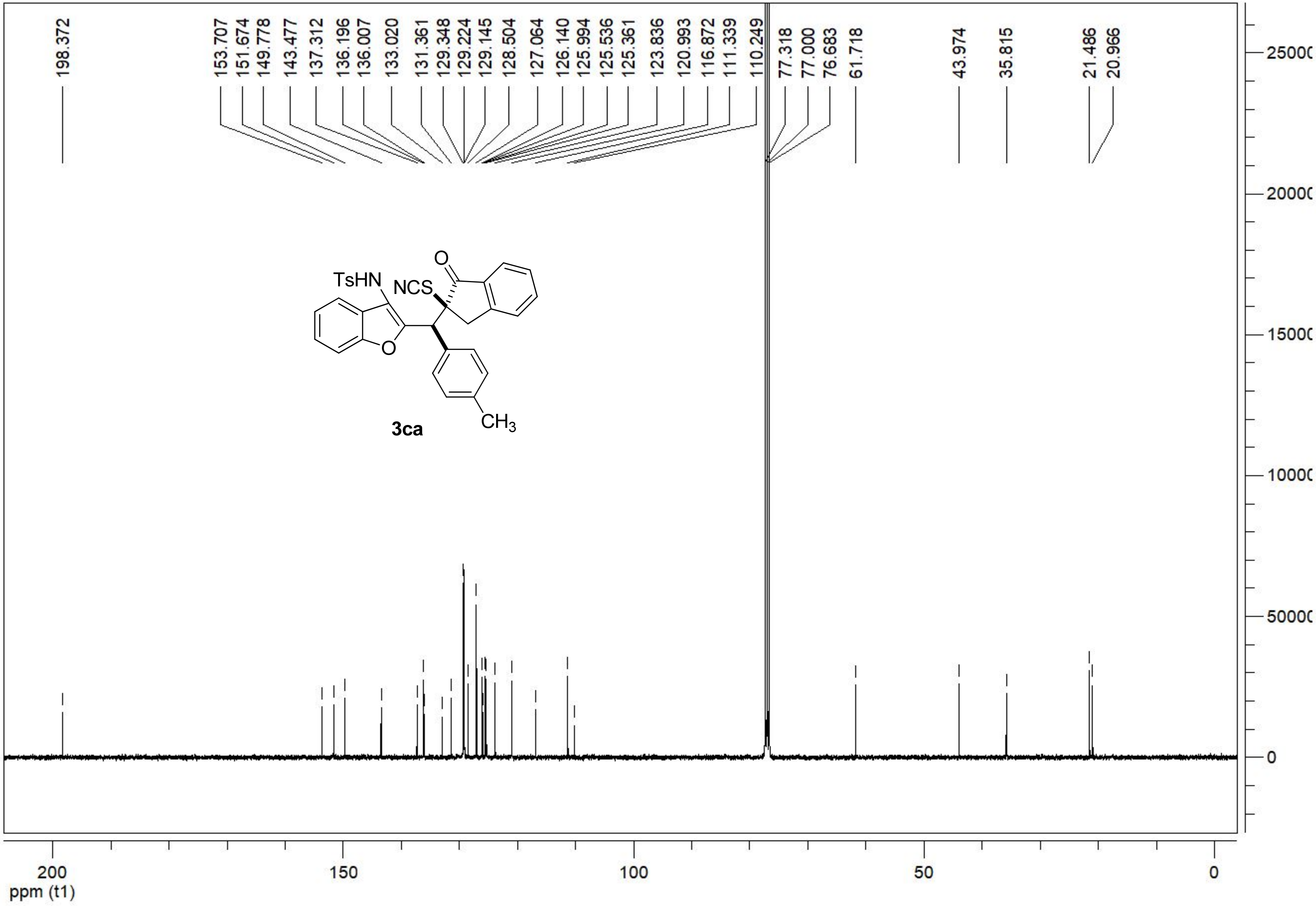
198.372
153.707
151.674
149.778
143.477
137.312
136.196
136.007
133.020
131.361
129.348
129.224
129.145
128.504
127.064
126.140
125.994
125.536
125.361
123.836
120.993
116.872
111.339
110.249
77.318
77.000
76.683
61.718
43.974
35.815
21.486
20.966
TsHN
NCS
O
CH3
3ca
25000
20000
15000
10000
50000
0
200
150
100
50
0
ppm (t1)

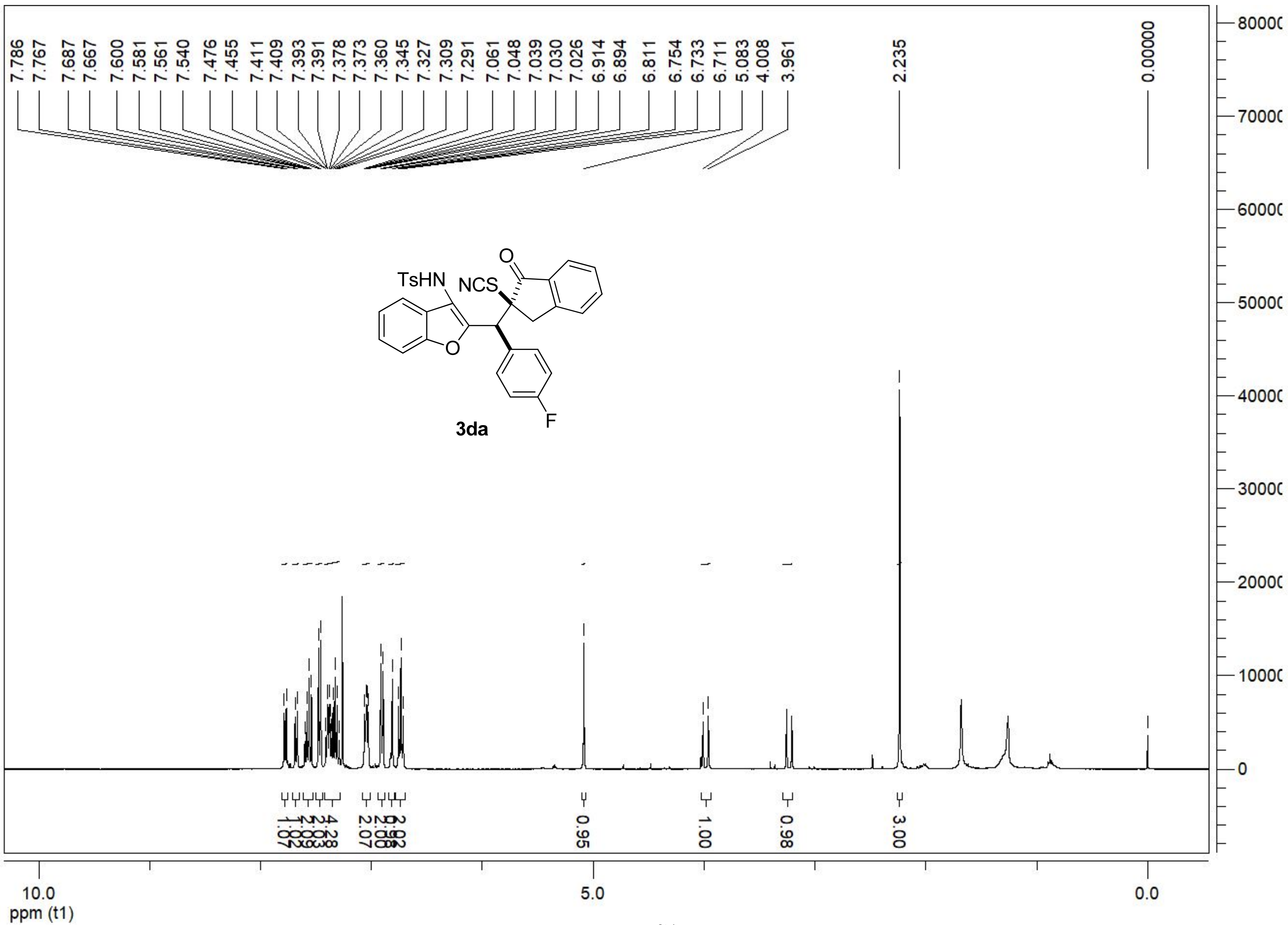
7.786
7.767
7.687
7.667
7.600
7.581
7.561
7.540
7.476
7.455
7.411
7.409
7.393
7.391
7.378
7.373
7.360
7.345
7.327
7.309
7.291
7.061
7.048
7.039
7.030
7.026
6.914
6.894
6.811
6.754
6.733
6.711
5.083
4.008
3.961
2.235
0.00000
TsHN
NCS
O
O
F
3da
4.28
2.07
0.95
1.00
0.98
3.00
10.0
5.0
0.0
ppm (t1)
80000
70000
60000
50000
40000
30000
20000
10000
0

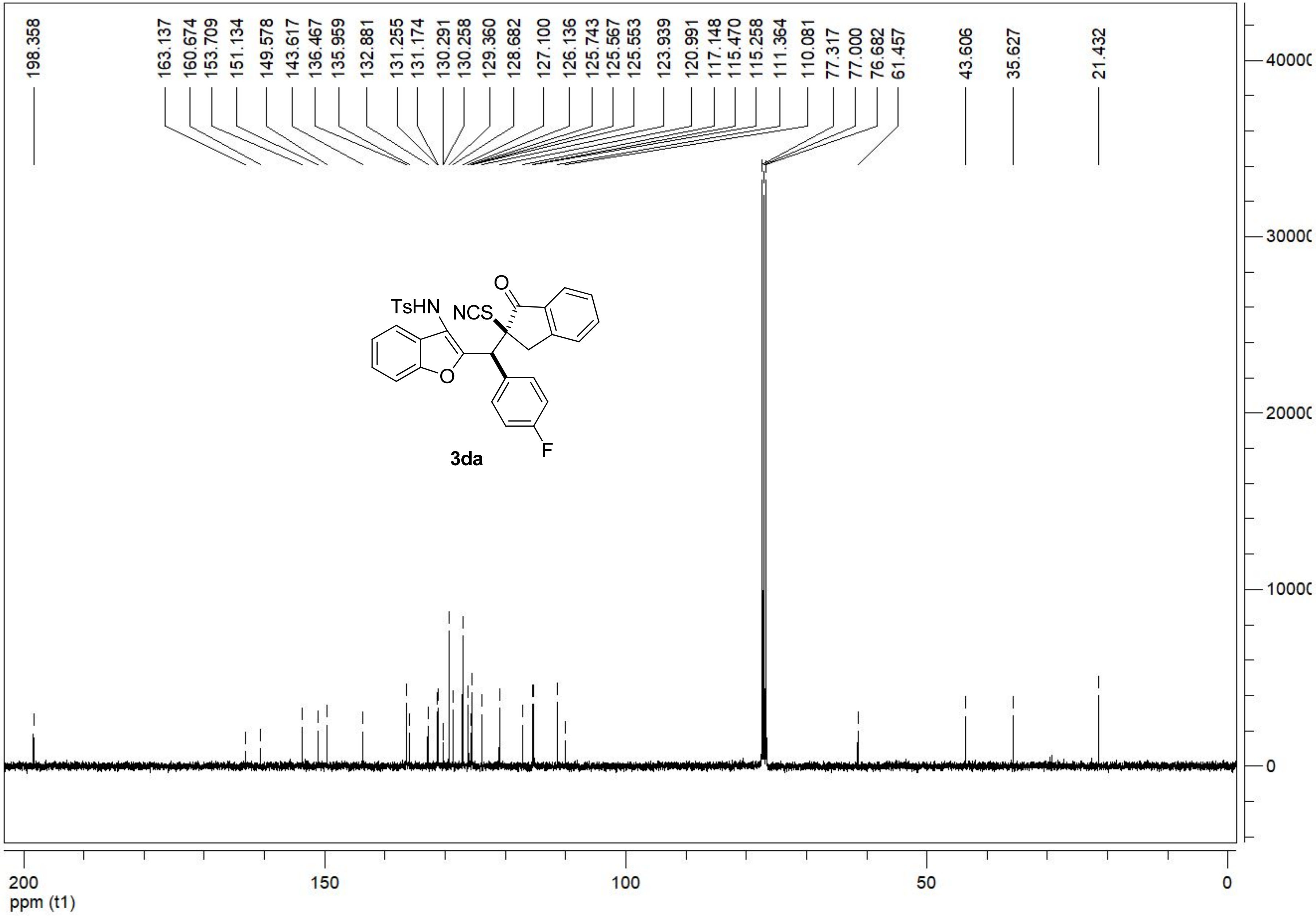
198.358
163.137
160.674
153.709
151.134
149.578
143.617
136.467
135.959
132.881
131.255
131.174
130.291
130.258
129.360
128.682
127.100
126.136
125.743
125.567
125.553
123.939
120.991
117.148
115.470
115.258
111.364
110.081
77.317
77.000
76.682
61.457
43.606
35.627
21.432
TsHN
NCS
O
O
F
3da
40000
30000
20000
10000
0
200
150
100
50
0
ppm (t1)

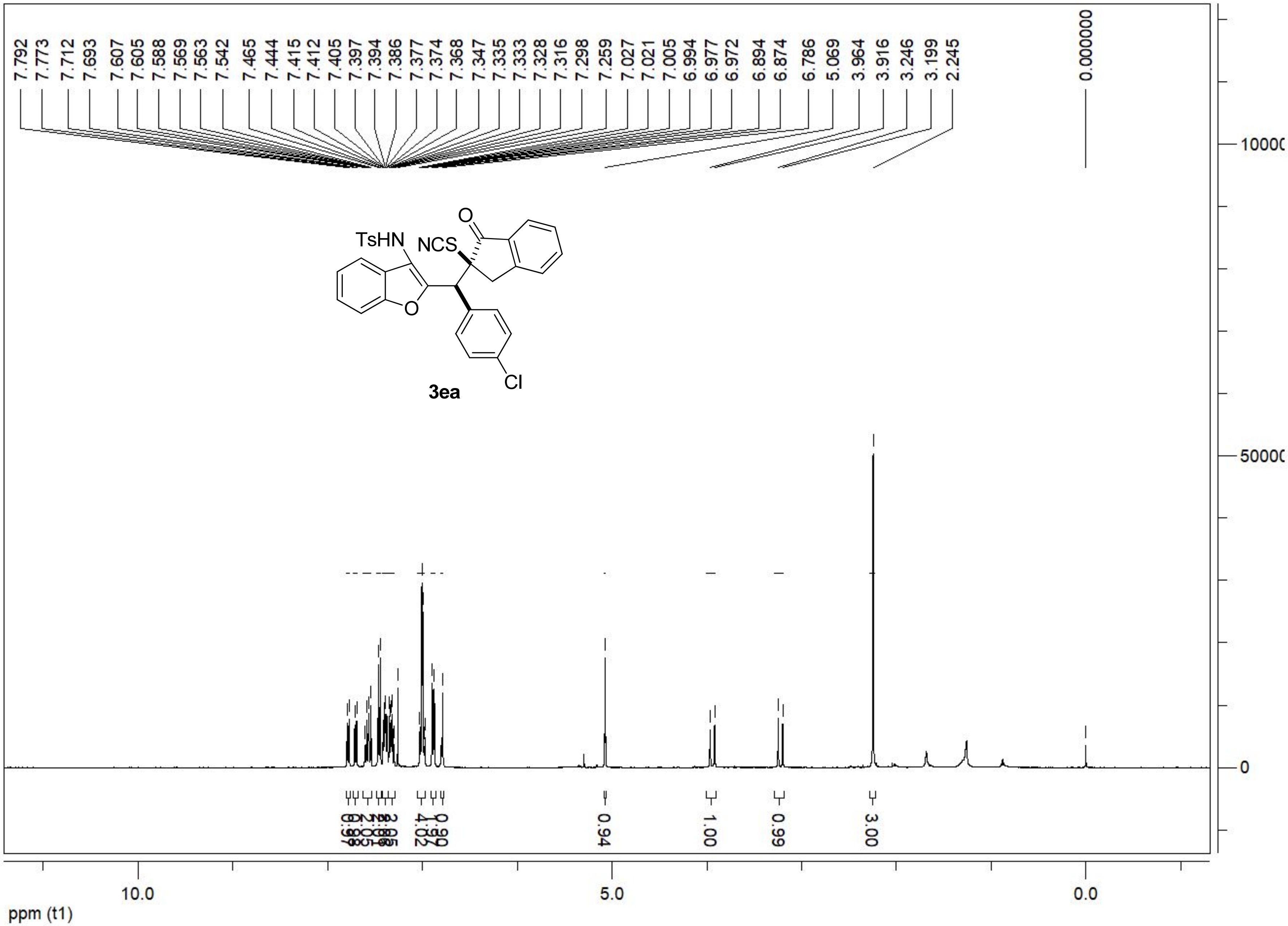
7.792
7.773
7.712
7.693
7.607
7.605
7.588
7.569
7.563
7.542
7.465
7.444
7.415
7.412
7.405
7.397
7.394
7.386
7.377
7.374
7.368
7.347
7.335
7.333
7.328
7.316
7.298
7.259
7.027
7.021
7.005
6.994
6.977
6.972
6.894
6.874
6.786
5.069
3.964
3.916
3.246
3.199
2.245
0.000000
TsHN
NCS
O
O
Cl
3ea
4.02
0.94
1.00
0.99
3.00
10.0
5.0
0.0
ppm (t1)
10000
50000
0

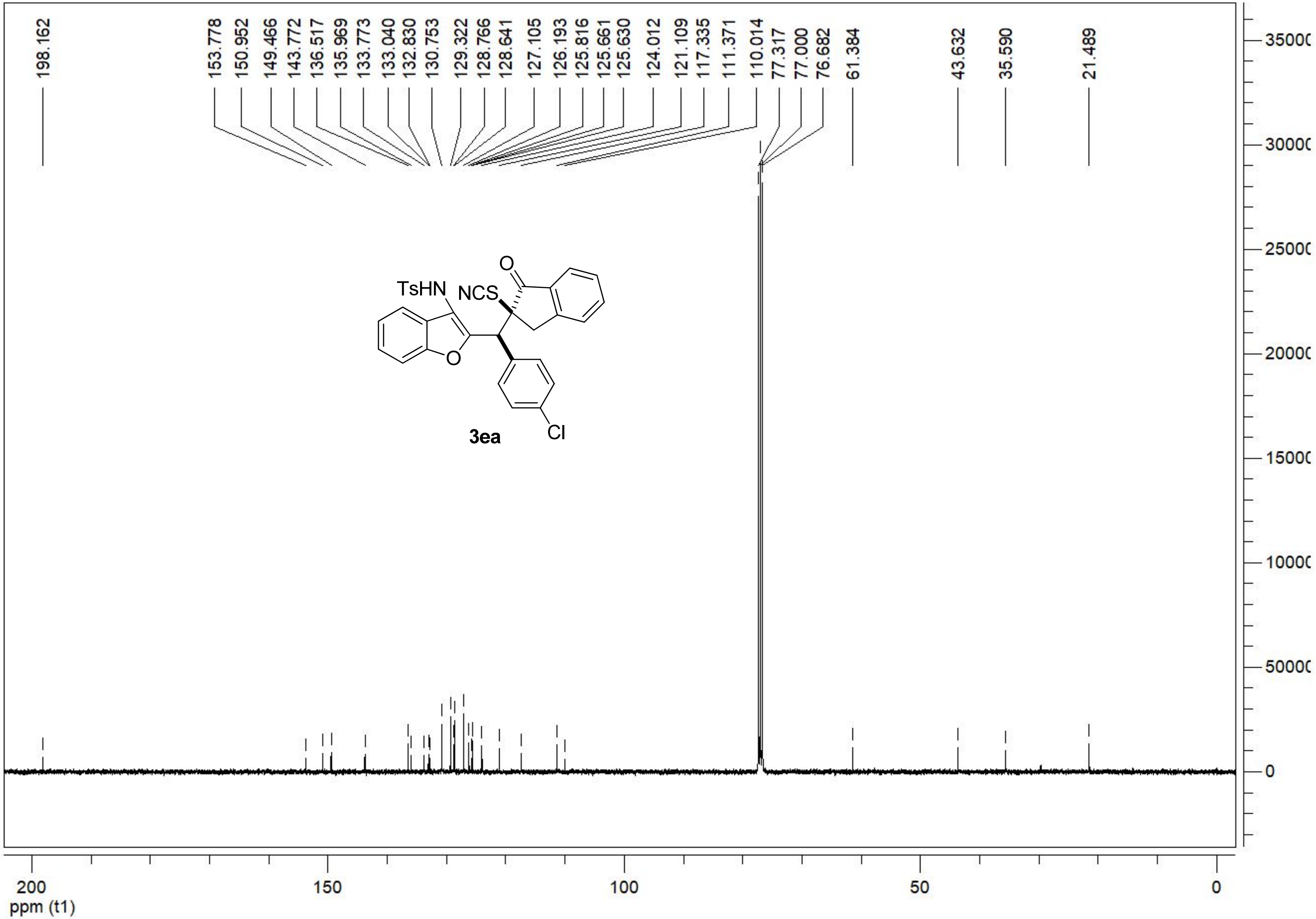
198.162
153.778
150.952
149.466
143.772
136.517
135.969
133.773
133.040
132.830
130.753
129.322
128.766
128.641
127.105
126.193
125.816
125.661
125.630
124.012
121.109
117.335
111.371
110.014
77.317
77.000
76.682
61.384
43.632
35.590
21.489
TsHN
NCS
O
O
Cl
3ea
ppm (t1)

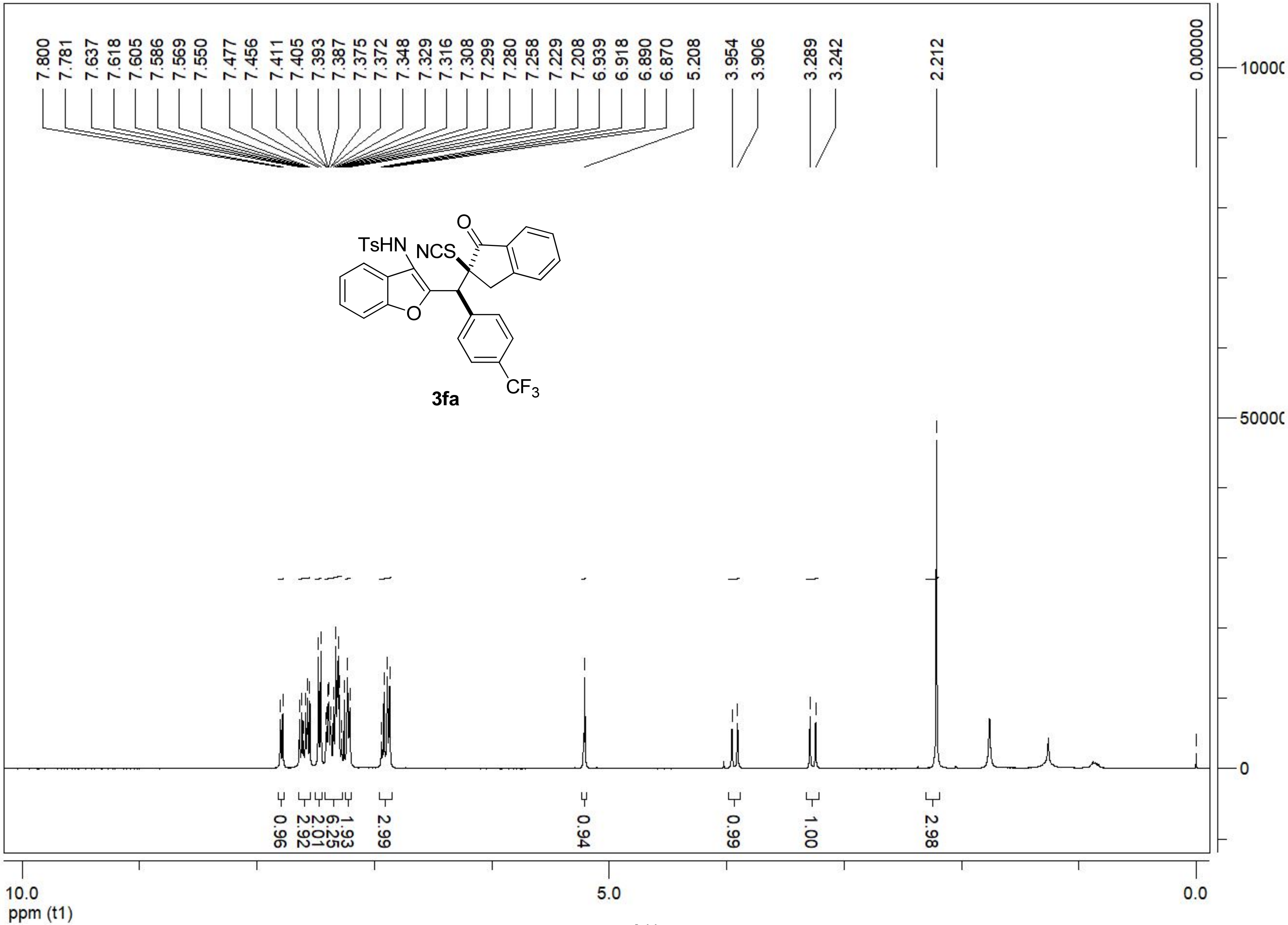
7.800 7.781 7.637 7.618 7.605 7.586 7.569 7.550 7.477 7.456 7.411 7.405 7.393 7.387 7.375 7.372 7.348 7.329 7.316 7.308 7.299 7.280 7.258 7.229 7.208 6.939 6.918 6.890 6.870 5.208
3.954 3.906
3.289 3.242
2.212
0.00000
TsHN
NCS
O
CF3
3fa
0.96 2.92 2.01 6.25 1.93 2.99
0.94
0.99
1.00
2.98
10000
50000
0
10.0
5.0
0.0
ppm (t1)

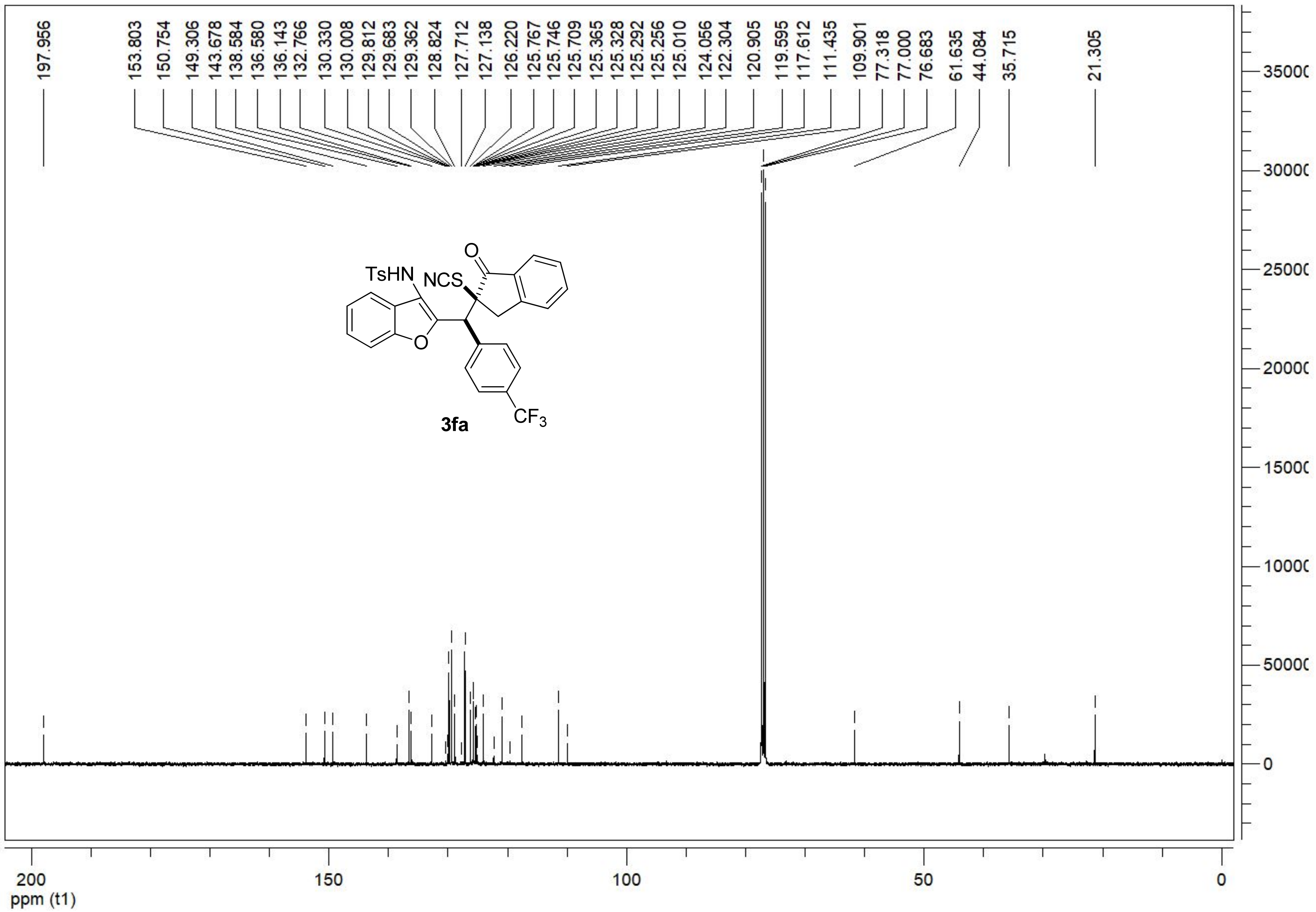
197.956
153.803
150.754
149.306
143.678
138.584
136.580
136.143
132.766
130.330
130.008
129.812
129.683
129.362
128.824
127.712
127.138
126.220
125.767
125.746
125.709
125.365
125.328
125.292
125.256
125.010
124.056
122.304
120.905
119.595
117.612
111.435
109.901
77.318
77.000
76.683
61.635
44.084
35.715
21.305
TsHN
NCS
O
CF3
3fa
35000
30000
25000
20000
15000
10000
50000
0
200
150
100
50
0
ppm (t1)

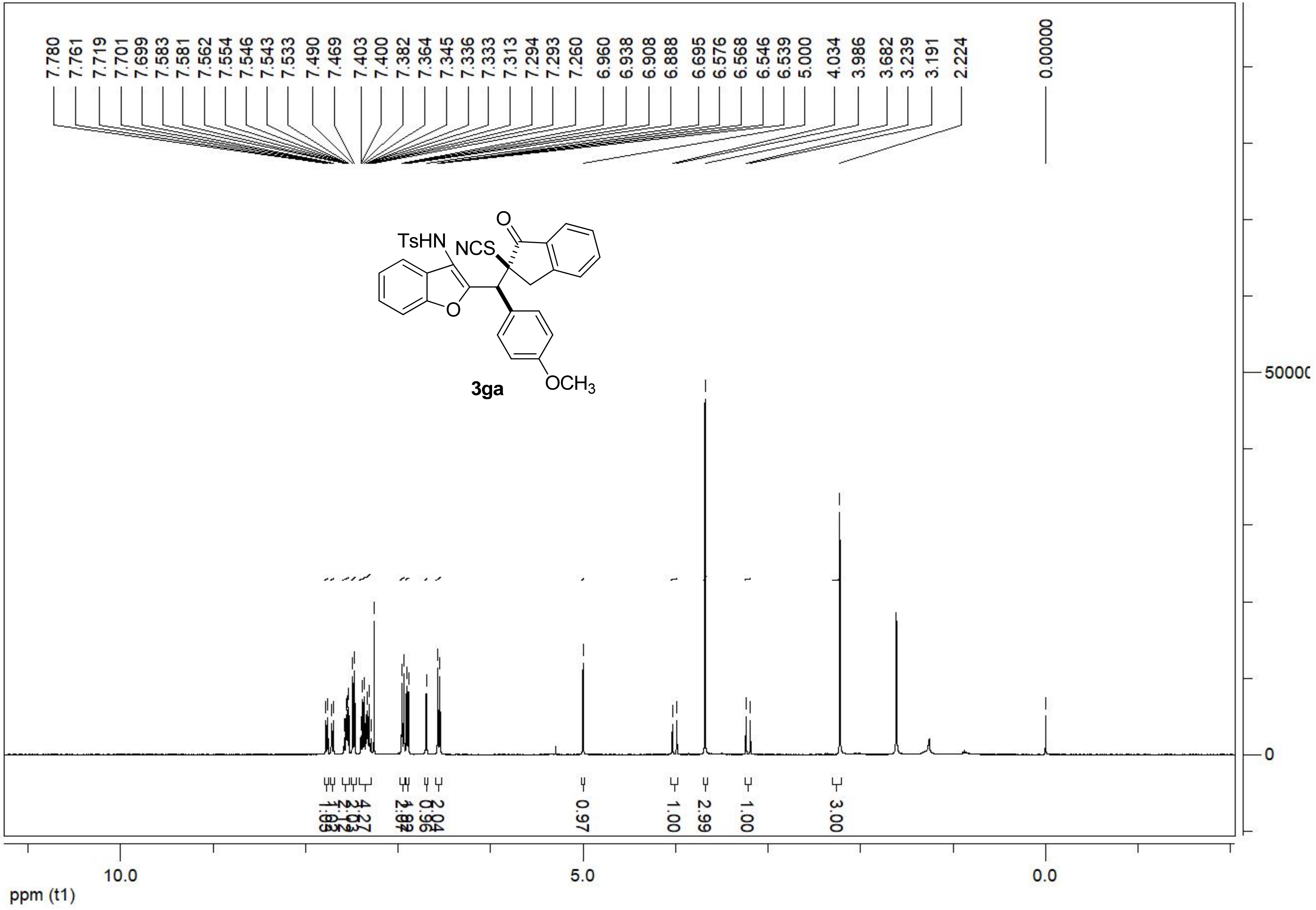
7.780
7.761
7.719
7.701
7.699
7.583
7.581
7.562
7.554
7.546
7.543
7.533
7.490
7.469
7.403
7.400
7.382
7.364
7.345
7.336
7.333
7.313
7.294
7.293
7.260
6.960
6.938
6.908
6.888
6.695
6.576
6.568
6.546
6.539
5.000
4.034
3.986
3.682
3.239
3.191
2.224
0.00000
TsHN
NCS
O
O
OCH3
3ga
50000
0
0.97
1.00
2.99
1.00
3.00
10.0
5.0
0.0
ppm (t1)

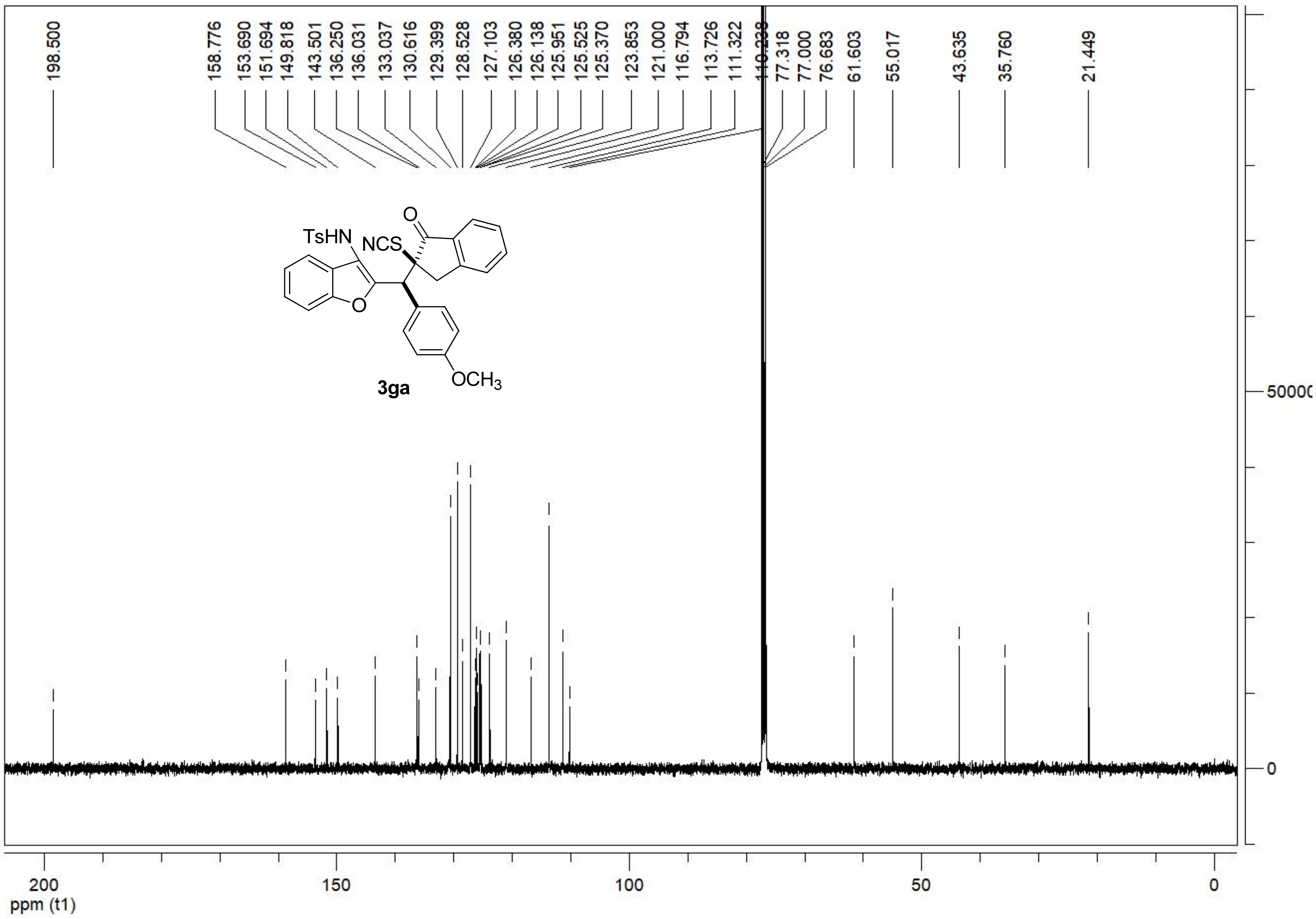
198.500
158.776
153.690
151.694
149.818
143.501
136.250
136.031
133.037
130.616
129.399
128.528
127.103
126.380
126.138
125.951
125.525
125.370
123.853
121.000
116.794
113.726
111.322
77.318
77.000
76.683
61.603
55.017
43.635
35.760
21.449
TsHN
NCS
O
O
OCH3
3ga
50000
0
200
150
100
50
0
ppm (t1)

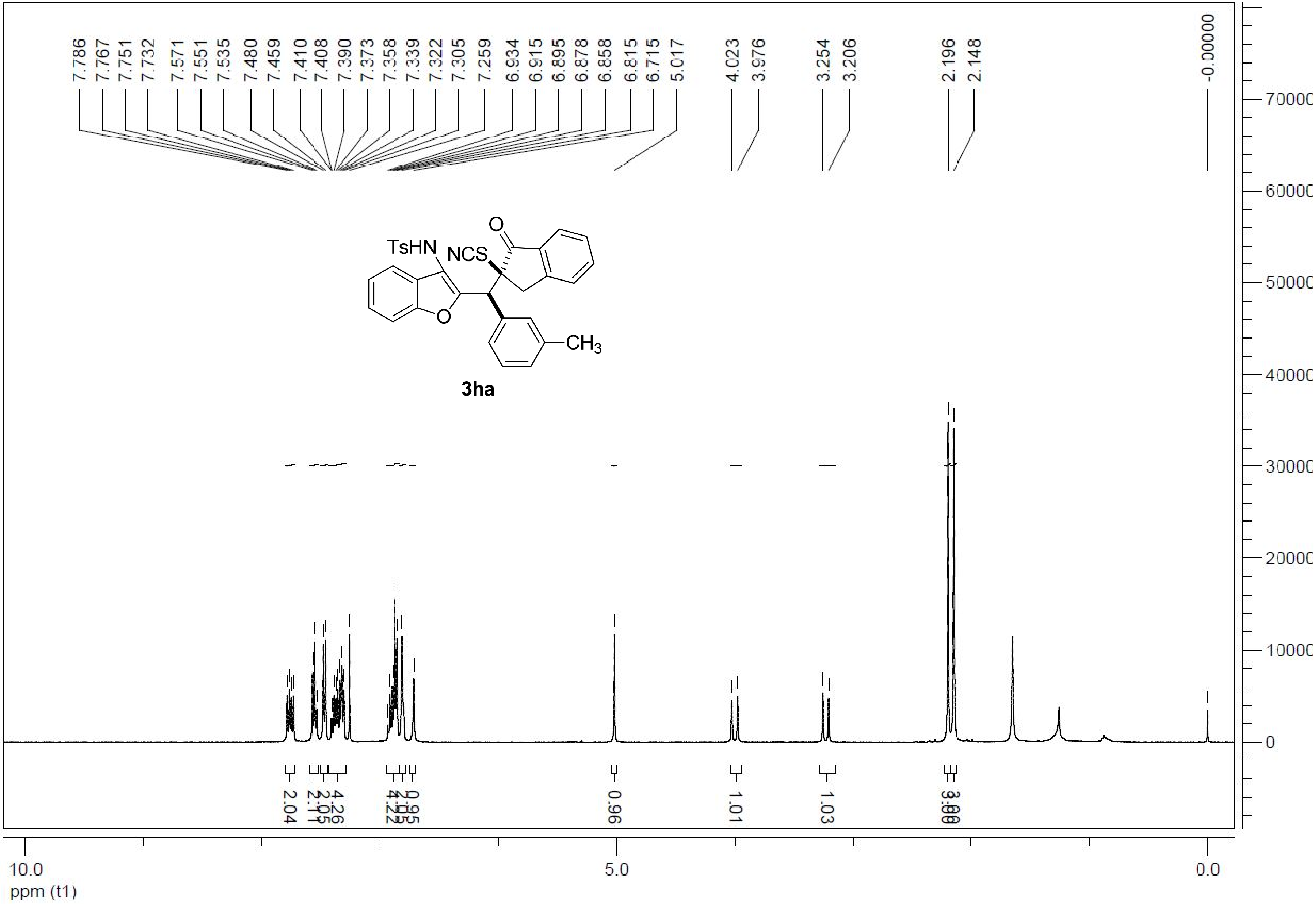
7.786
7.767
7.751
7.732
7.571
7.551
7.535
7.480
7.459
7.410
7.408
7.390
7.373
7.358
7.339
7.322
7.305
7.259
6.934
6.915
6.895
6.878
6.858
6.815
6.715
5.017
4.023
3.976
3.254
3.206
2.196
2.148
-0.00000
TsHN
NCS
O
CH3
3ha
2.04
0.96
1.01
1.03
10.0
5.0
0.0
ppm (t1)

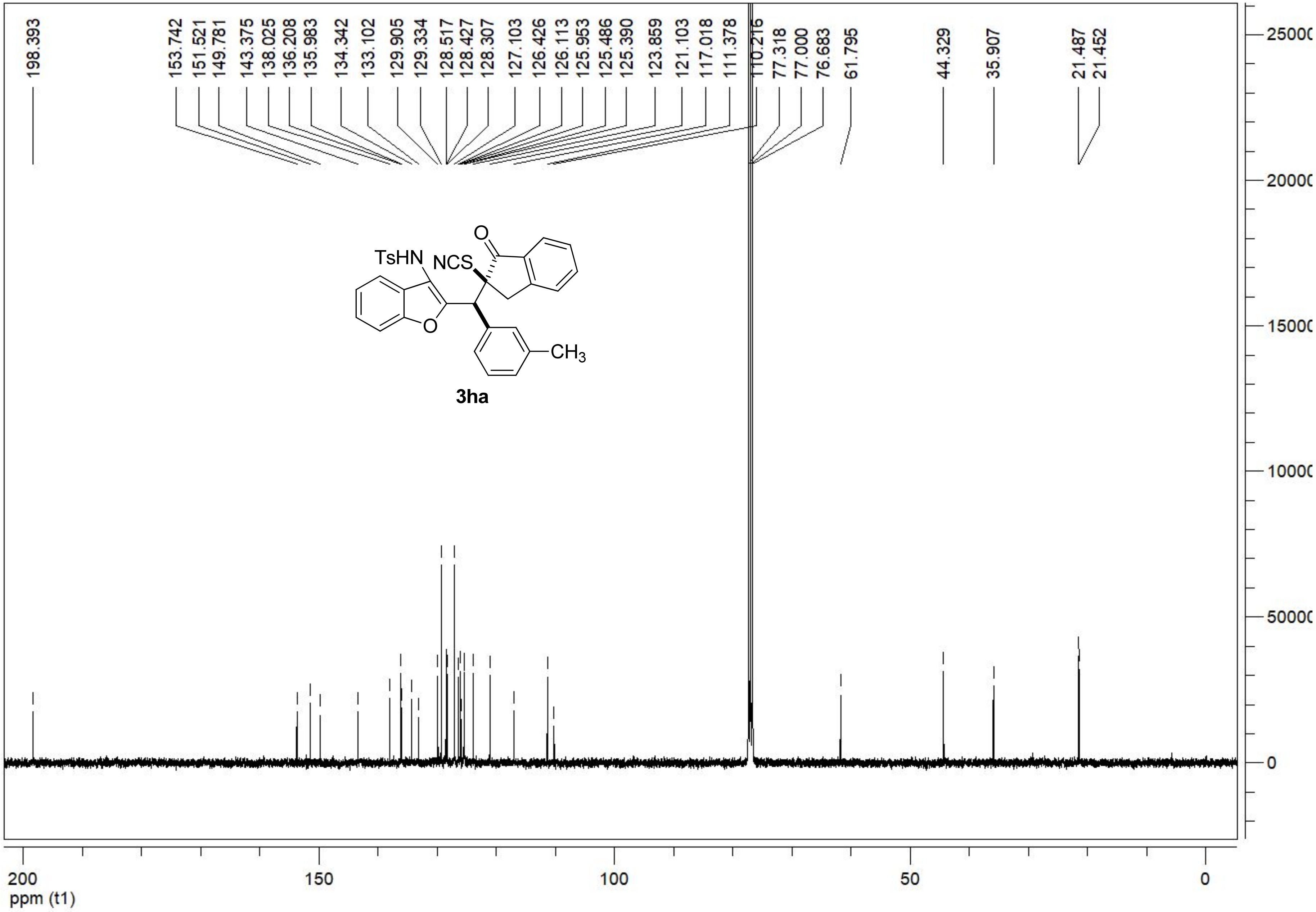
198.393
153.742
151.521
149.781
143.375
138.025
136.208
135.983
134.342
133.102
129.905
129.334
128.517
128.427
128.307
127.103
126.426
126.113
125.953
125.486
125.390
123.859
121.103
117.018
111.378
110.216
77.318
77.000
76.683
61.795
44.329
35.907
21.487
21.452
TsHN
NCS
O
O
CH3
3ha
25000
20000
15000
10000
50000
0
200
150
100
50
0
ppm (t1)

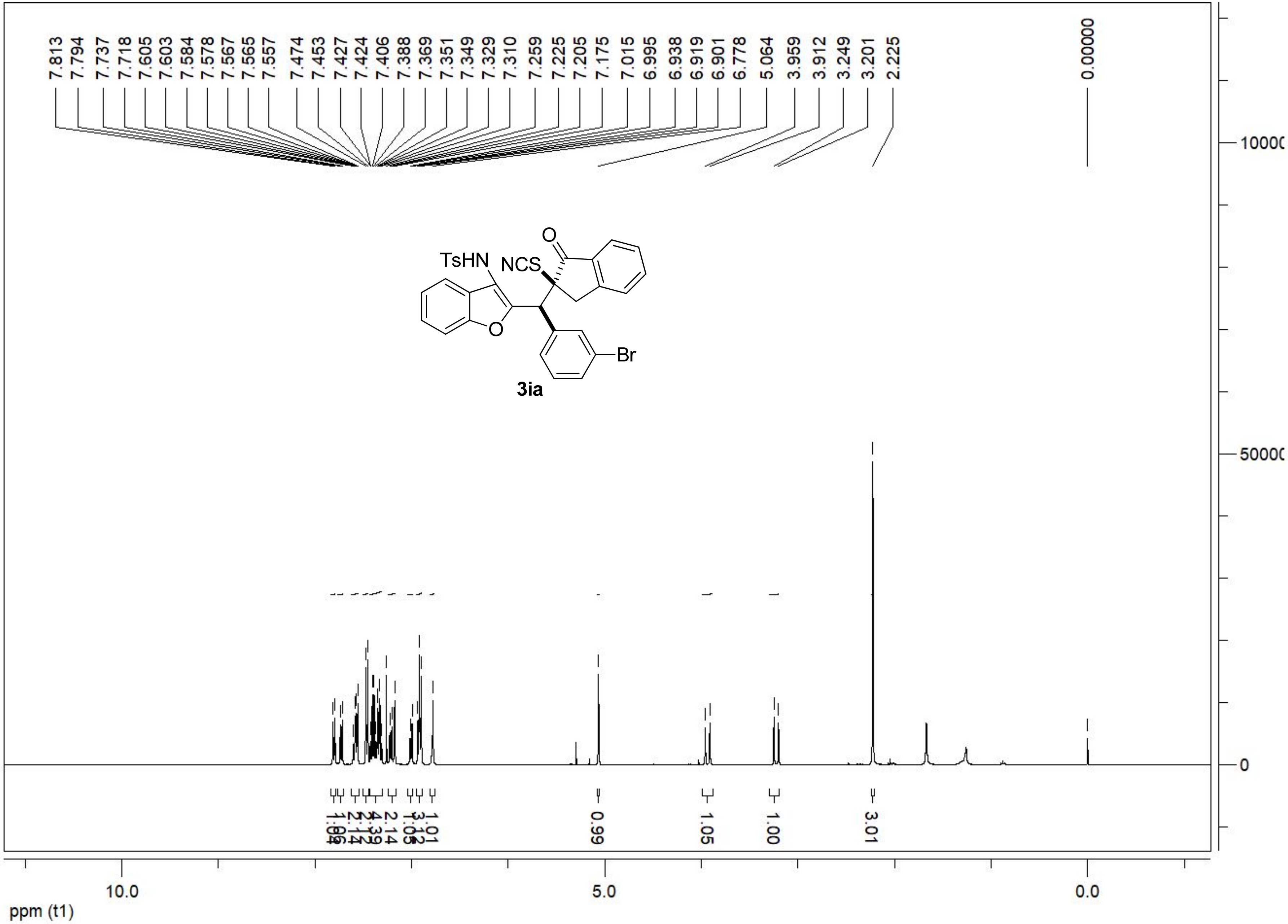
7.813 7.794 7.737 7.718 7.605 7.603 7.584 7.578 7.567 7.565 7.557 7.474 7.453 7.427 7.424 7.406 7.388 7.369 7.351 7.349 7.329 7.310 7.259 7.225 7.205 7.175 7.015 6.995 6.938 6.919 6.901 6.778 5.064 3.959 3.912 3.249 3.201 2.225
0.00000
TsHN
NCS
O
O
Br
3ia
2.14
1.01
0.99
1.05
1.00
3.01
10.0
5.0
0.0
ppm (t1)

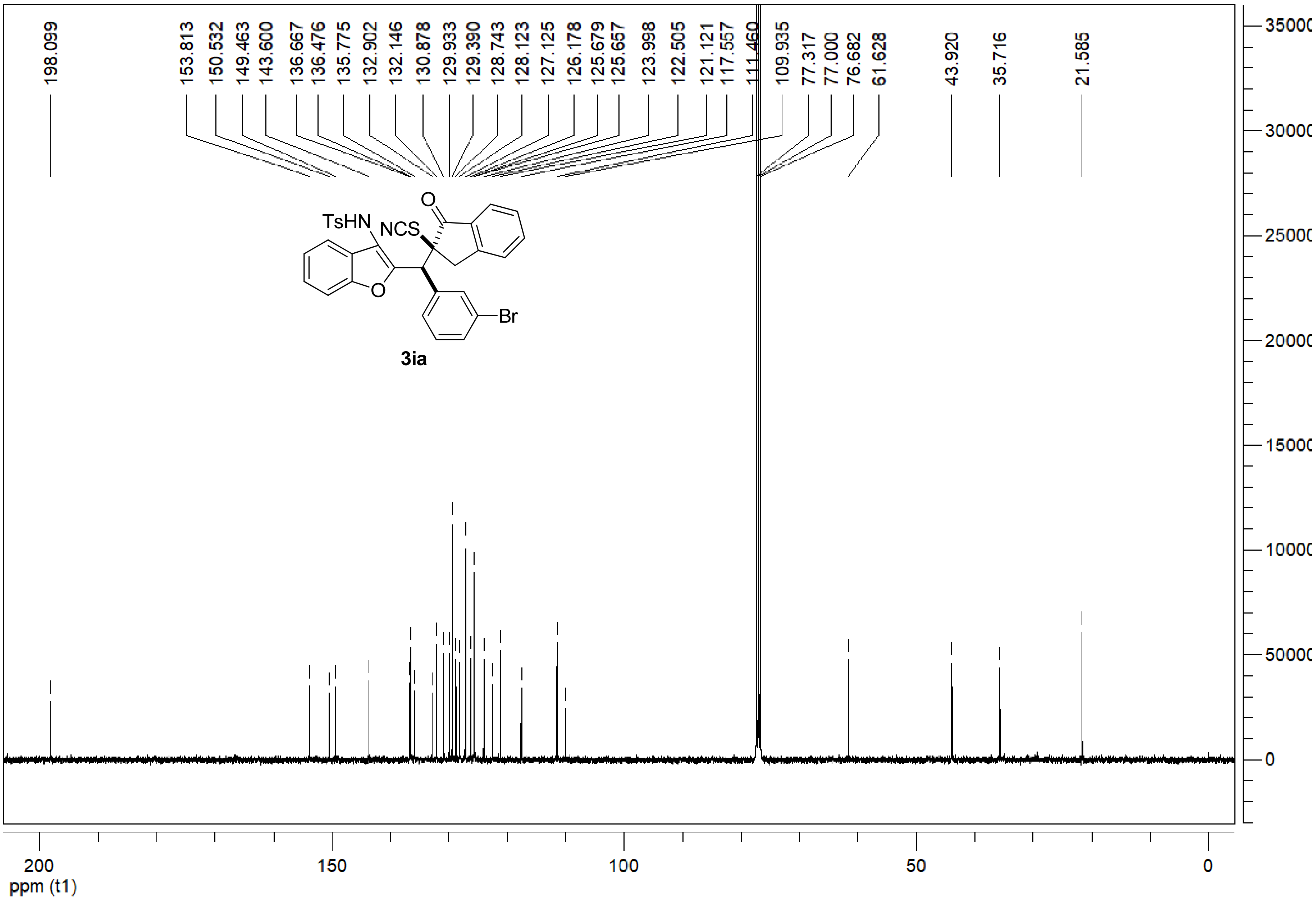
198.099
153.813
150.532
149.463
143.600
136.667
136.476
135.775
132.902
132.146
130.878
129.933
129.390
128.743
128.123
127.125
126.178
125.679
125.657
123.998
122.505
121.121
117.557
111.460
109.935
77.317
77.000
76.682
61.628
43.920
35.716
21.585
TsHN
NCS
O
Br
3ia
200
150
100
50
0
ppm (t1)

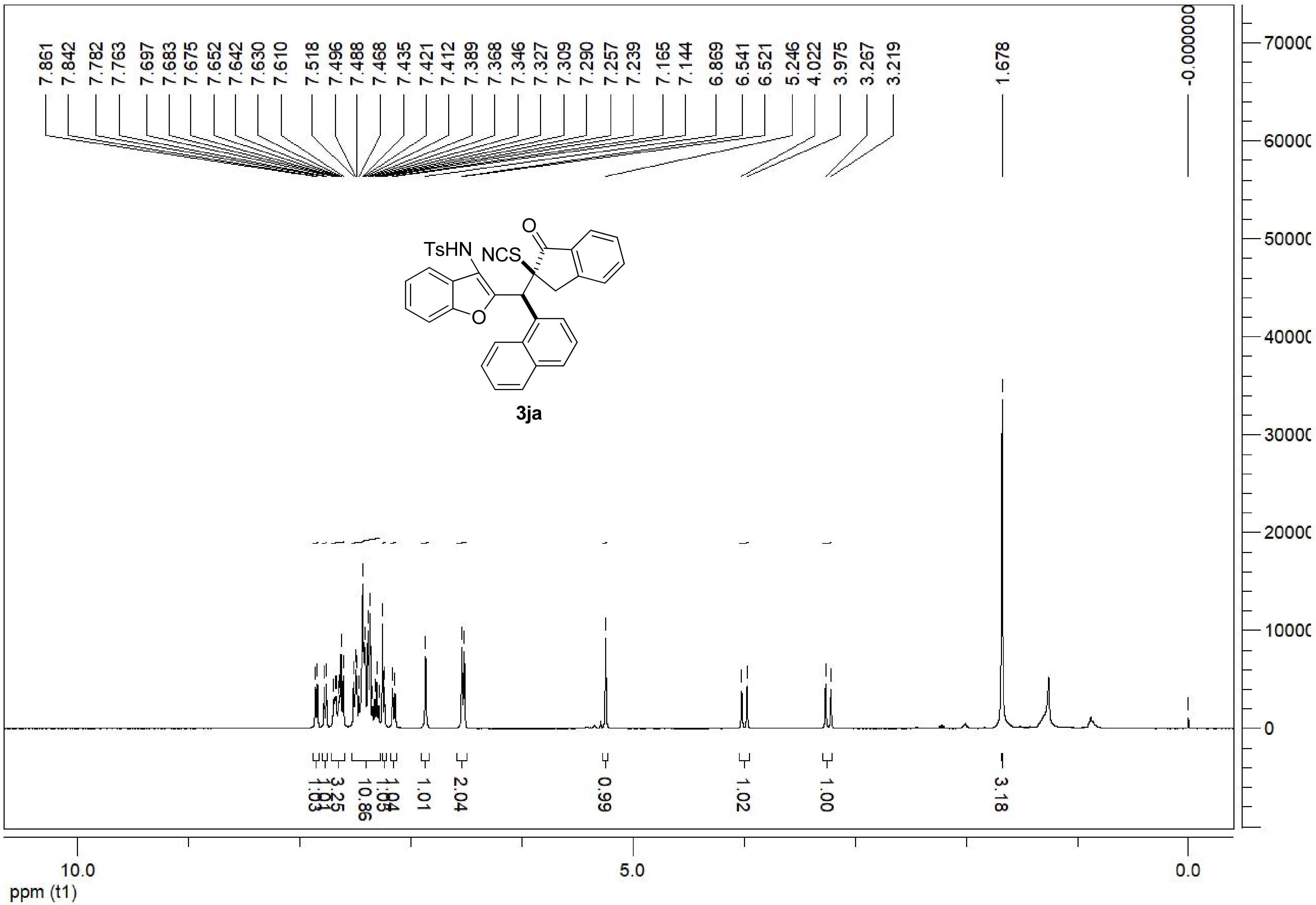
7.861
7.842
7.782
7.763
7.697
7.683
7.675
7.652
7.642
7.630
7.610
7.518
7.496
7.488
7.468
7.435
7.421
7.412
7.389
7.368
7.346
7.327
7.309
7.290
7.257
7.239
7.165
7.144
6.869
6.541
6.521
5.246
4.022
3.975
3.267
3.219
1.678
-0.000000
TsHN
NCS
O
O
3ja
1.01
2.04
0.99
1.02
1.00
3.18
10.86
3.25
10.0
5.0
0.0
ppm (t1)
70000
60000
50000
40000
30000
20000
10000
0

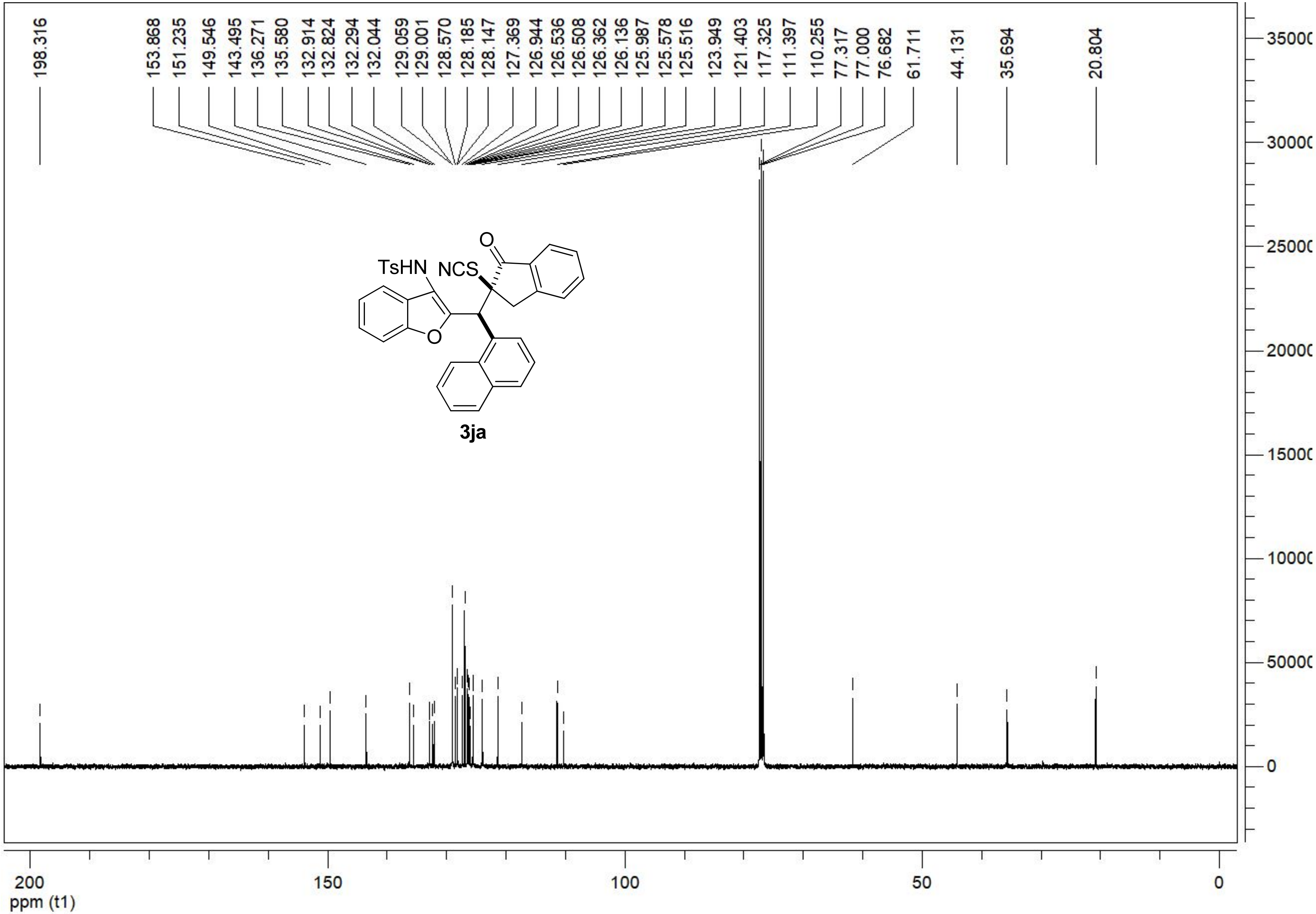
198.316
153.868
151.235
149.546
143.495
136.271
135.580
132.914
132.824
132.294
132.044
129.059
129.001
128.570
128.185
128.147
127.369
126.944
126.536
126.508
126.362
126.136
125.987
125.578
125.516
123.949
121.403
117.325
111.397
110.255
77.317
77.000
76.682
61.711
44.131
35.694
20.804
TsHN
NCS
O
O
3ja
35000
30000
25000
20000
15000
10000
50000
0
200
150
100
50
0
ppm (t1)

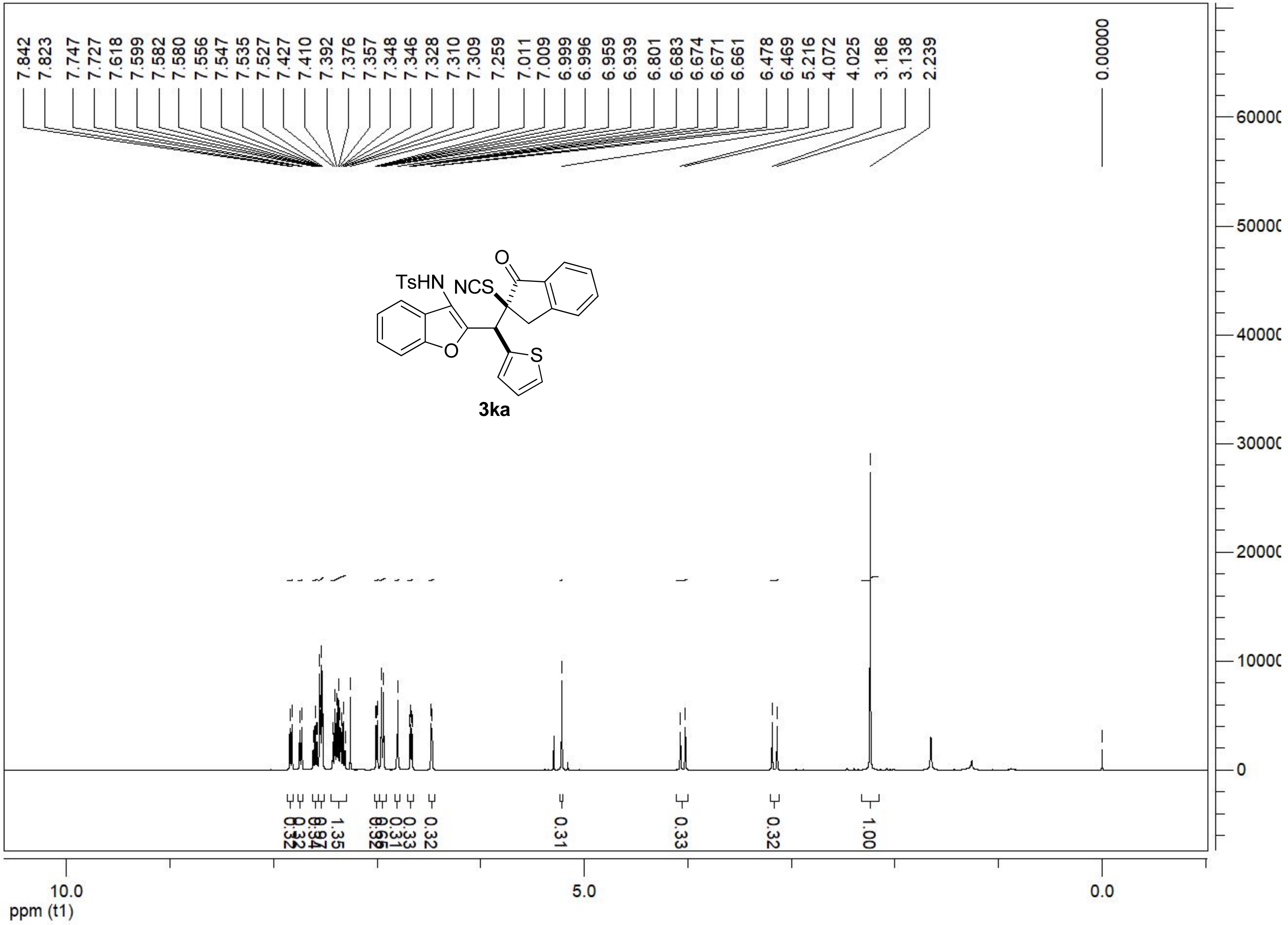
7.842
7.823
7.747
7.727
7.618
7.599
7.582
7.580
7.556
7.547
7.535
7.527
7.427
7.410
7.392
7.376
7.357
7.348
7.346
7.328
7.310
7.309
7.259
7.011
7.009
6.999
6.996
6.959
6.939
6.801
6.683
6.674
6.671
6.661
6.478
6.469
5.216
4.072
4.025
3.186
3.138
2.239
0.00000
TsHN
NCS
O
O
S
3ka
1.35
0.31
0.33
0.32
0.31
0.33
0.32
1.00
60000
50000
40000
30000
20000
10000
0
10.0
5.0
0.0
ppm (t1)

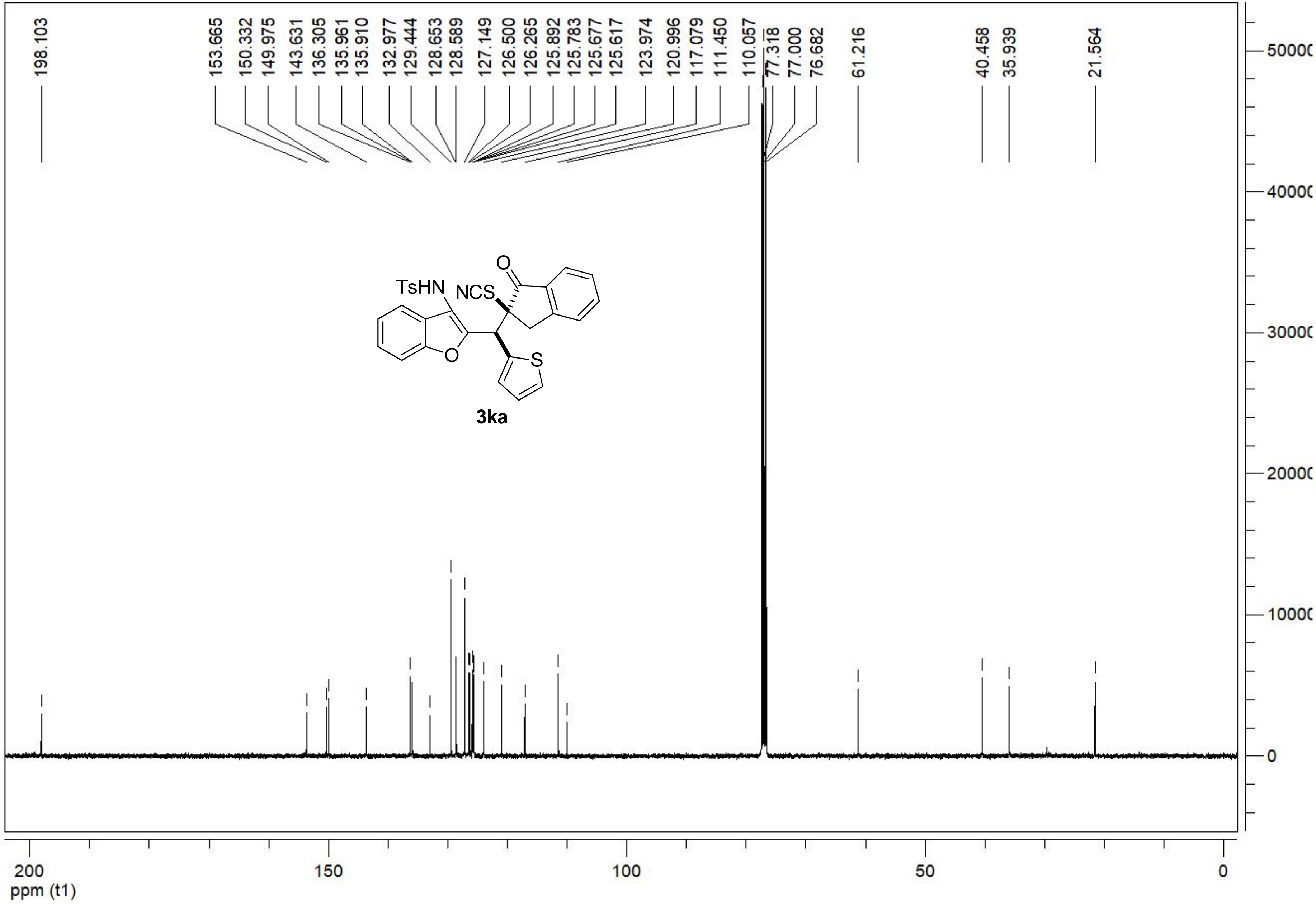
198.103
153.665
150.332
149.975
143.631
136.305
135.961
135.910
132.977
129.444
128.653
128.589
127.149
126.500
126.265
125.892
125.783
125.677
125.617
123.974
120.996
117.079
111.450
110.057
77.318
77.000
76.682
61.216
40.458
35.939
21.564
TsHN
NCS
O
S
3ka
50000
40000
30000
20000
10000
0
200
150
100
50
0
ppm (t1)

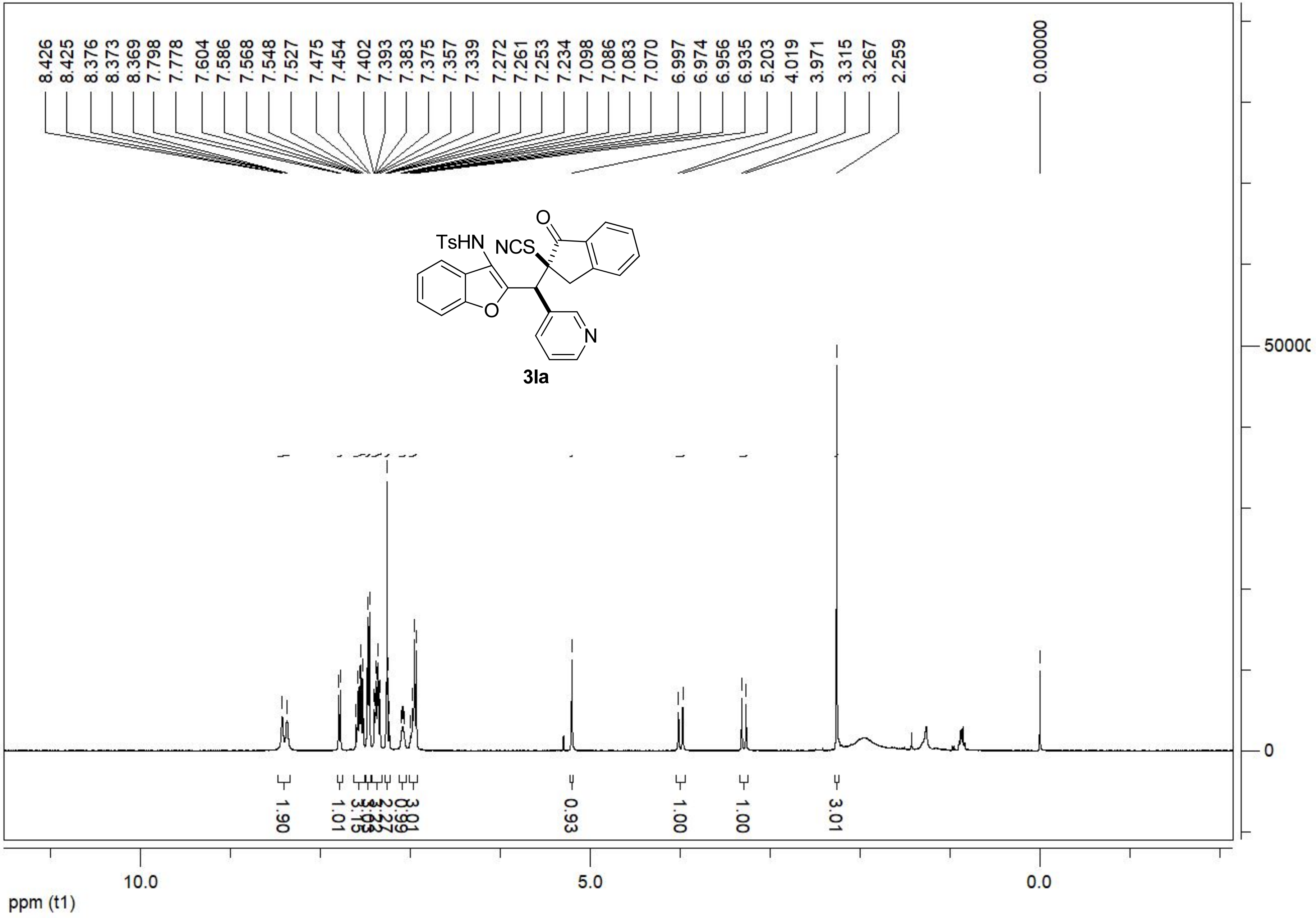
8.426 8.425 8.376 8.373 8.369 7.798 7.778 7.604 7.586 7.568 7.548 7.527 7.475 7.454 7.402 7.393 7.383 7.375 7.357 7.339 7.272 7.261 7.253 7.234 7.098 7.086 7.083 7.070 6.997 6.974 6.956 6.935 5.203 4.019 3.971 3.315 3.267 2.259
0.00000
TsHN
NCS
O
N
3la
50000
0
1.90 1.01 3.15 1.03 4.22 2.27 0.99 3.01 0.93 1.00 1.00 3.01
10.0
5.0
0.0
ppm (t1)

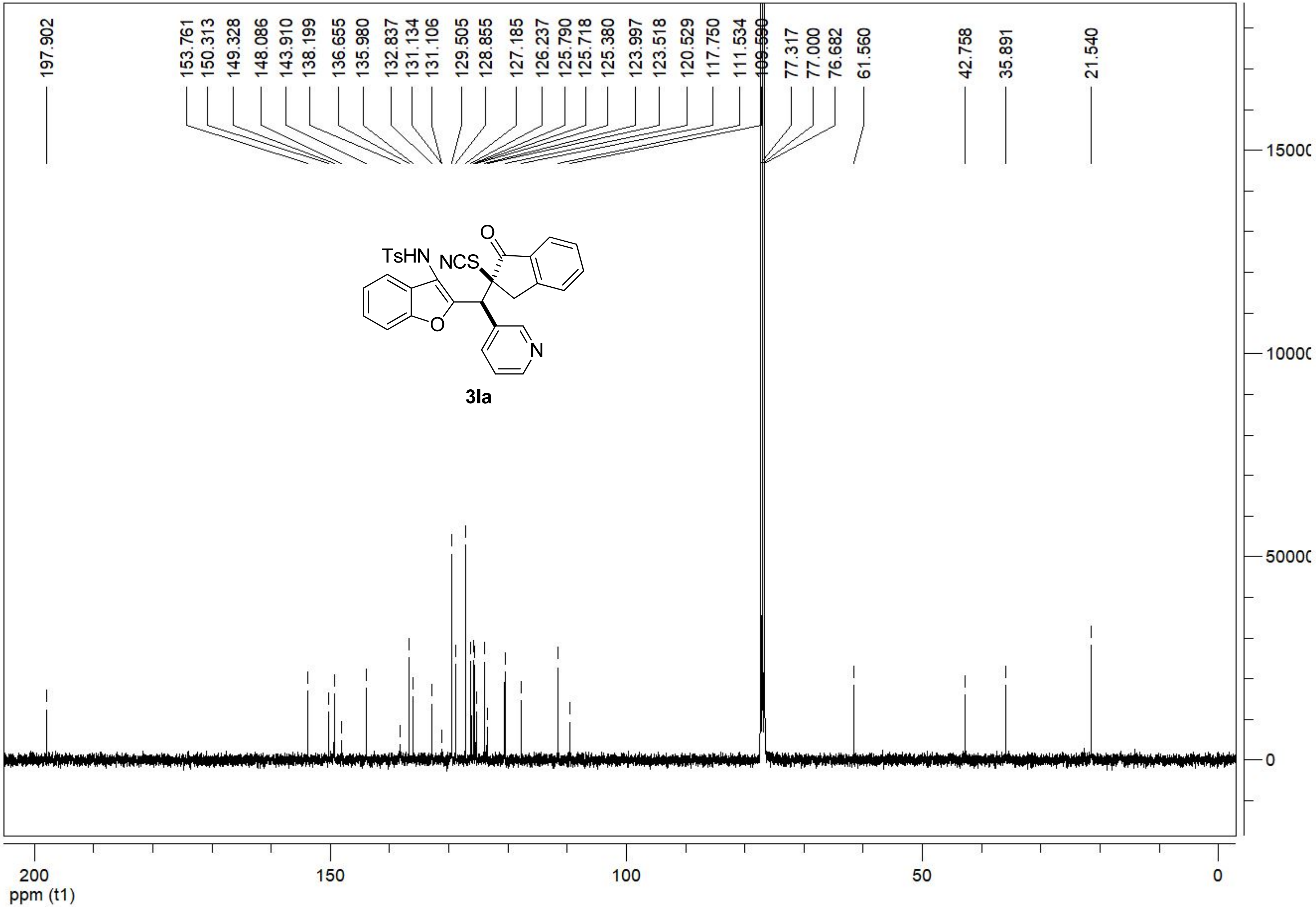
197.902
153.761
150.313
149.328
148.086
143.910
138.199
136.655
135.980
132.837
131.134
131.106
129.505
128.855
127.185
126.237
125.790
125.718
125.380
123.997
123.518
120.529
117.750
111.534
109.590
77.317
77.000
76.682
61.560
42.758
35.891
21.540
TsHN
NCS
O
O
N
3la
150000
100000
50000
0
200
150
100
50
0
ppm (t1)

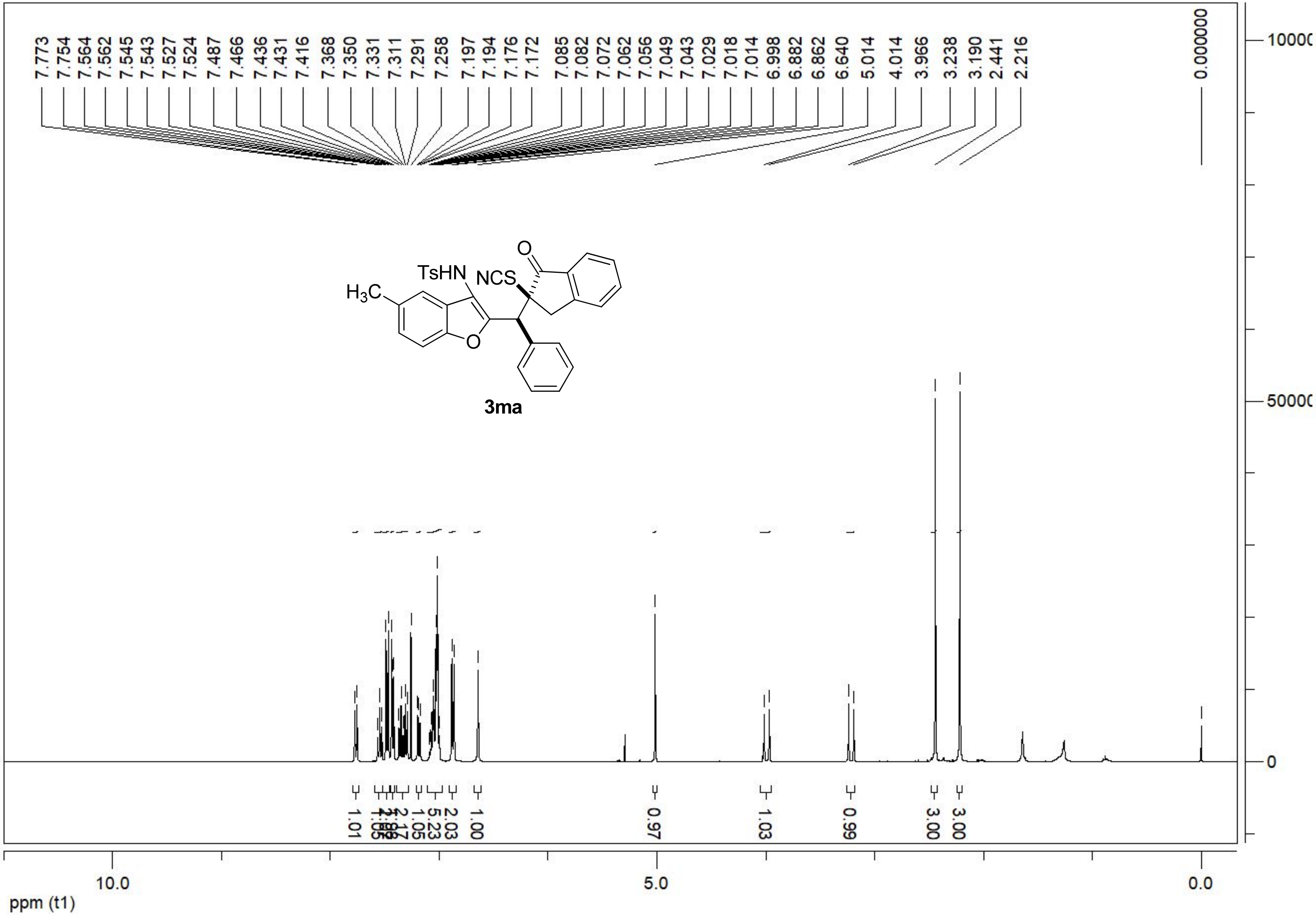
7.773
7.754
7.564
7.562
7.545
7.543
7.527
7.524
7.487
7.466
7.436
7.431
7.416
7.368
7.350
7.331
7.311
7.291
7.258
7.197
7.194
7.176
7.172
7.085
7.082
7.072
7.062
7.056
7.049
7.043
7.029
7.018
7.014
6.998
6.882
6.862
6.640
5.014
4.014
3.966
3.238
3.190
2.441
2.216
0.000000
TsHN
NCS
O
H3C
O
3ma
1.01
1.00
0.97
1.03
0.99
3.00
3.00
2.17
1.05
5.23
2.03
10.0
5.0
0.0
ppm (t1)
0
50000
10000

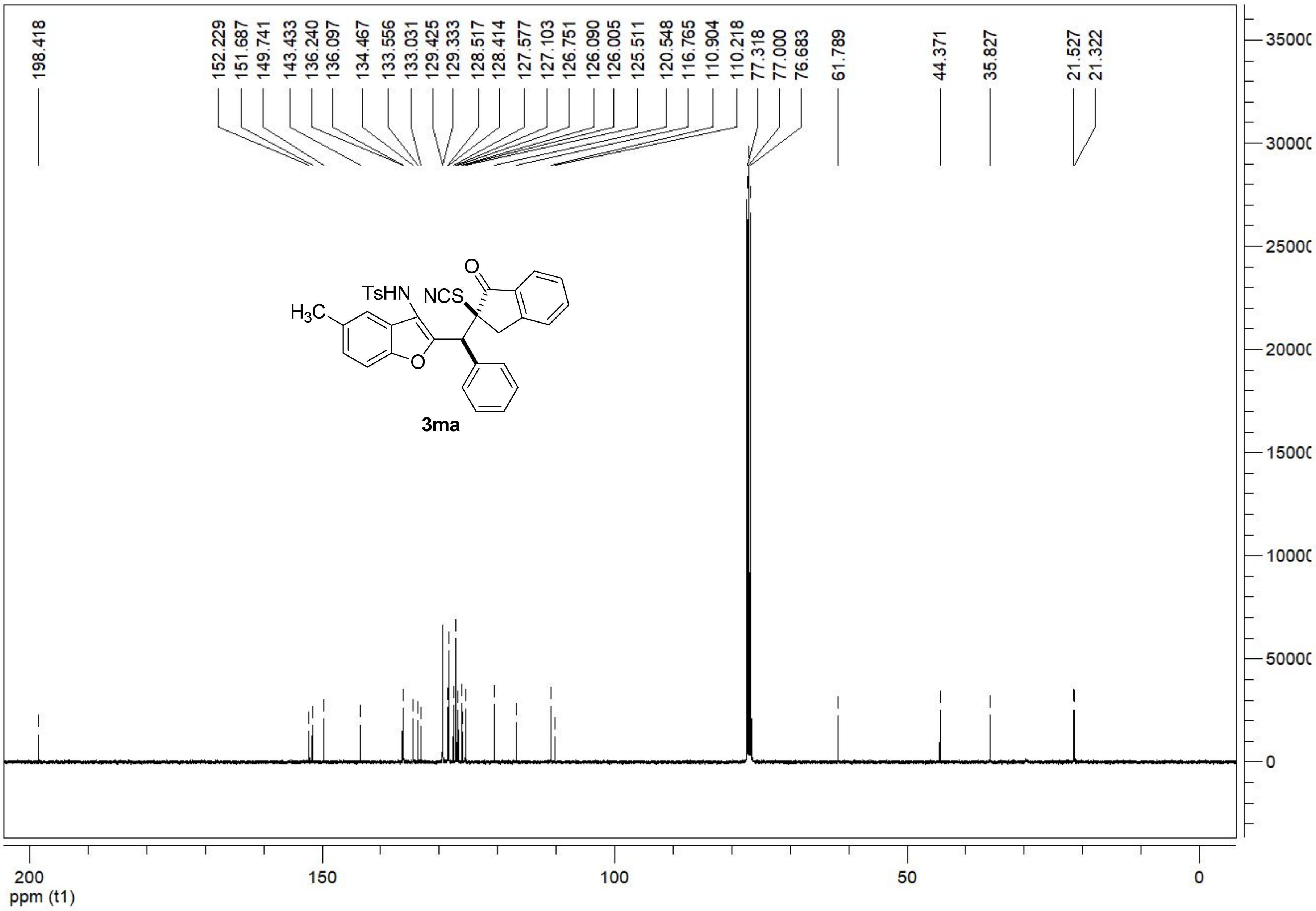
198.418
152.229
151.687
149.741
143.433
136.240
136.097
134.467
133.556
133.031
129.425
129.333
128.517
128.414
127.577
127.103
126.751
126.090
126.005
125.511
120.548
116.765
110.904
110.218
77.318
77.000
76.683
61.789
44.371
35.827
21.527
21.322
TsHN
NCS
O
H3C
O
3ma
200
150
100
50
0
ppm (t1)

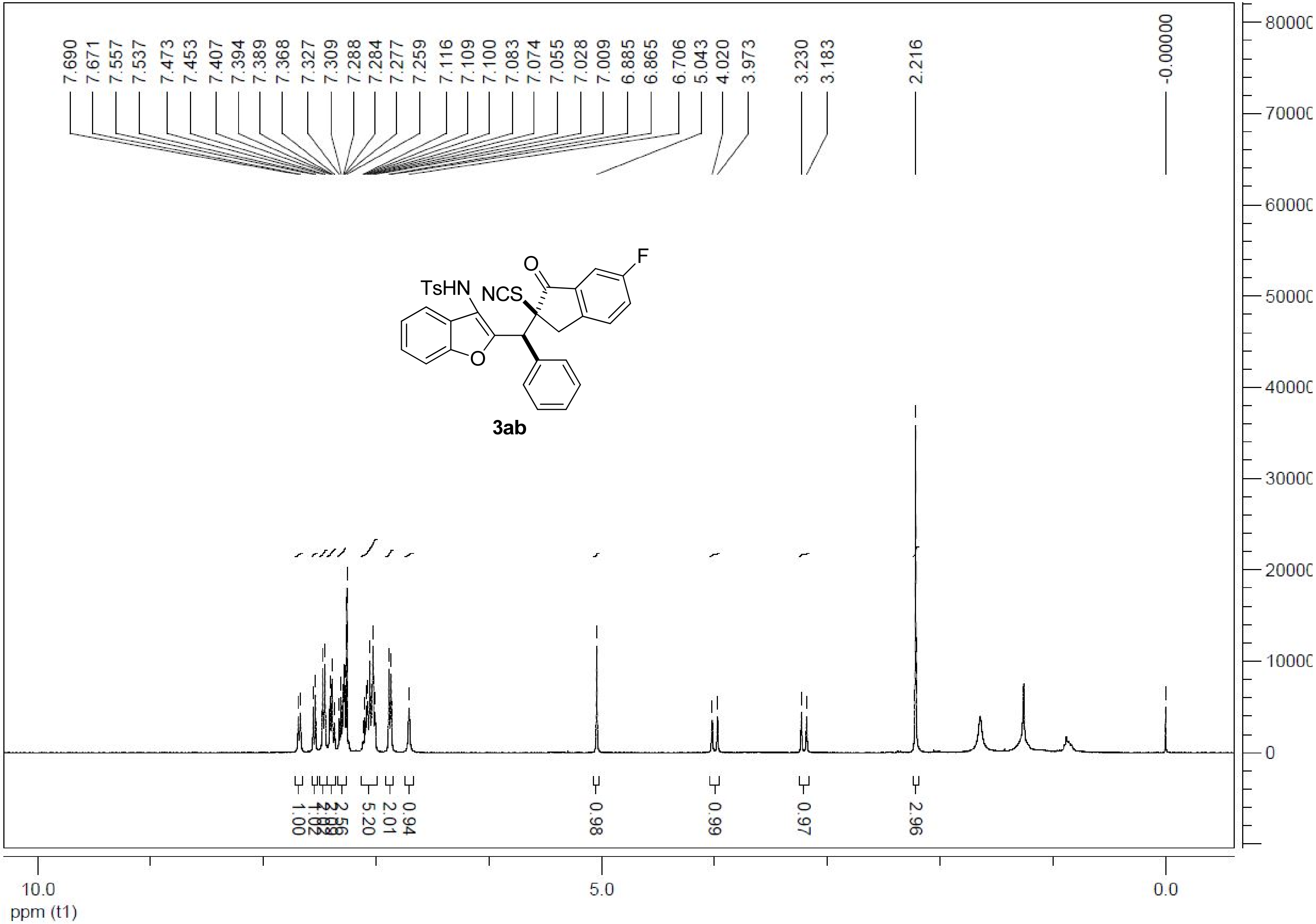
7.690
7.671
7.557
7.537
7.473
7.453
7.407
7.394
7.389
7.368
7.327
7.309
7.288
7.284
7.277
7.259
7.116
7.109
7.100
7.083
7.074
7.055
7.028
7.009
6.885
6.865
6.706
5.043
4.020
3.973
3.230
3.183
2.216
-0.00000
TsHN
NCS
O
F
3ab
1.00
2.56
5.20
2.01
0.94
0.98
0.99
0.97
2.96
10.0
5.0
0.0
ppm (t1)
80000
70000
60000
50000
40000
30000
20000
10000
0

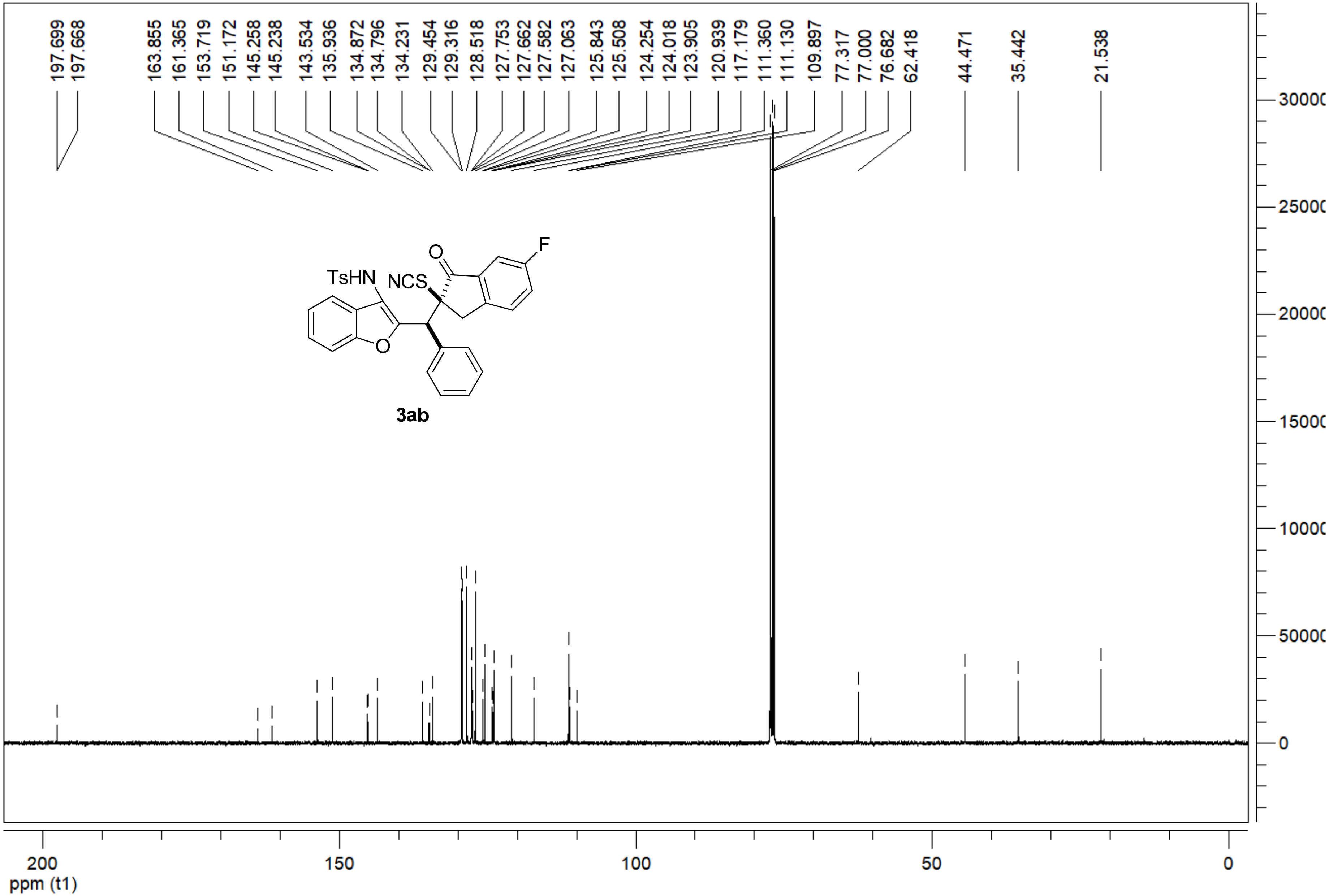
197.699
197.668
163.855
161.365
153.719
151.172
145.258
145.238
143.534
135.936
134.872
134.796
134.231
129.454
129.316
128.518
127.753
127.662
127.582
127.063
125.843
125.508
124.254
124.018
123.905
120.939
117.179
111.360
111.130
109.897
77.317
77.000
76.682
62.418
44.471
35.442
21.538
TsHN
NCS
O
F
O
3ab
200
150
100
50
0
ppm (t1)
0
50000
10000
15000
20000
25000
30000

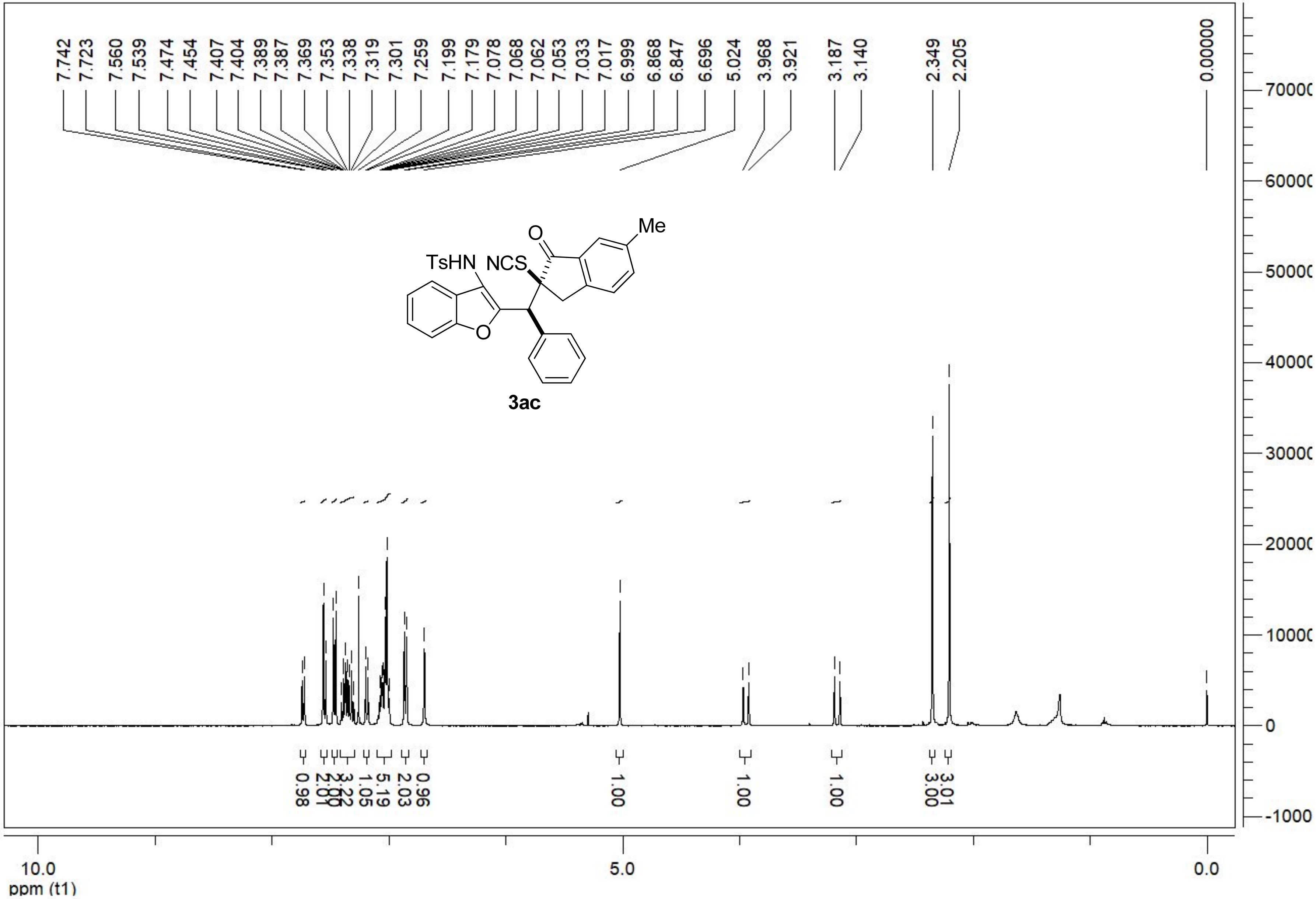
7.742
7.723
7.560
7.539
7.474
7.454
7.407
7.404
7.389
7.387
7.369
7.353
7.338
7.319
7.301
7.259
7.199
7.179
7.078
7.068
7.062
7.053
7.033
7.017
6.999
6.868
6.847
6.696
5.024
3.968
3.921
3.187
3.140
2.349
2.205
0.00000
TsHN
NCS
O
Me
O
3ac
0.98
2.01
2.00
3.22
1.05
5.19
2.03
0.96
1.00
1.00
1.00
3.00
3.01
10.0
ppm (t1)
5.0
0.0

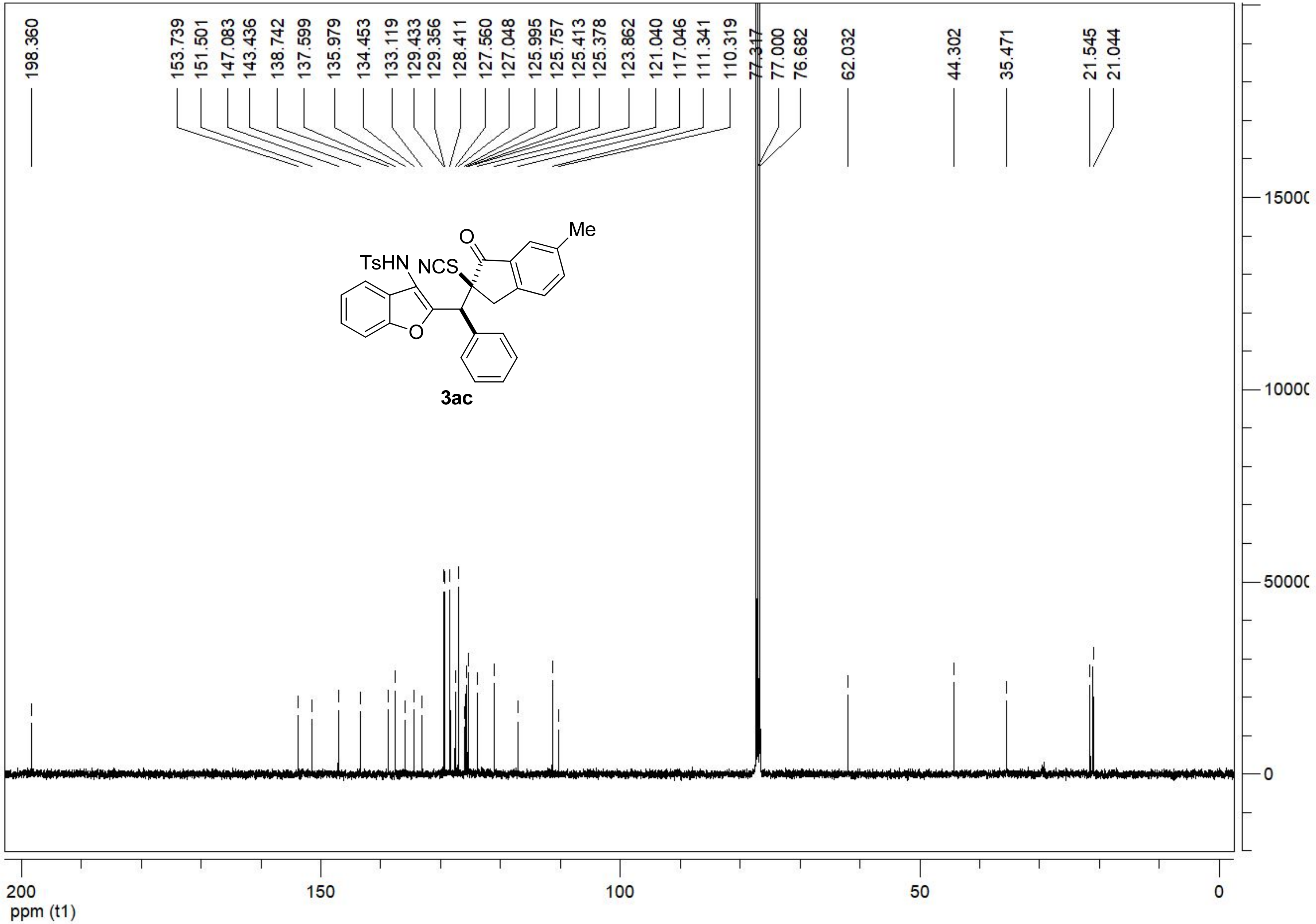
198.360
153.739
151.501
147.083
143.436
138.742
137.599
135.979
134.453
133.119
129.433
129.356
128.411
127.560
127.048
125.995
125.757
125.413
125.378
123.862
121.040
117.046
111.341
110.319
77.317
77.000
76.682
62.032
44.302
35.471
21.545
21.044
TsHN
NCS
O
Me
O
3ac
150000
100000
50000
0
200
150
100
50
0
ppm (t1)

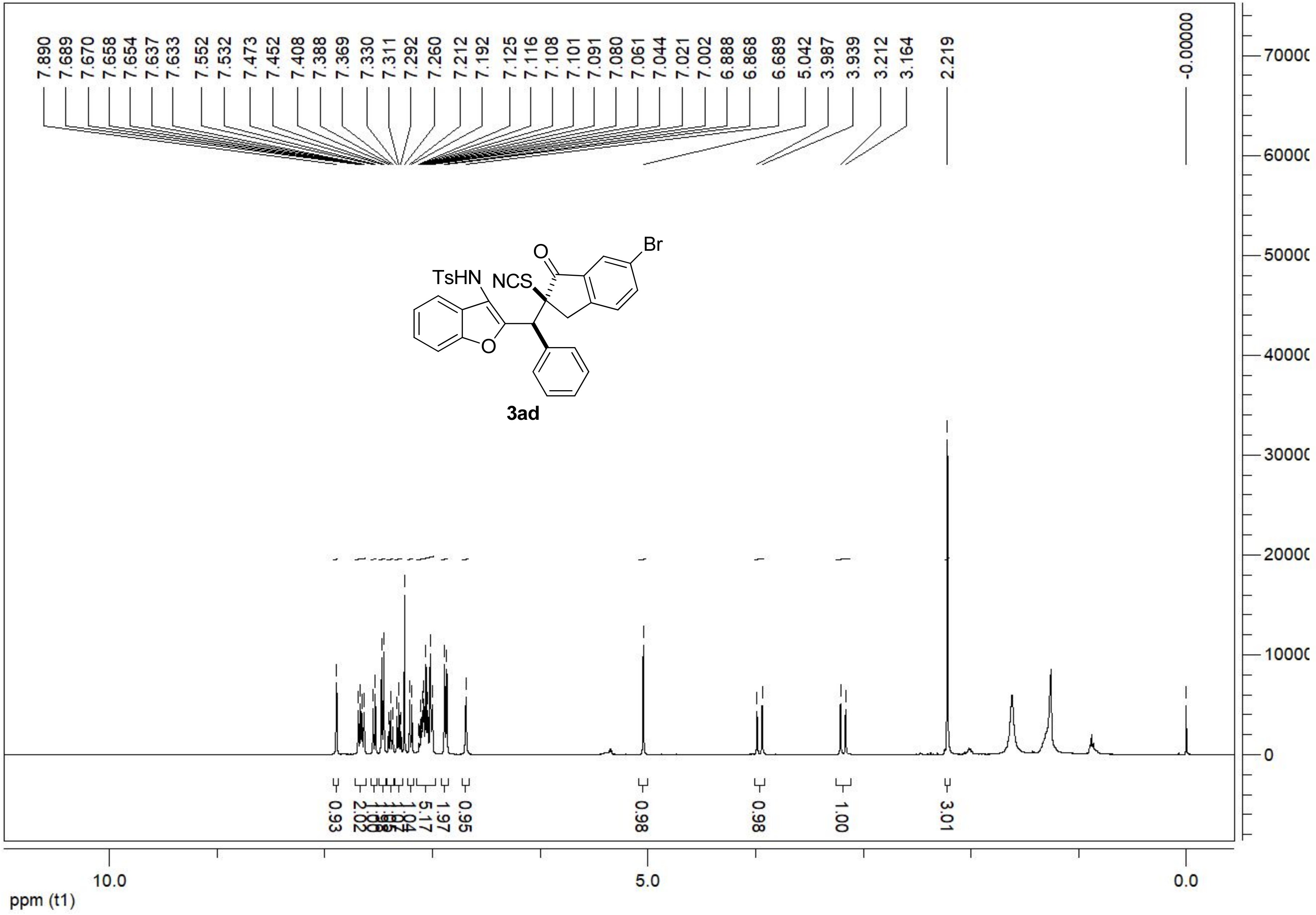
7.890
7.689
7.670
7.658
7.654
7.637
7.633
7.552
7.532
7.473
7.452
7.408
7.388
7.369
7.330
7.311
7.292
7.260
7.212
7.192
7.125
7.116
7.108
7.101
7.091
7.080
7.061
7.044
7.021
7.002
6.888
6.868
6.689
5.042
3.987
3.939
3.212
3.164
2.219
-0.00000
TsHN
NCS
O
Br
3ad
0.93
2.02
1.04
5.17
1.97
0.95
0.98
0.98
1.00
3.01
70000
60000
50000
40000
30000
20000
10000
0
10.0
5.0
0.0
ppm (t1)

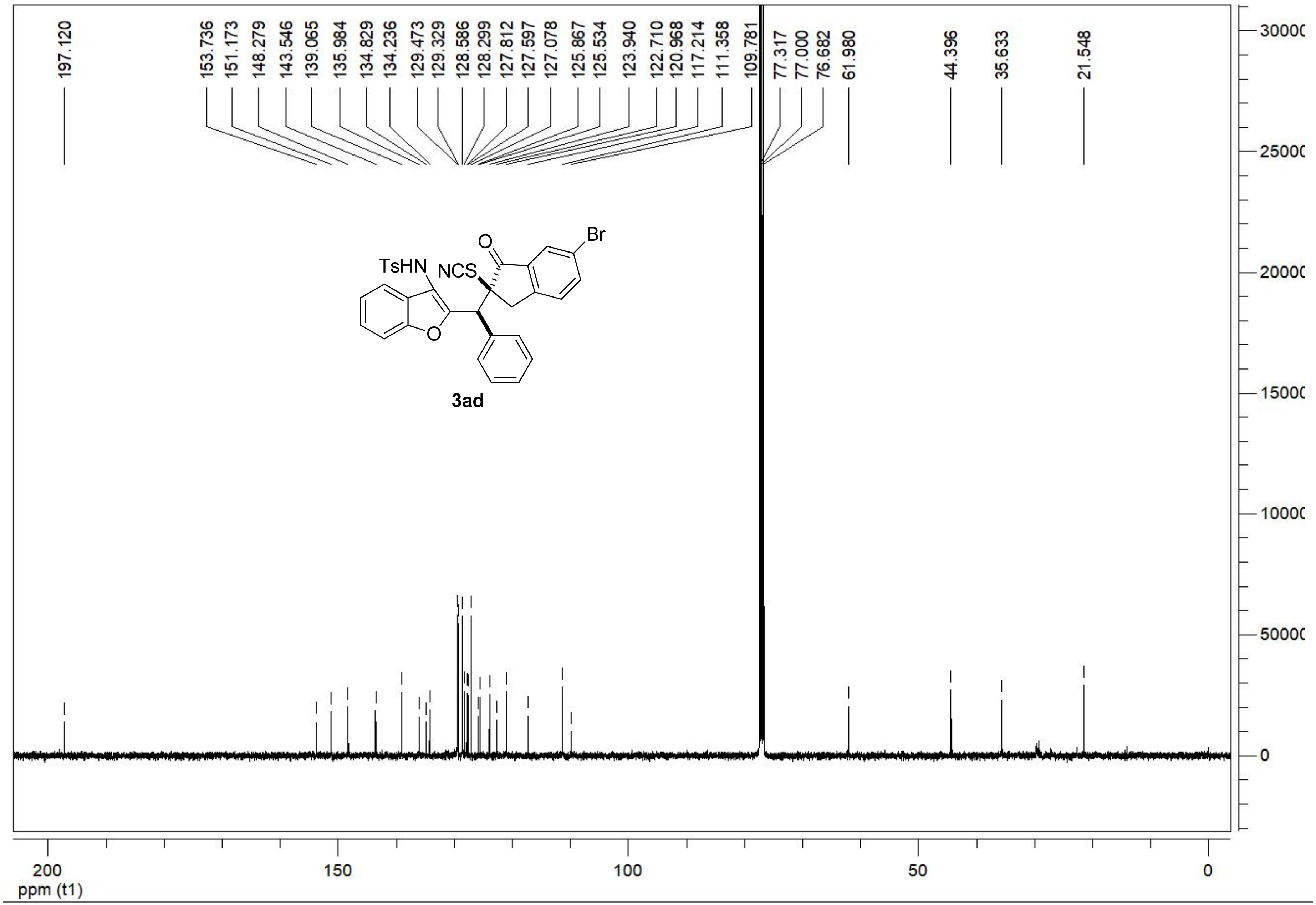
197.120
153.736
151.173
148.279
143.546
139.065
135.984
134.829
134.236
129.473
129.329
128.586
128.299
127.812
127.597
127.078
125.867
125.534
123.940
122.710
120.968
117.214
111.358
109.781
77.317
77.000
76.682
61.980
44.396
35.633
21.548
TsHN
NCS
O
Br
O
3ad
ppm (t1)
200
150
100
50
0

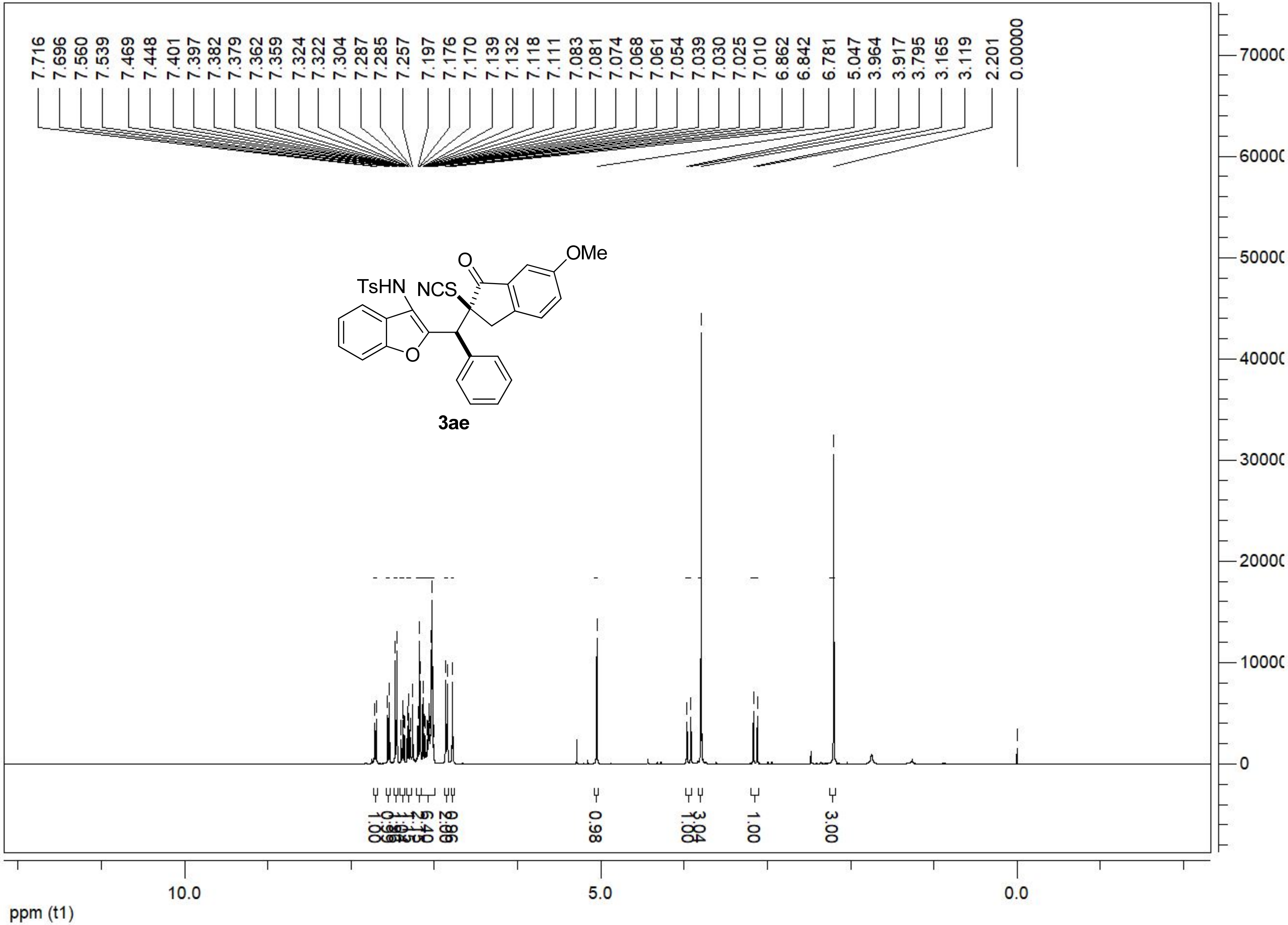
7.716
7.696
7.560
7.539
7.469
7.448
7.401
7.397
7.382
7.379
7.362
7.359
7.324
7.322
7.304
7.287
7.285
7.257
7.197
7.176
7.170
7.139
7.132
7.118
7.111
7.083
7.081
7.074
7.068
7.061
7.054
7.039
7.030
7.025
7.010
6.862
6.842
6.781
5.047
3.964
3.917
3.795
3.165
3.119
2.201
0.00000
TsHN
NCS
O
OMe
3ae
0.98
1.00
3.04
1.00
3.00
70000
60000
50000
40000
30000
20000
10000
0
10.0
5.0
0.0
ppm (t1)

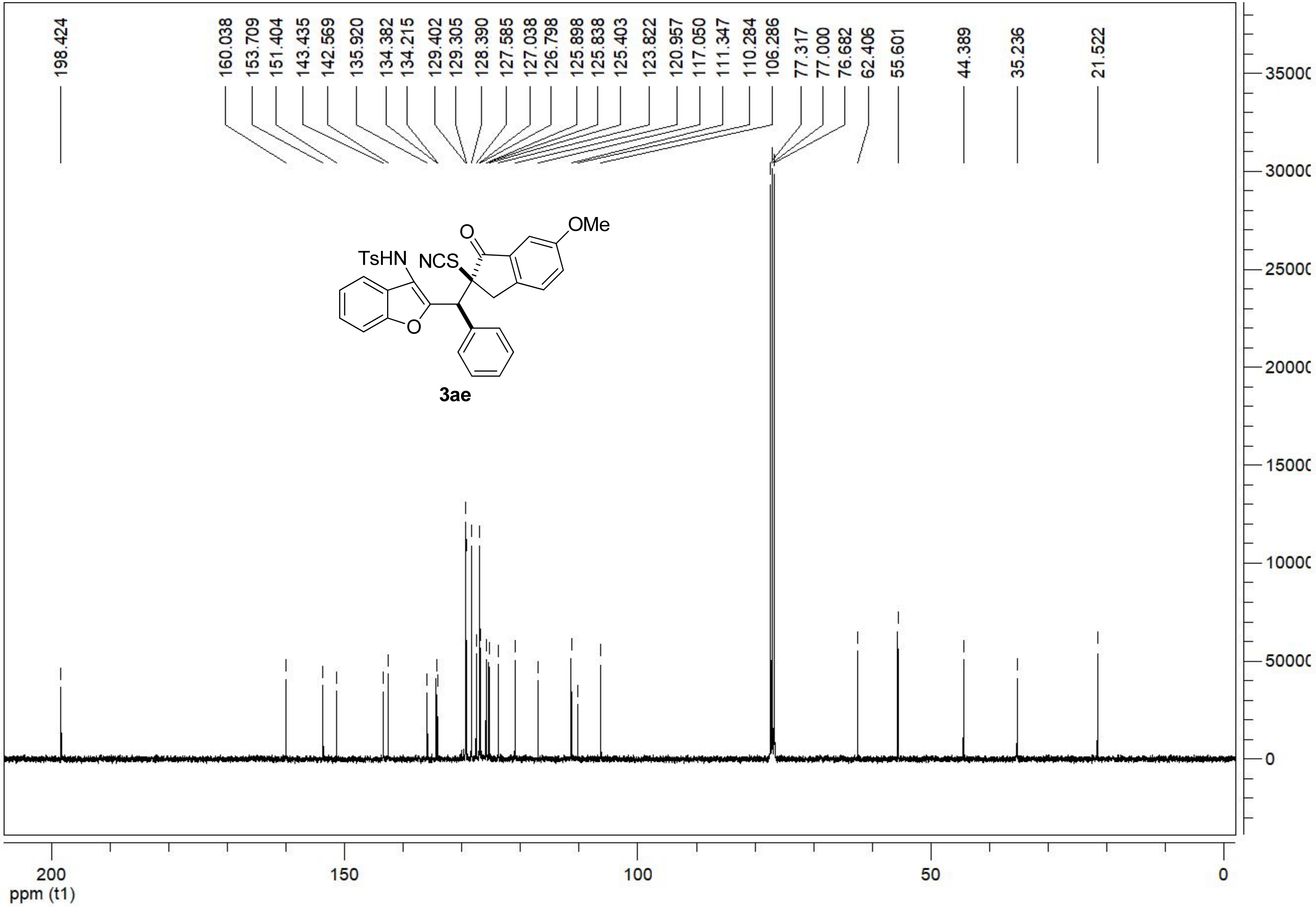
198.424
160.038
153.709
151.404
143.435
142.569
135.920
134.382
134.215
129.402
129.305
128.390
127.585
127.038
126.798
125.898
125.838
125.403
123.822
120.957
117.050
111.347
110.284
106.286
77.317
77.000
76.682
62.406
55.601
44.389
35.236
21.522
TsHN
NCS
O
OMe
O
3ae
ppm (t1)
200
150
100
50
0

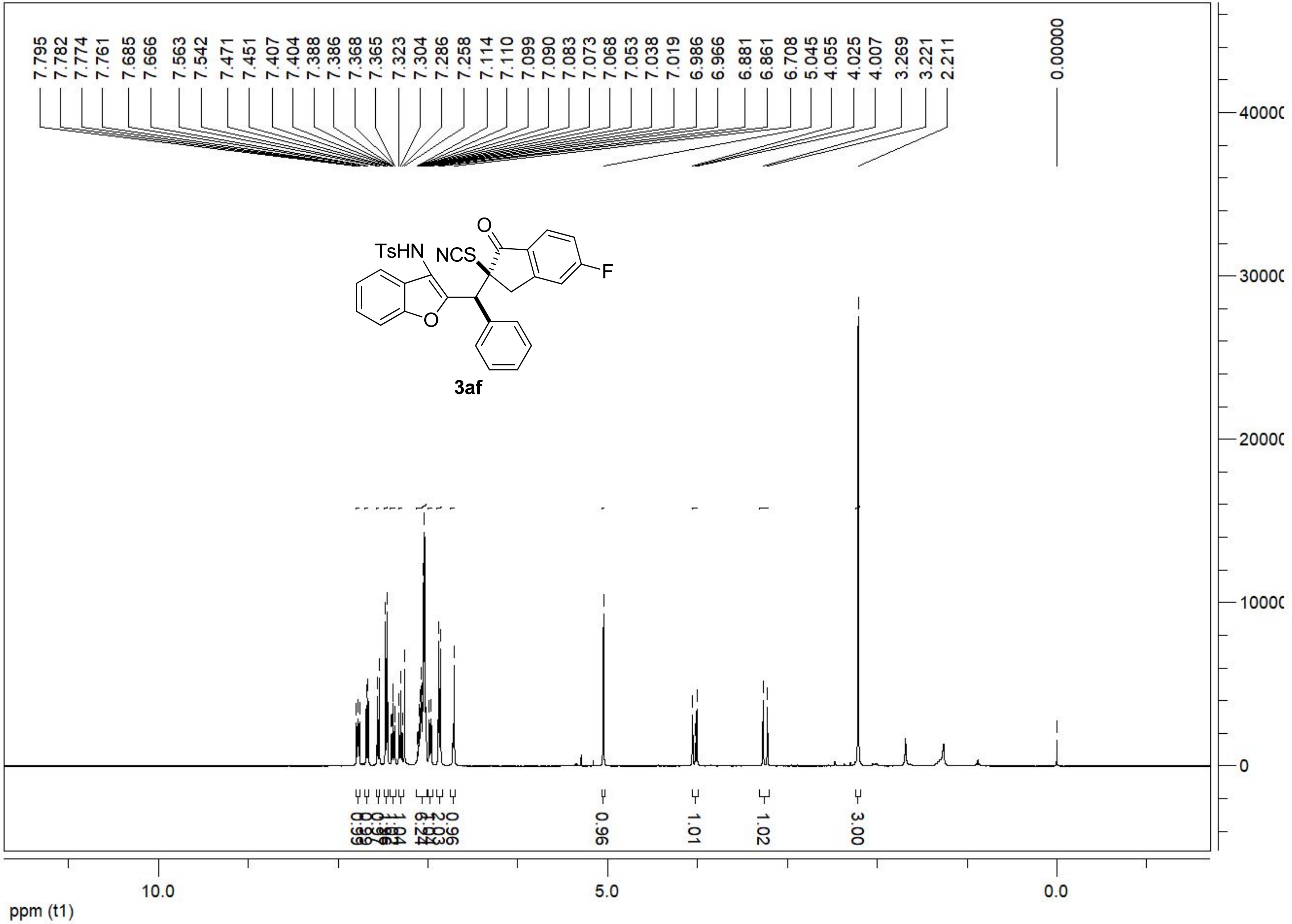
7.795
7.782
7.774
7.761
7.685
7.666
7.563
7.542
7.471
7.451
7.407
7.404
7.388
7.386
7.368
7.365
7.323
7.304
7.286
7.258
7.114
7.110
7.099
7.090
7.083
7.073
7.068
7.053
7.038
7.019
6.986
6.966
6.881
6.861
6.708
5.045
4.055
4.025
4.007
3.269
3.221
2.211
0.00000
TsHN
NCS
O
F
O
3af
40000
30000
20000
10000
0
0.96
1.01
1.02
3.00
10.0
5.0
0.0
ppm (t1)

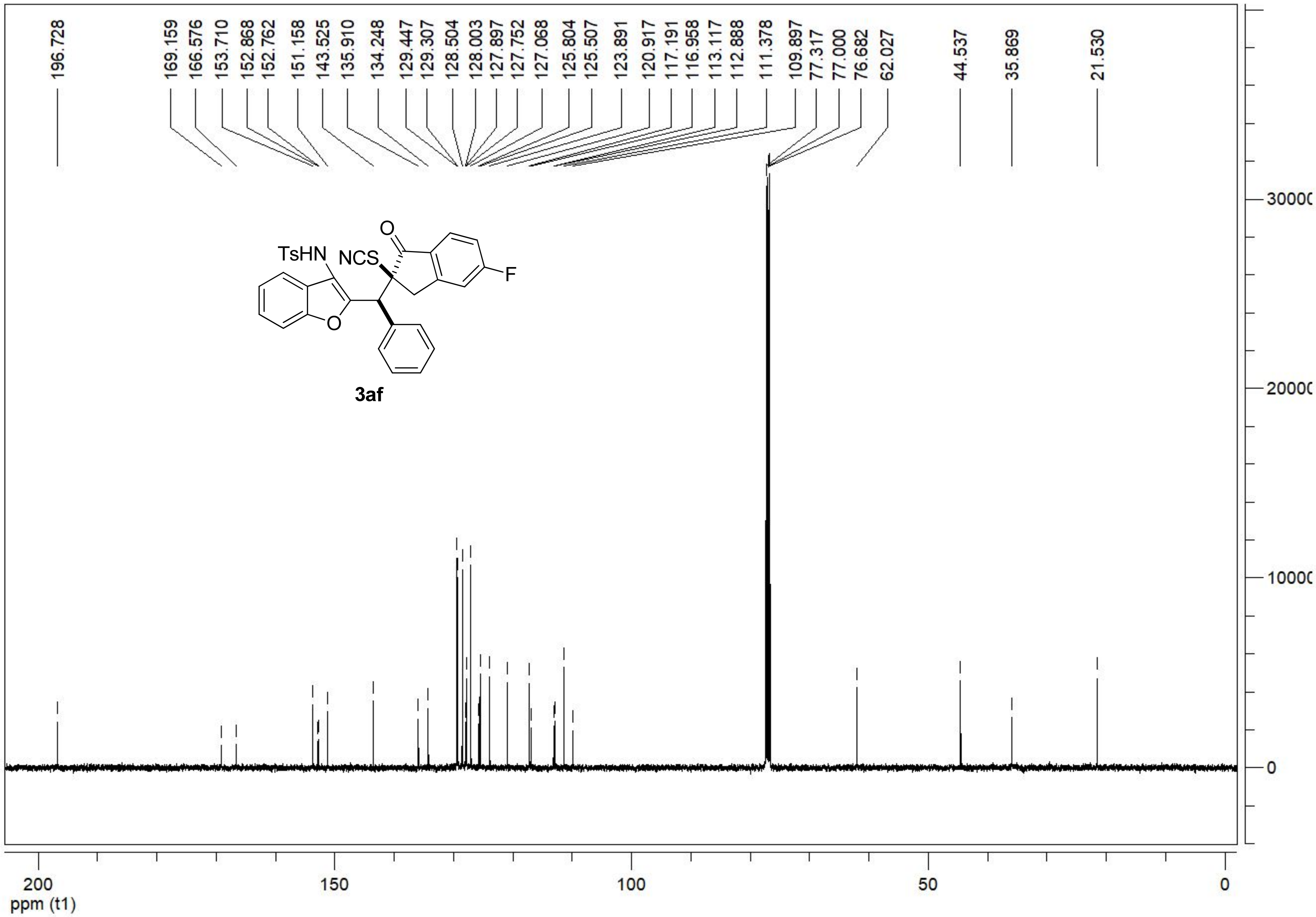
196.728
169.159
166.576
153.710
152.868
152.762
151.158
143.525
135.910
134.248
129.447
129.307
128.504
128.003
127.897
127.752
127.068
125.804
125.507
123.891
120.917
117.191
116.958
113.117
112.888
111.378
109.897
77.317
77.000
76.682
62.027
44.537
35.869
21.530
TsHN
NCS
O
F
3af
30000
20000
10000
0
200
150
100
50
0
ppm (t1)

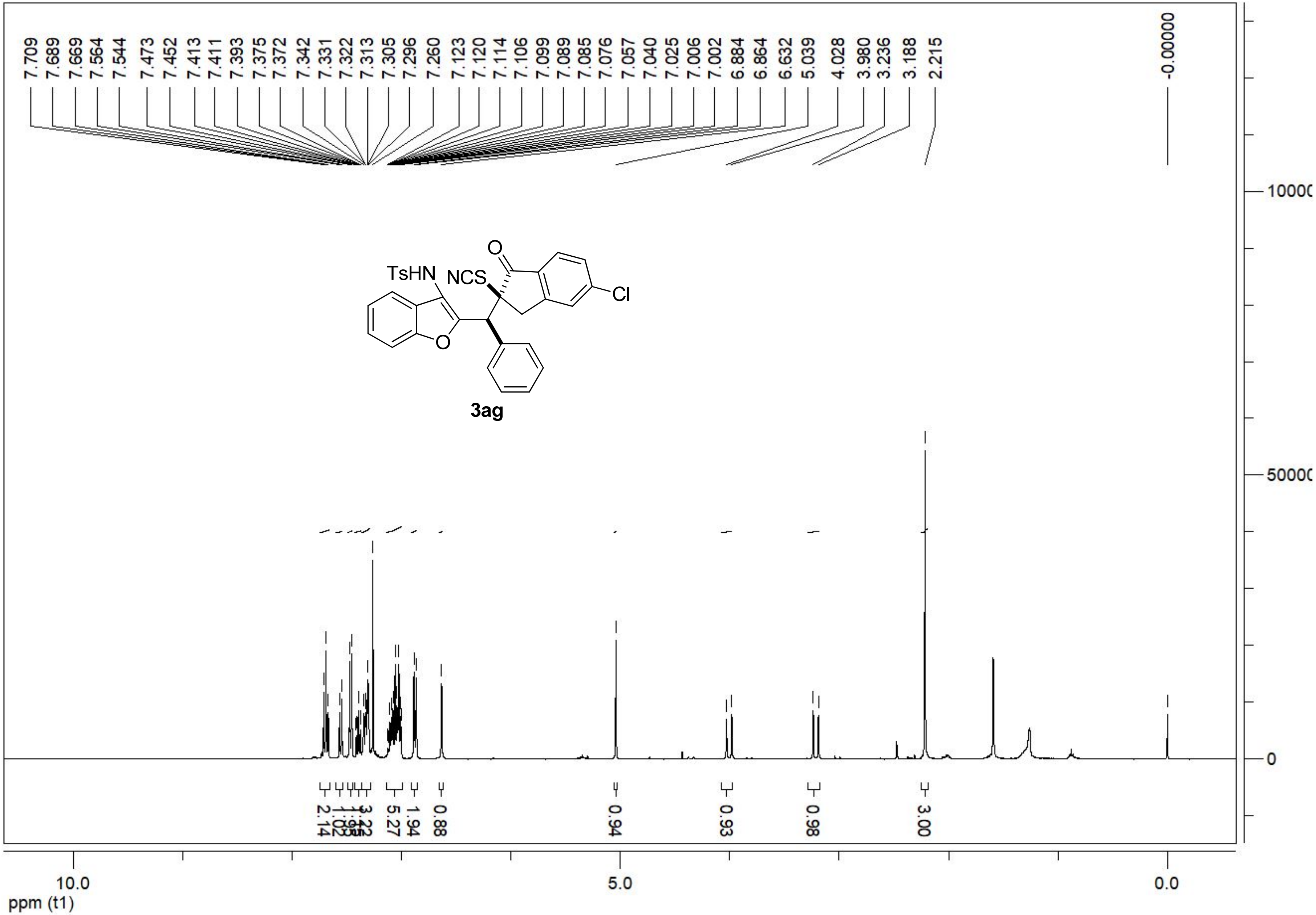

7.709
7.689
7.669
7.564
7.544
7.473
7.452
7.413
7.411
7.393
7.375
7.372
7.342
7.331
7.322
7.313
7.305
7.296
7.260
7.123
7.120
7.114
7.106
7.099
7.089
7.085
7.076
7.057
7.040
7.025
7.006
7.002
6.884
6.864
6.632
5.039
4.028
3.980
3.236
3.188
2.215
-0.00000
TsHN
NCS
O
Cl
3ag
2.14
1.02
3.22
5.27
1.94
0.88
0.94
0.93
0.98
3.00
10.0
5.0
0.0
ppm (t1)

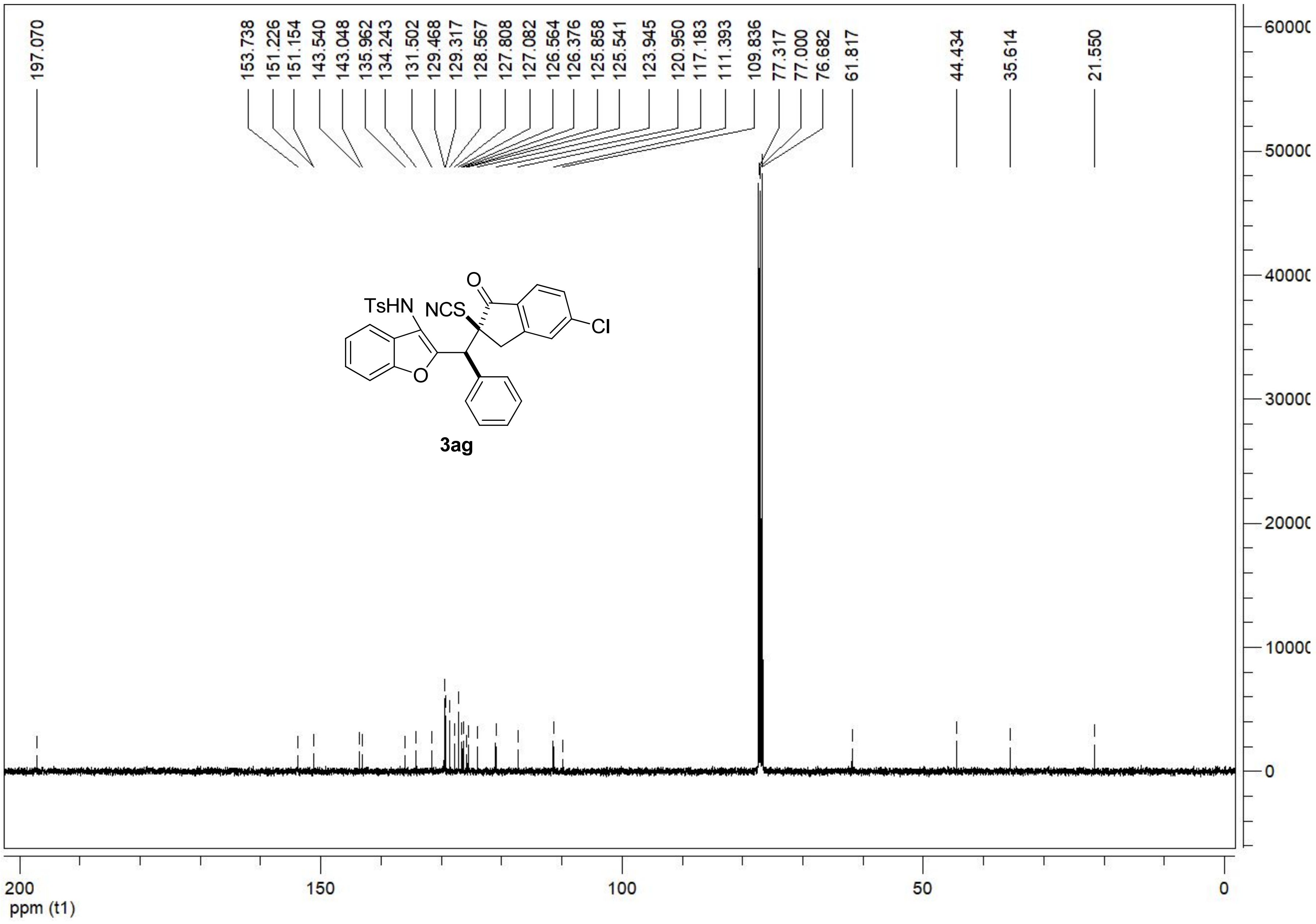
197.070
153.738
151.226
151.154
143.540
143.048
135.962
134.243
131.502
129.468
129.317
128.567
127.808
127.082
126.564
126.376
125.858
125.541
123.945
120.950
117.183
111.393
109.836
77.317
77.000
76.682
61.817
44.434
35.614
21.550
TsHN
NCS
O
Cl
3ag
200
150
100
50
0
ppm (t1)

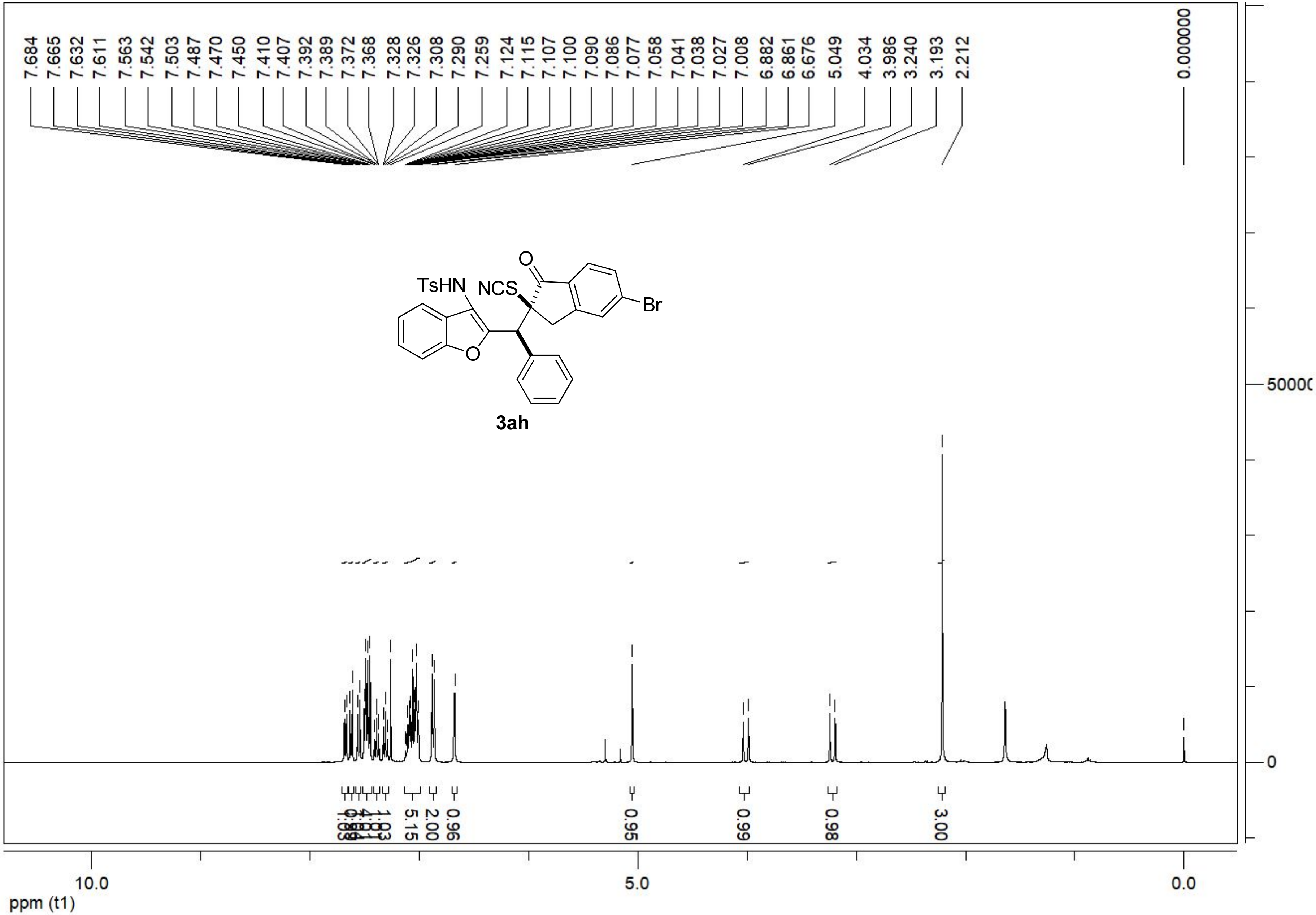
7.684 7.665 7.632 7.611 7.563 7.542 7.503 7.487 7.470 7.450 7.410 7.407 7.392 7.389 7.372 7.368 7.328 7.326 7.308 7.290 7.259 7.124 7.115 7.107 7.100 7.090 7.086 7.077 7.058 7.041 7.038 7.027 7.008 6.882 6.861 6.676 5.049 4.034 3.986 3.240 3.193 2.212
0.000000
TsHN
NCS
O
Br
3ah
5.15
2.00
0.96
0.95
0.99
0.98
3.00
50000
0
10.0
5.0
0.0
ppm (t1)

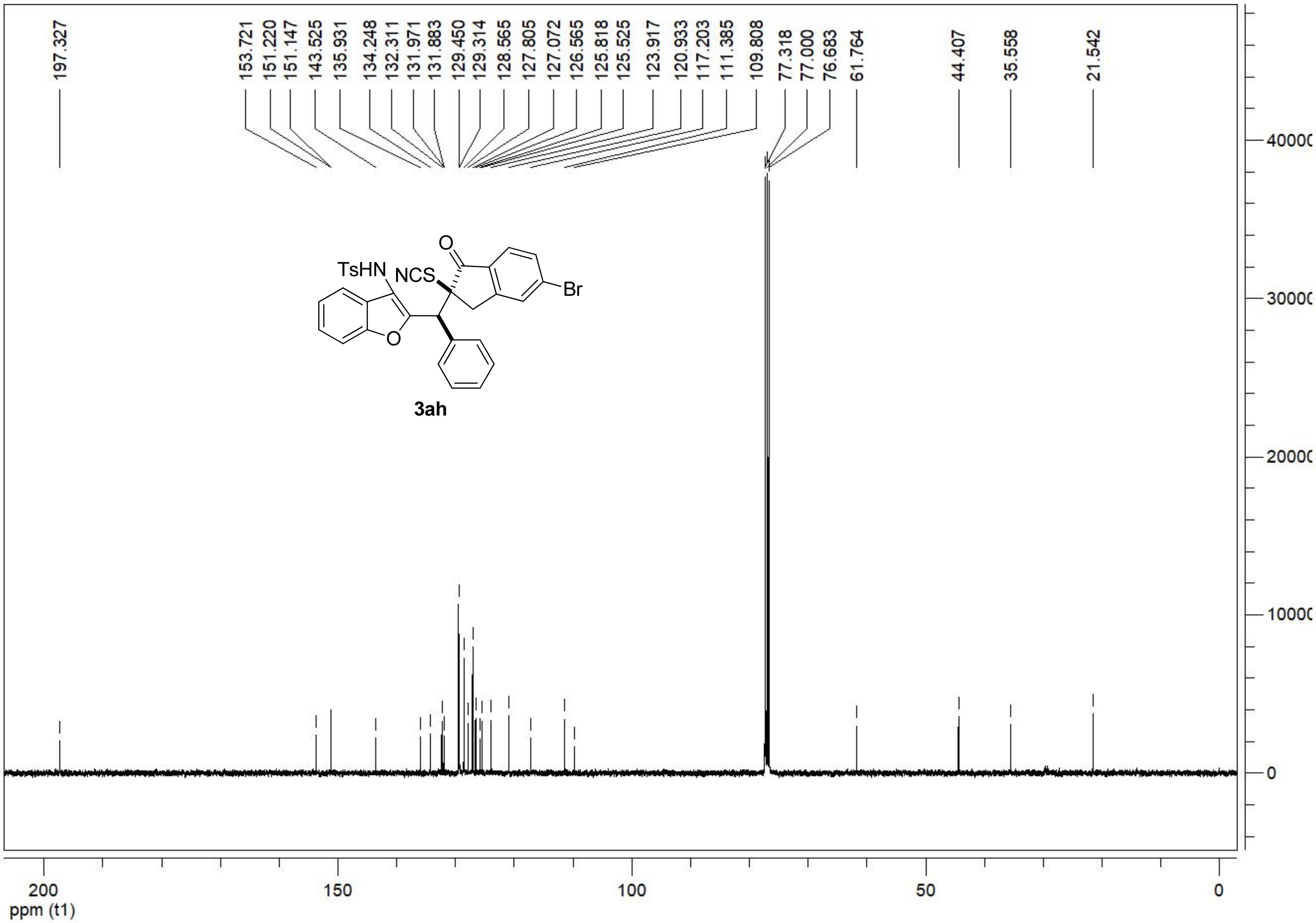
197.327
153.721
151.220
151.147
143.525
135.931
134.248
132.311
131.971
131.883
129.450
129.314
128.565
127.805
127.072
126.565
125.818
125.525
123.917
120.933
117.203
111.385
109.808
77.318
77.000
76.683
61.764
44.407
35.558
21.542
TsHN
NCS
O
Br
O
3ah
ppm (t1)

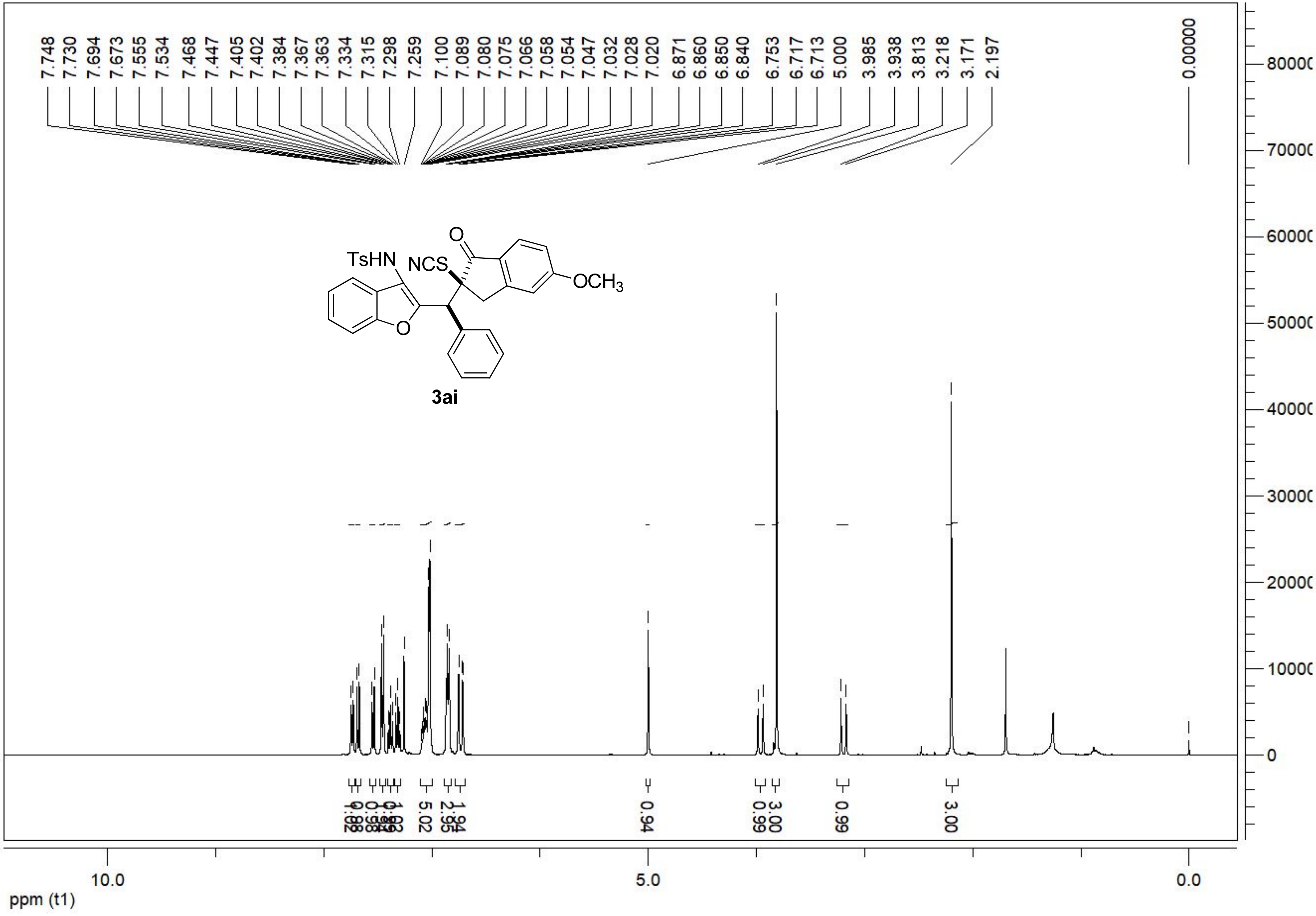
7.748 7.730 7.694 7.673 7.555 7.534 7.468 7.447 7.405 7.402 7.384 7.367 7.363 7.334 7.315 7.298 7.259 7.100 7.089 7.080 7.075 7.066 7.058 7.054 7.047 7.032 7.028 7.020 6.871 6.860 6.850 6.840 6.753 6.717 6.713 5.000 3.985 3.938 3.813 3.218 3.171 2.197
0.00000
TsHN
NCS
O
OCH3
O
3ai
5.02
2.95
1.94
0.94
0.99
3.00
0.99
3.00
80000
70000
60000
50000
40000
30000
20000
10000
0
10.0
5.0
0.0
ppm (t1)

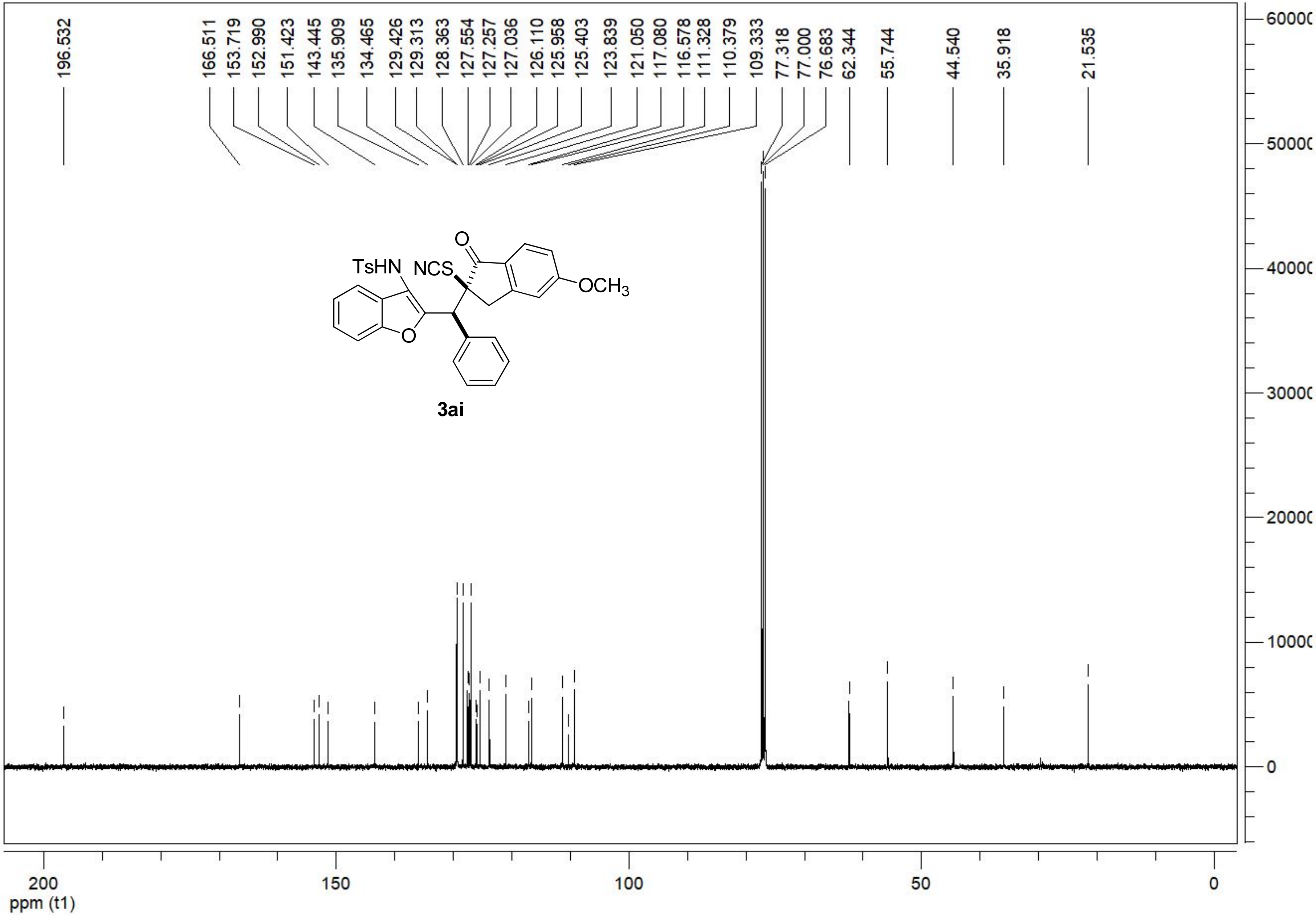
196.532
166.511
153.719
152.990
151.423
143.445
135.909
134.465
129.426
129.313
128.363
127.554
127.257
127.036
126.110
125.958
125.403
123.839
121.050
117.080
116.578
111.328
110.379
109.333
77.318
77.000
76.683
62.344
55.744
44.540
35.918
21.535
TsHN
NCS
O
OCH3
3ai
60000
50000
40000
30000
20000
10000
0
200
150
100
50
0
ppm (t1)

2. X-ray single-crystal data for product 3ja

The single crystal of **3ja** was cultured in a mixture of ethyl acetate and petroleum ether (1:3), The single crystal data see Table S1.

Table S1 Crystal data and structure refinement for **3ja**

Identification code	a_a
Empirical formula	$C_{36}H_{26}N_2O_4S_2$
Formula weight	614.71
Temperature/K	273.15
Crystal system	triclinic
Space group	P-1
a/Å	9.1074(11)
b/Å	13.1125(15)
c/Å	14.3704(17)
α/°	107.532(6)
β/°	98.113(6)
γ/°	108.346(6)
Volume/Å^3	1499.5(3)
Z	2
ρ_{calc}g/cm^3	1.361
μ/mm^{-1}	1.968
F(000)	640.0
Crystal size/mm^3	0.21 × 0.2 × 0.18
Radiation	CuKα (λ = 1.54178)
2Θ range for data collection/°	6.682 to 137.042
Index ranges	$-10 \le h \le 9$, $-15 \le k \le 15$, $-17 \le l \le 17$
Reflections collected	38808
Independent reflections	5480 [R_{int} = 0.1301, R_{sigma} = 0.0829]
Data/restraints/parameters	5480/564/398
Goodness-of-fit on F^2	1.069
Final R indexes [I>=2σ (I)]	R_1 = 0.0563, wR_2 = 0.1514
Final R indexes [all data]	R_1 = 0.1193, wR_2 = 0.1873
Largest diff. peak/hole / e Å^{-3}	0.42/-0.39

3. Copies of HPLC chromatograms

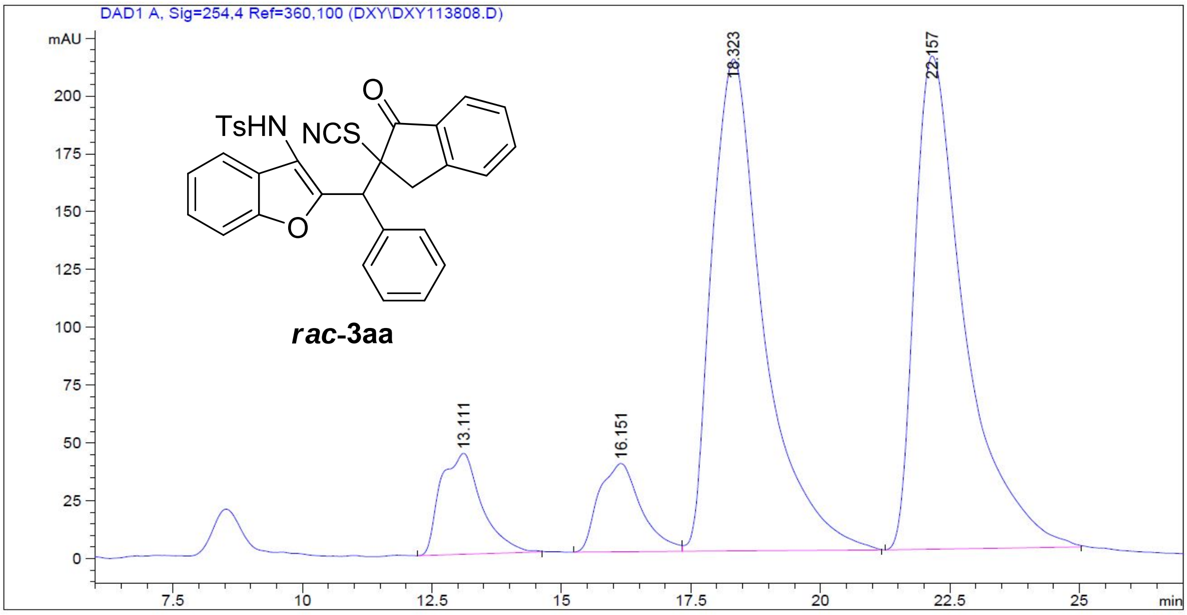

Peak #	RetTime [min]	Type	Width [min]	Area [mAU*s]	Height [mAU]	Area %
1	13.111	BB	0.7182	2411.73242	43.67733	7.1623
2	16.151	BV	0.7534	2166.38062	38.21144	6.4336
3	18.323	VB	0.9789	1.47881e4	212.76341	43.9172
4	22.157	BB	0.9867	1.43065e4	213.25284	42.4869

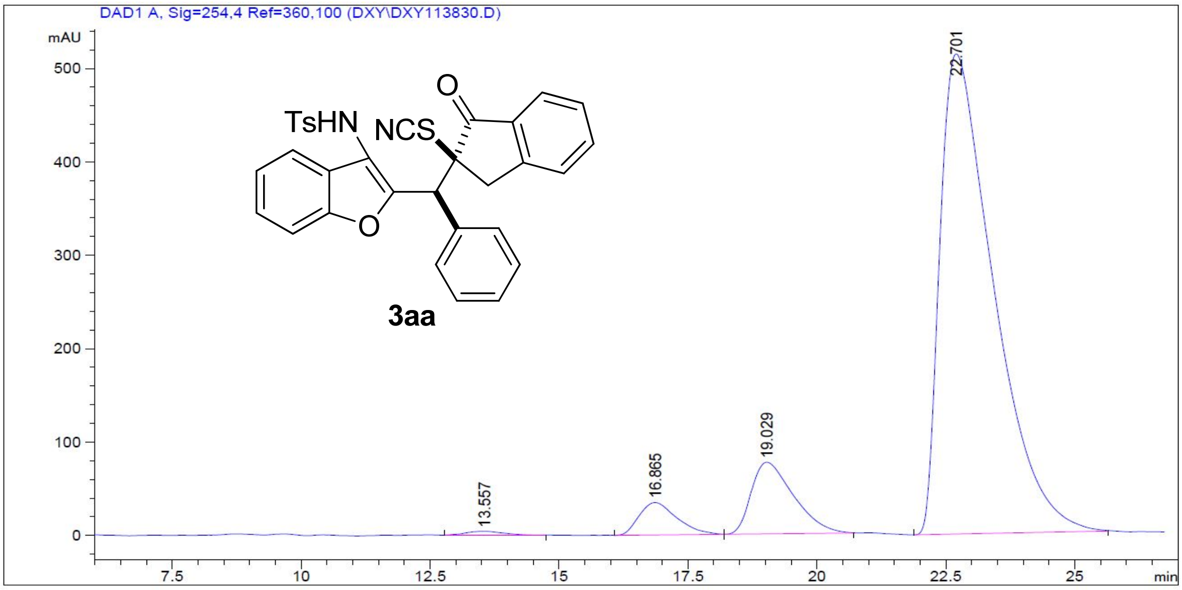

Peak #	RetTime [min]	Type	Width [min]	Area [mAU*s]	Height [mAU]	Area %
1	13.557	MM	0.5617	121.00778	3.59028	0.2775
2	16.865	MM	0.8021	1644.76990	34.17732	3.7715
3	19.029	MM	0.9097	4135.89502	75.77113	9.4838
4	22.701	MM	1.2132	3.77085e4	518.02844	86.4672

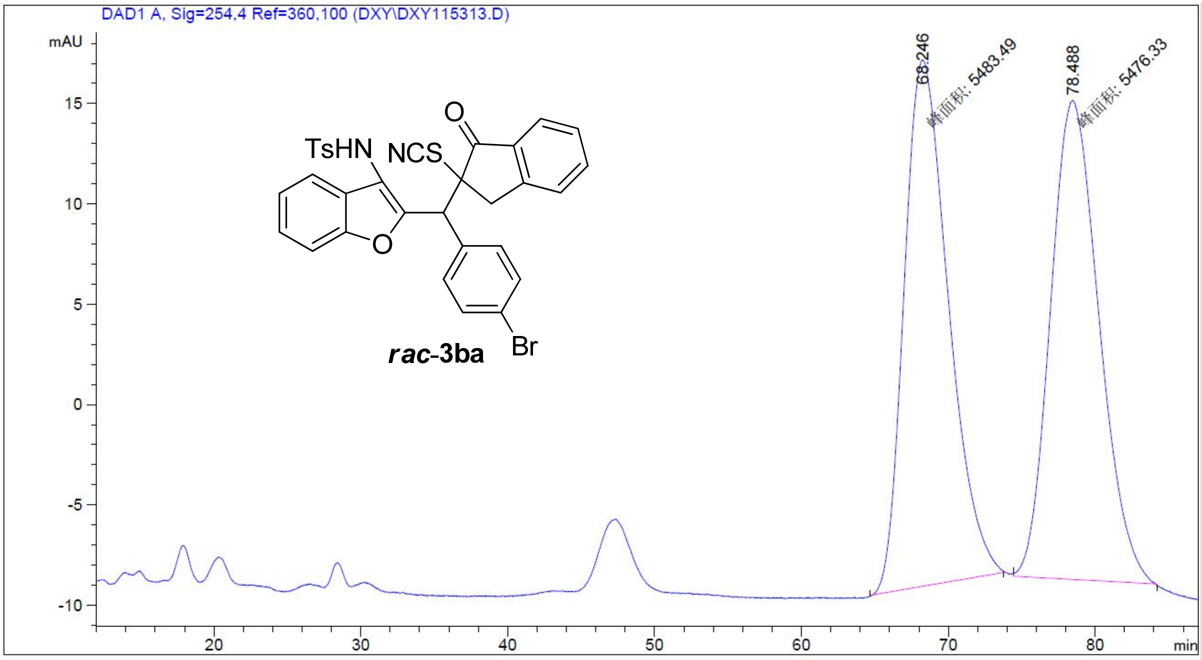

Peak #	RetTime [min]	Type	Width [min]	Area [mAU*s]	Height [mAU]	Area %
1	68.246	MM	3.5384	5634.02490	26.53769	50.0659
2	78.488	MM	3.8929	5619.18896	24.05749	49.9341

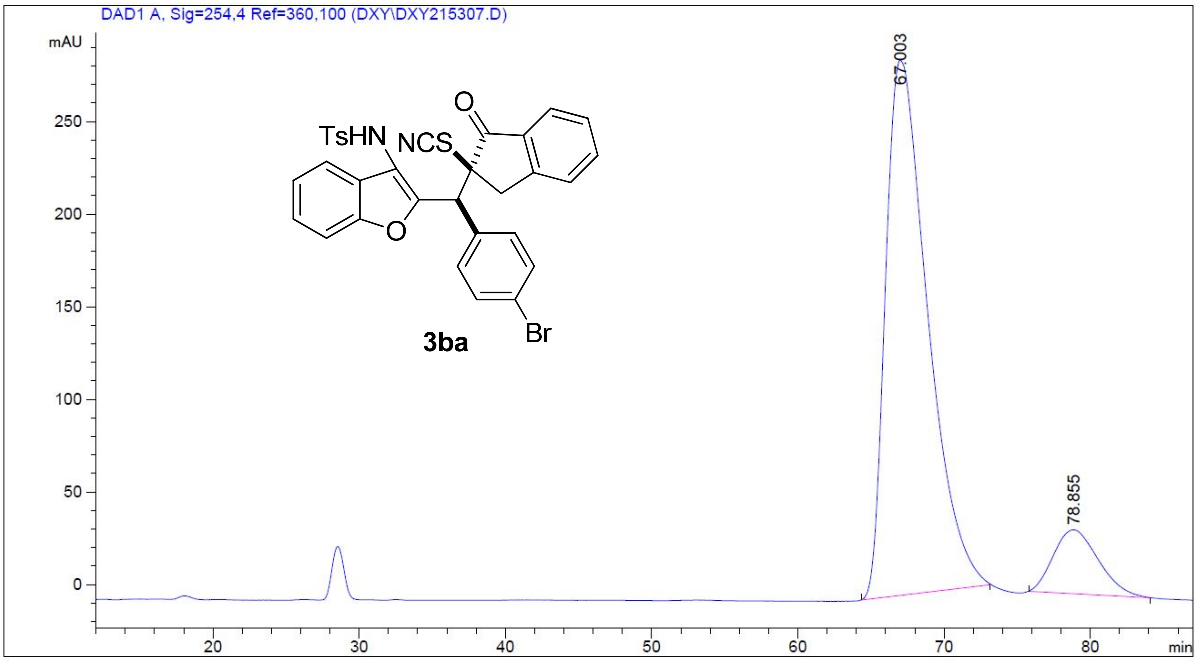

Peak #	RetTime [min]	Type	Width [min]	Area [mAU*s]	Height [mAU]	Area %
1	67.003	MM	3.4050	5.94125e4	290.80991	88.5516
2	78.859	MM	3.6202	7681.11475	35.36198	11.4484

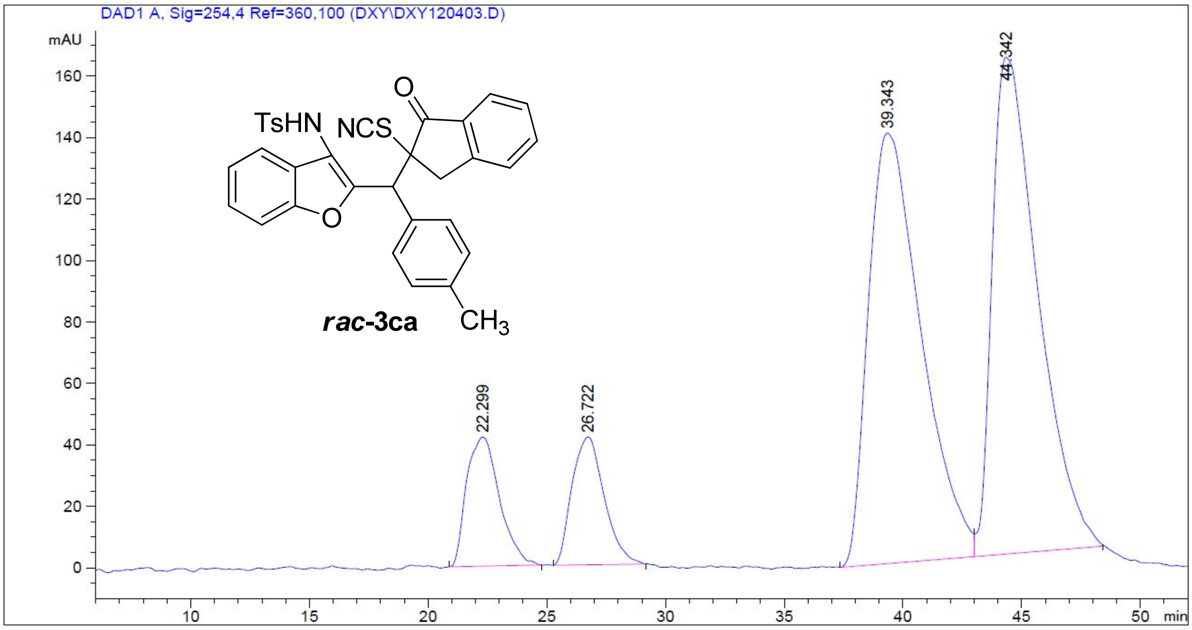

Peak #	RetTime [min]	Type	Width [min]	Area [mAU*s]	Height [mAU]	Area %
1	22.299	BB	1.1513	4046.34448	41.94951	7.8951
2	26.722	BB	1.1272	3933.47998	41.52397	7.6749
3	39.343	BV	1.8168	2.16170e4	140.04588	42.1786
4	44.342	VB	1.7535	2.16543e4	161.57863	42.2513

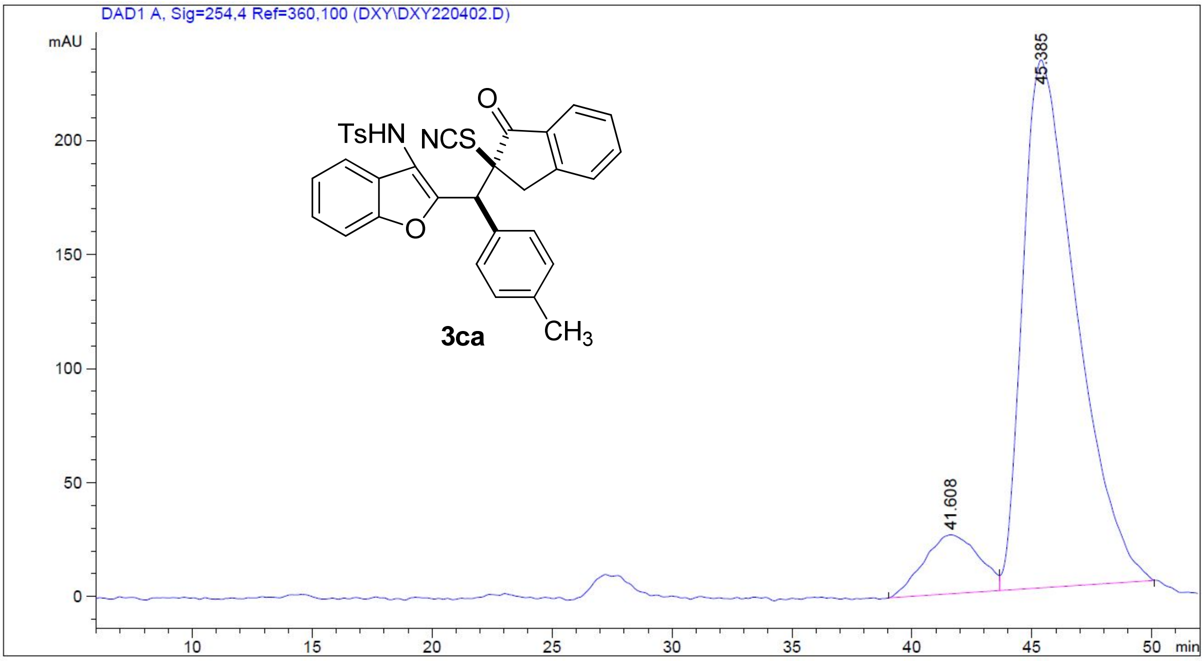

Peak #	RetTime [min]	Type	Width [min]	Area [mAU*s]	Height [mAU]	Area %
1	41.608	BV	1.8705	4130.06201	25.92630	10.1248
2	45.385	MM	2.6324	3.66615e4	232.11403	89.8752

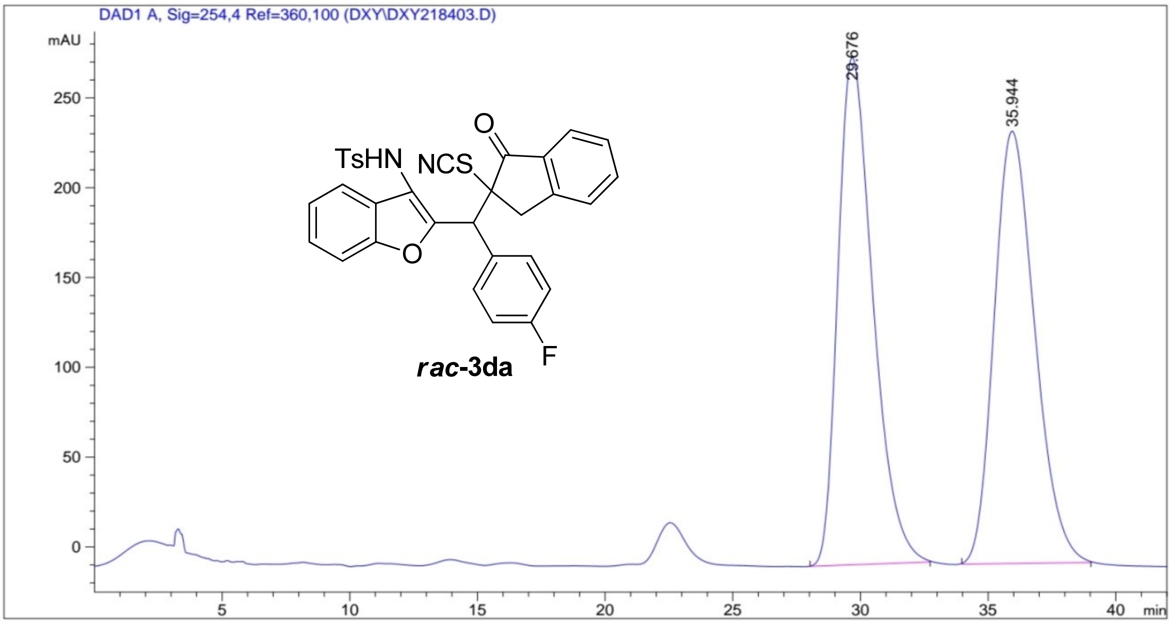

Peak #	RetTime [min]	Type	Width [min]	Area [mAU*s]	Height [mAU]	Area %
1	29.676	BB	1.3962	2.69336e4	282.51431	50.2563
2	35.944	BB	1.6441	2.66589e4	240.71315	49.7437

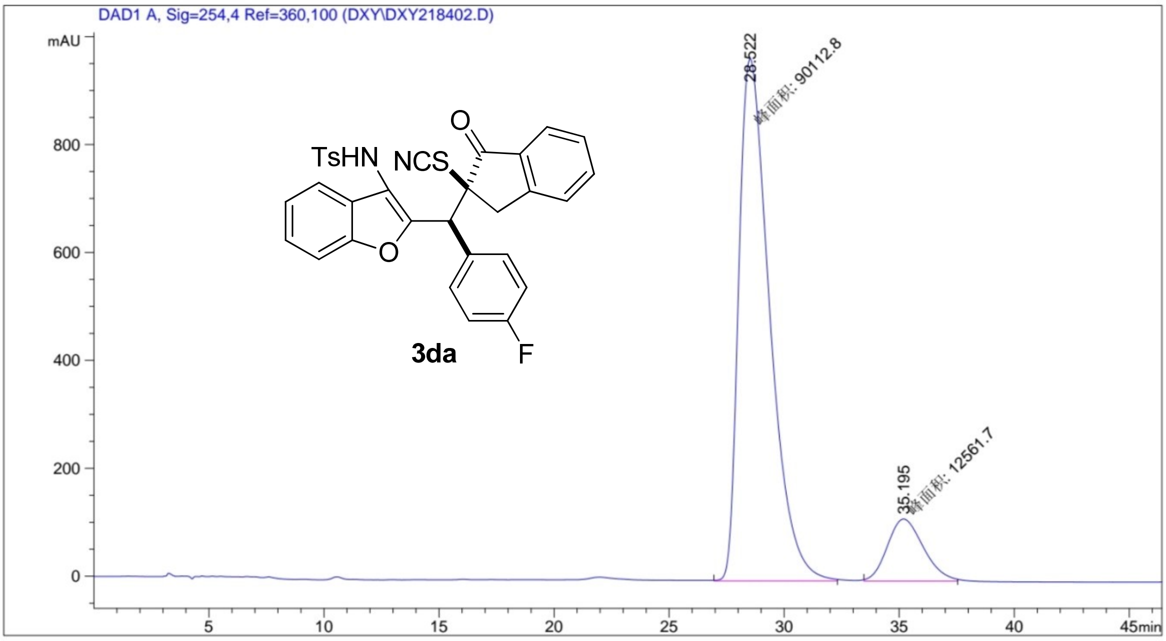

Peak #	RetTime [min]	Type	Width [min]	Area [mAU*s]	Height [mAU]	Area %
1	28.522	MM	1.5521	9.01128e4	967.64630	87.7655
2	35.195	MM	1.8176	1.25617e4	115.18518	12.2345

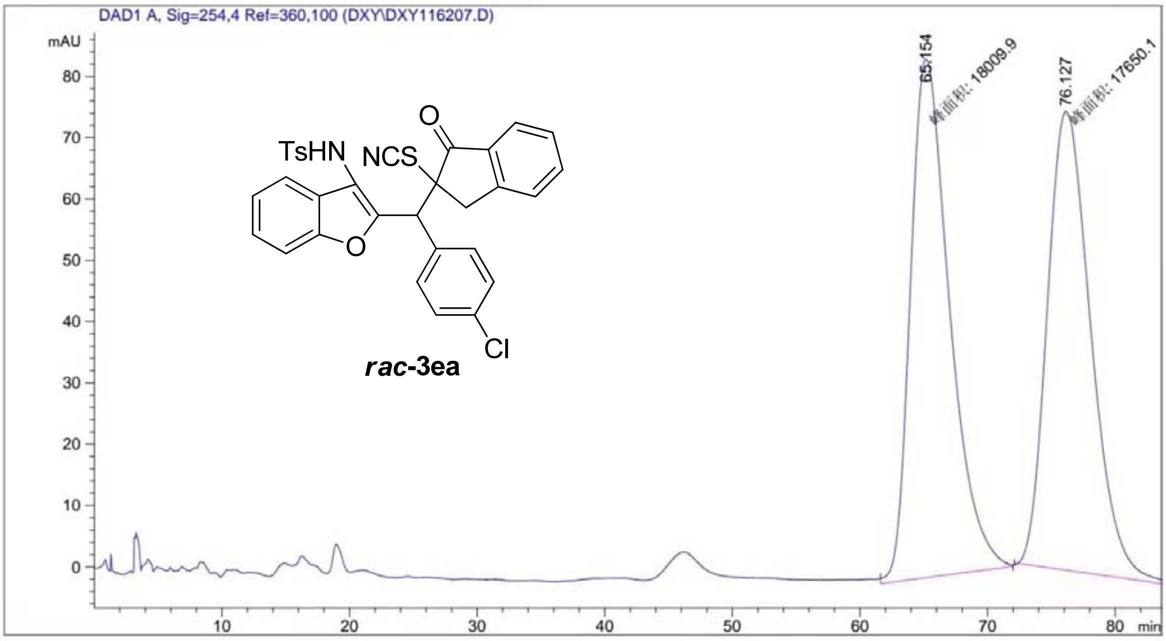

Peak #	RetTime [min]	Type	Width [min]	Area [mAU*s]	Height [mAU]	Area %
1	65.154	BB	2.3816	1.68020e4	82.58620	49.7546
2	76.115	BB	2.7030	1.69678e4	73.89217	50.2454

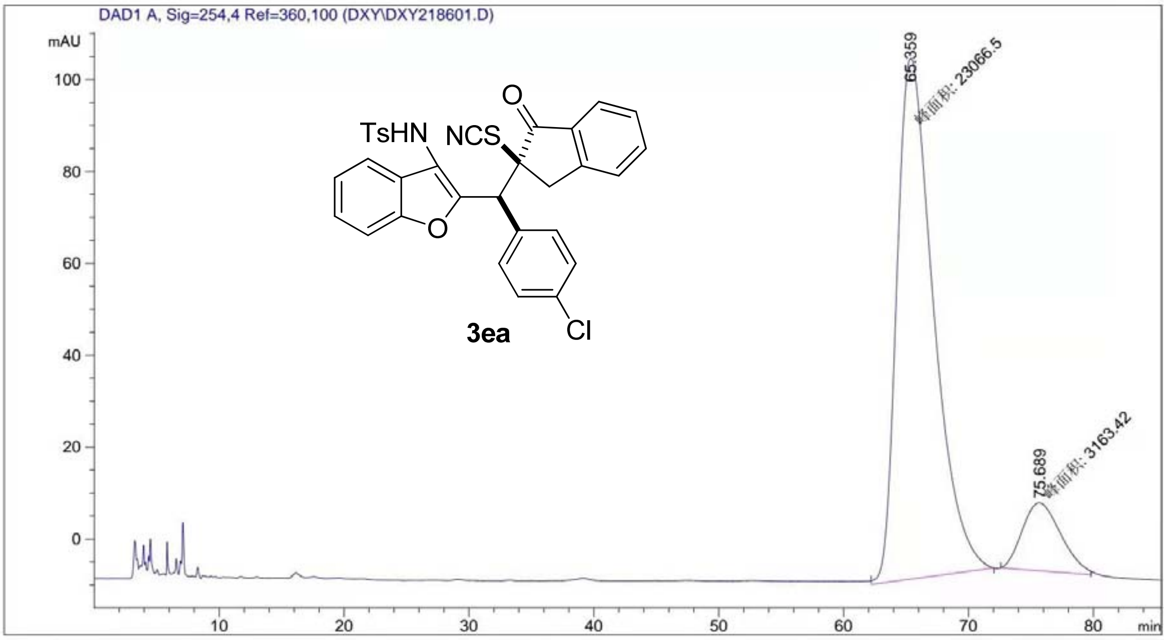

Peak #	RetTime [min]	Type	Width [min]	Area [mAU*s]	Height [mAU]	Area %
1	65.359	MM	3.3932	2.30665e4	113.29842	87.9396
2	75.689	MM	3.5652	3163.42480	14.78861	12.0604

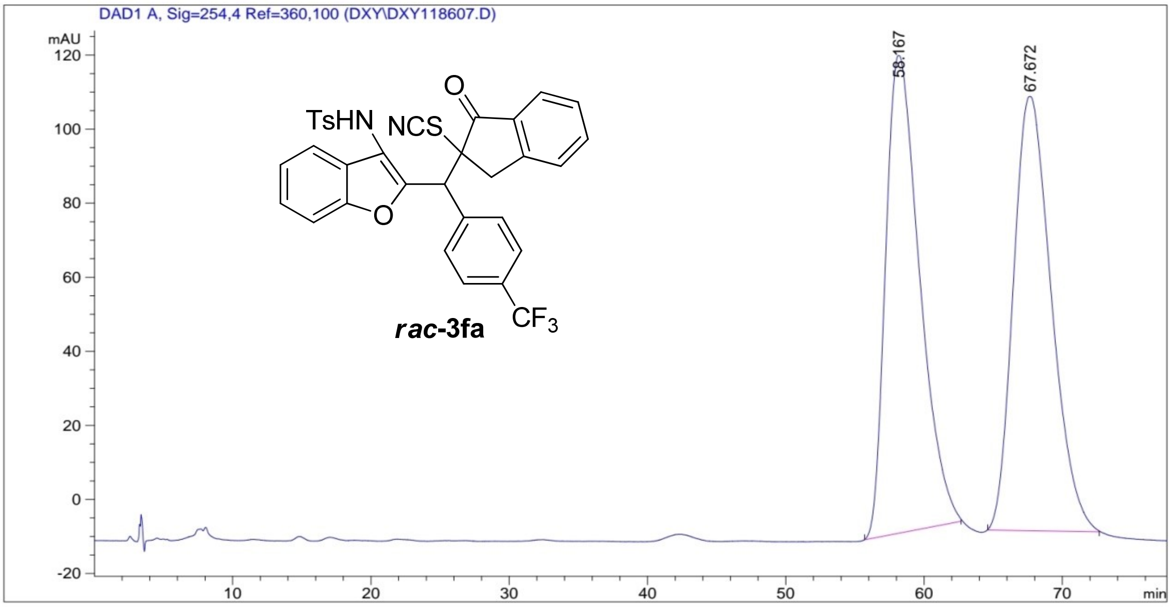

Peak #	RetTime [min]	Type	Width [min]	Area [mAU*s]	Height [mAU]	Area %
1	58.167	BB	2.0023	2.19618e4	128.99261	49.5073
2	67.672	BB	2.2400	2.23990e4	117.29044	50.4927

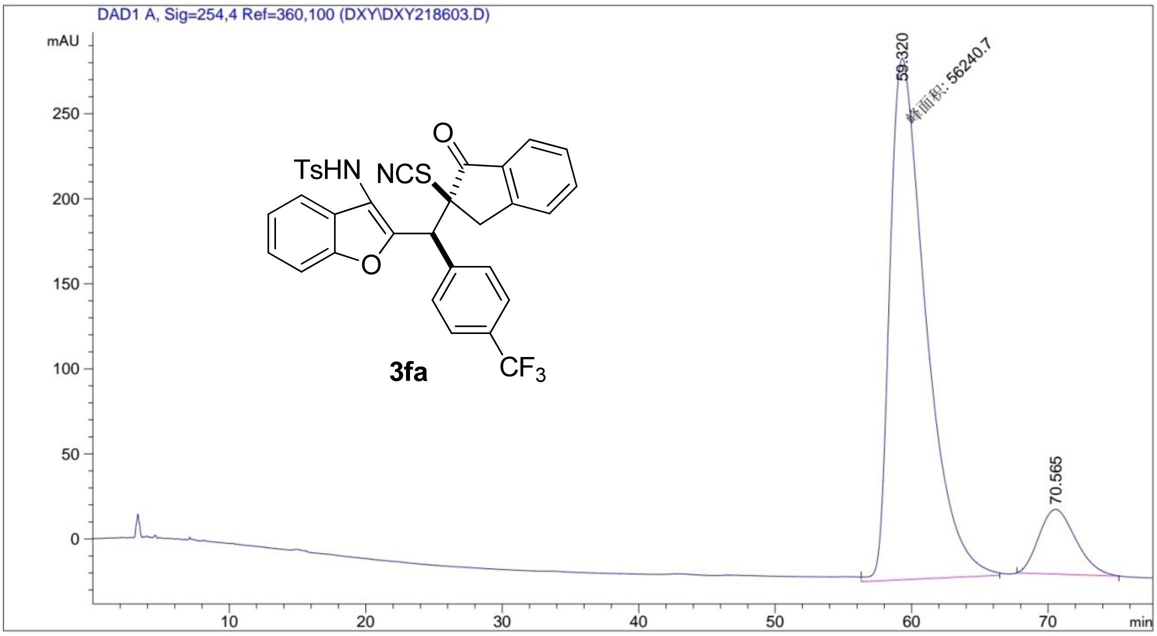

Peak #	RetTime [min]	Type	Width [min]	Area [mAU*s]	Height [mAU]	Area %
1	59.320	MM	3.0570	5.62407e4	306.62296	88.7083
2	70.565	BB	2.2030	7158.88623	38.15503	11.2917

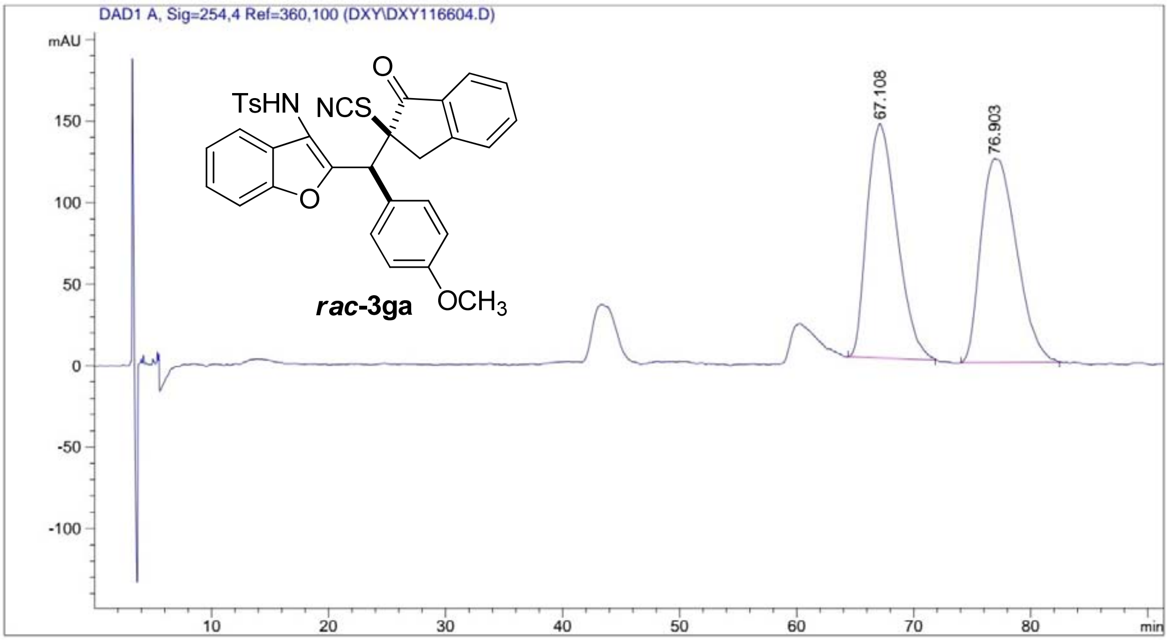

Peak #	RetTime [min]	Type	Width [min]	Area [mAU*s]	Height [mAU]	Area %
1	67.108	BB	2.1584	2.56083e4	143.60931	49.1490
2	76.903	BB	2.4802	2.64951e4	124.93356	50.8510

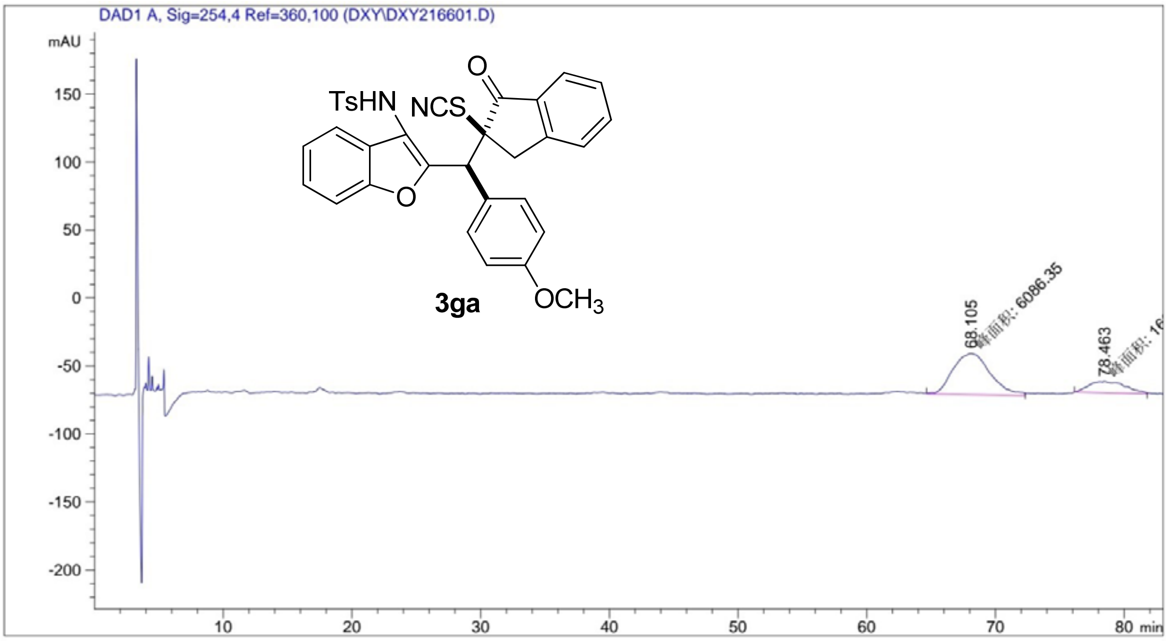

Peak #	RetTime [min]	Type	Width [min]	Area [mAU*s]	Height [mAU]	Area %
1	68.105	MM	3.3518	6086.35059	30.26385	78.5444
2	78.463	MM	3.2779	1662.58276	8.45343	21.4556

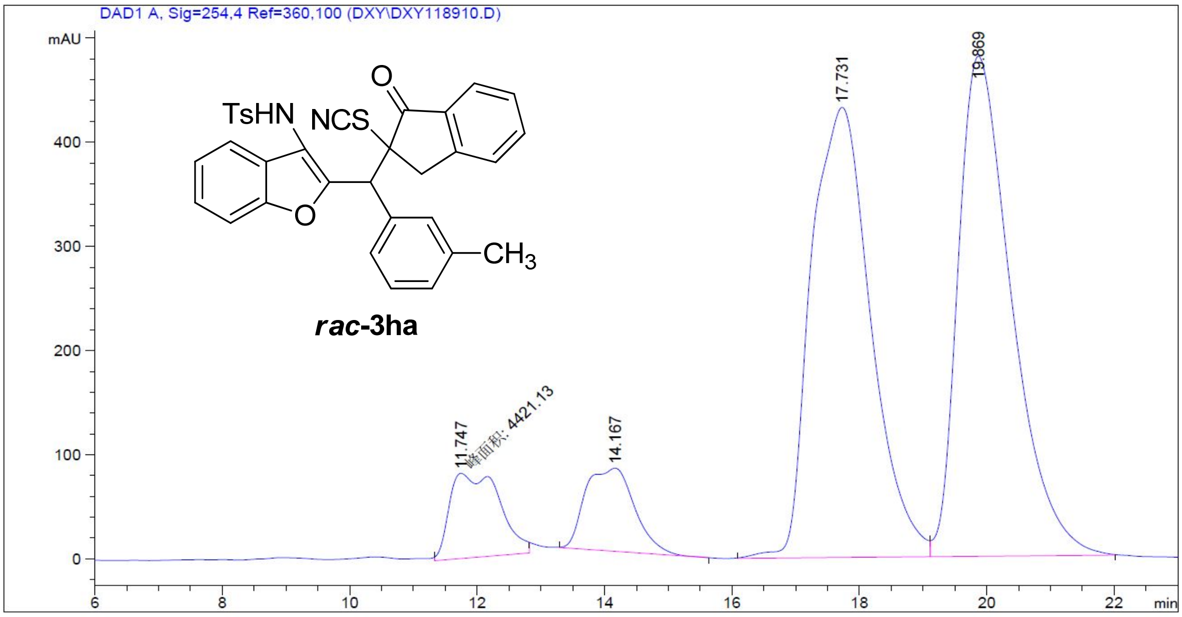

Peak #	RetTime [min]	Type	Width [min]	Area [mAU*s]	Height [mAU]	Area %
1	11.747	MM	0.8515	4129.92432	80.83656	6.4005
2	14.167	BB	0.7281	4274.69238	79.90782	6.6248
3	17.731	BV	0.8995	2.81277e4	432.05380	43.5917
4	19.869	VB	0.8883	2.79930e4	480.05524	43.3830

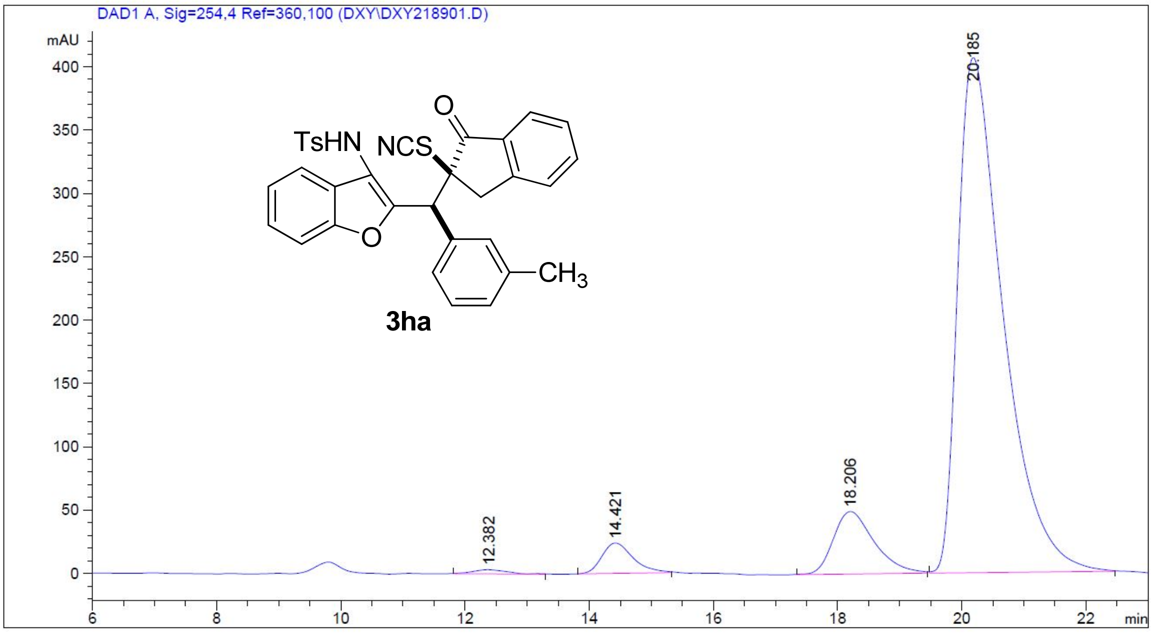

Peak #	RetTime [min]	Type	Width [min]	Area [mAU*s]	Height [mAU]	Area %
1	12.382	BB	0.4411	119.16027	3.27024	0.4990
2	14.421	BB	0.5085	840.70428	24.00974	3.5203
3	18.206	BB	0.6752	2267.09204	49.42046	9.4930
4	20.185	BB	0.7585	2.06547e4	406.72318	86.4877

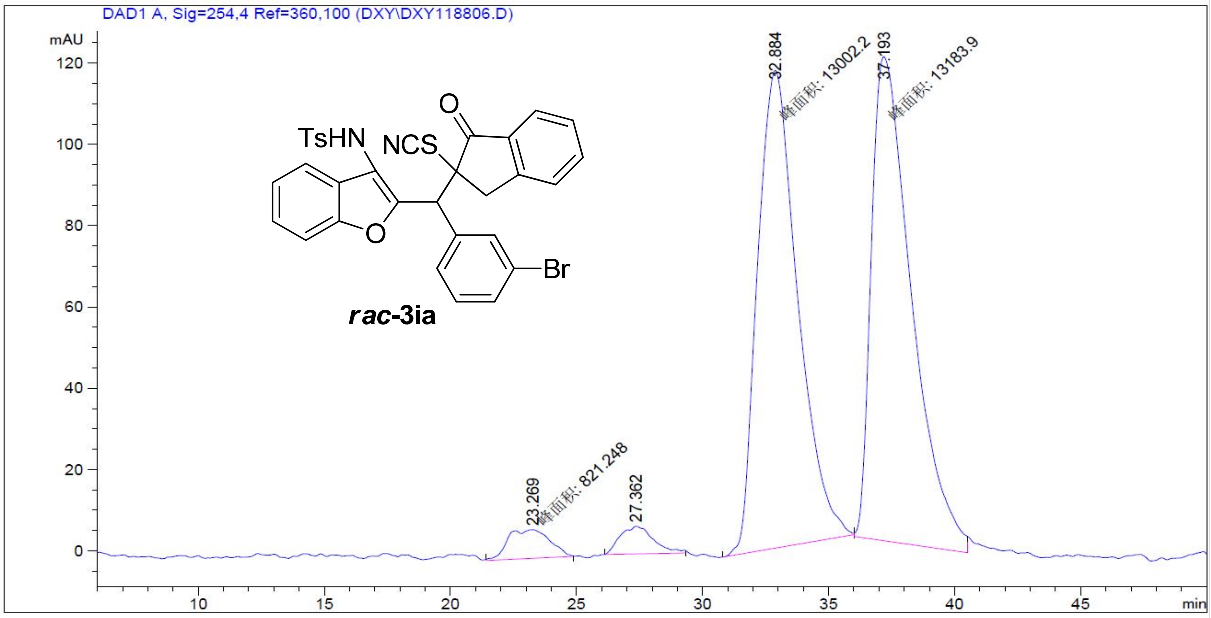

Peak #	RetTime [min]	Type	Width [min]	Area [mAU*s]	Height [mAU]	Area %
1	23.269	MM	1.9472	821.24829	7.02929	2.9718
2	27.362	BB	1.0974	627.74603	6.82260	2.2716
3	32.884	MM	1.8436	1.30022e4	117.54555	47.0497
4	37.193	MM	1.8450	1.31839e4	119.09746	47.7070

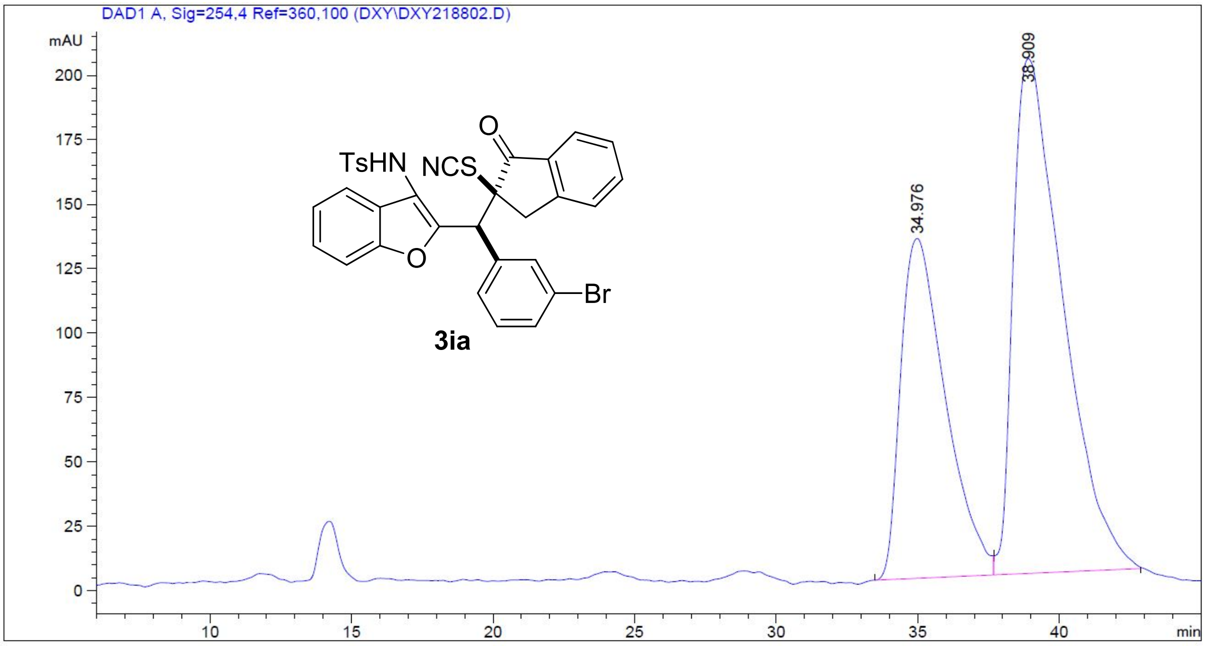

Peak #	RetTime [min]	Type	Width [min]	Area [mAU*s]	Height [mAU]	Area %
1	34.976	MM	1.8059	1.45750e4	134.50984	37.4221
2	38.914	MM	2.0529	2.43726e4	197.87558	62.5779

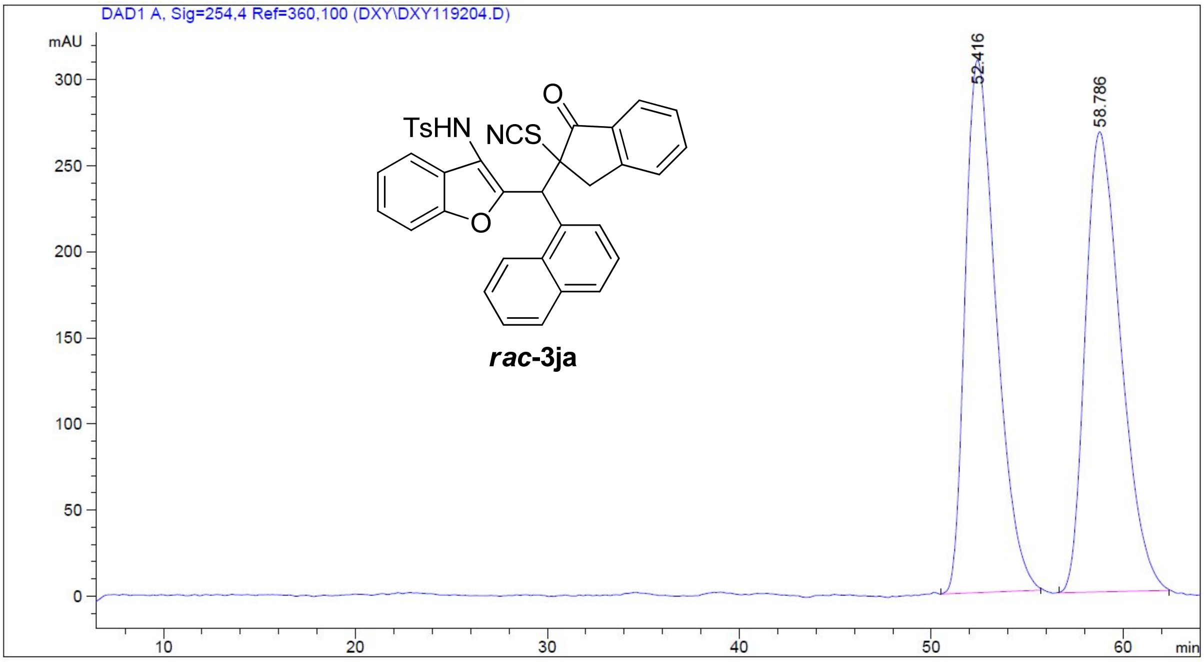

Peak #	RetTime [min]	Type	Width [min]	Area [mAU*s]	Height [mAU]	Area %
1	52.416	BB	1.6173	3.45682e4	309.43335	50.1030
2	58.786	BB	1.7537	3.44261e4	267.09976	49.8970

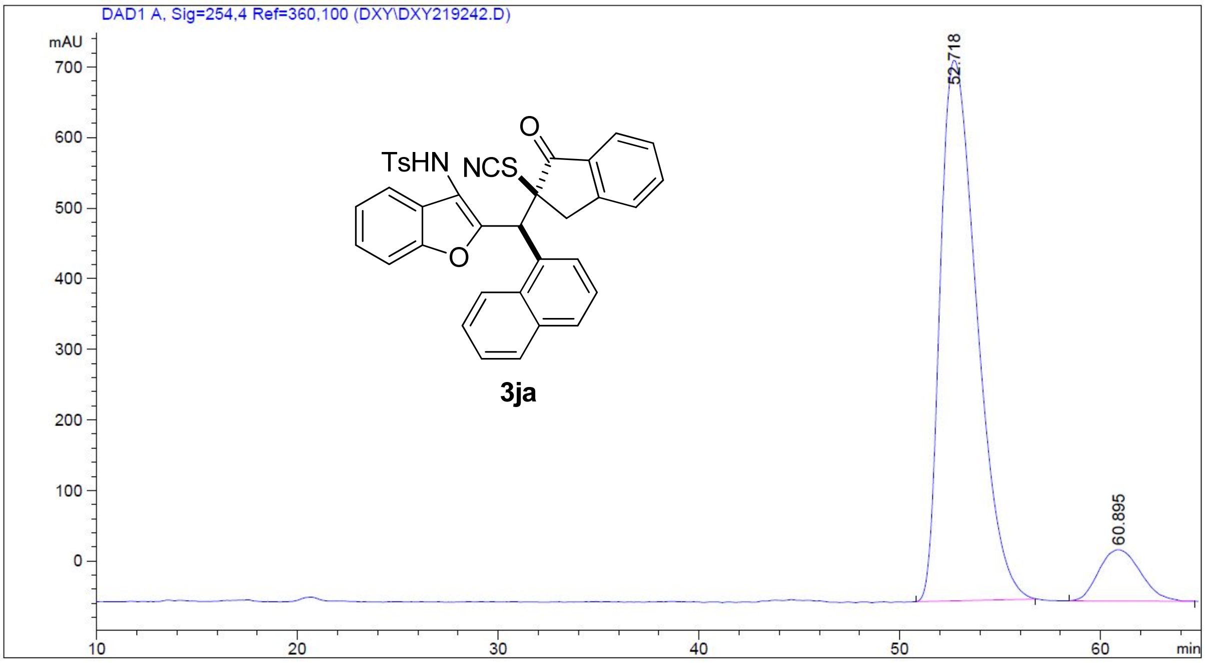

Peak #	RetTime [min]	Type	Width [min]	Area [mAU*s]	Height [mAU]	Area %
1	52.718	BB	1.7452	9.69559e4	765.75183	90.2594
2	60.895	BB	1.6937	1.04633e4	72.76312	9.7406

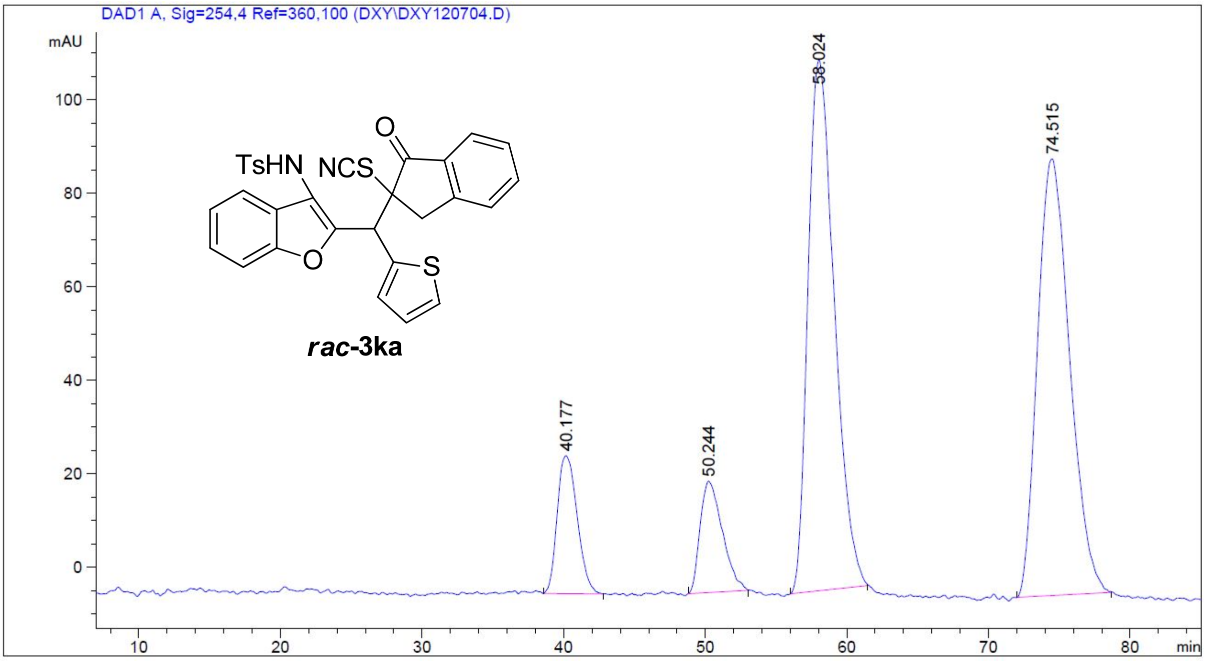

Peak #	RetTime [min]	Type	Width [min]	Area [mAU*s]	Height [mAU]	Area %
1	40.177	BB	1.1504	2896.88110	29.48141	8.3079
2	50.244	BB	1.2843	2600.54224	23.84565	7.4581
3	58.024	BB	1.5793	1.46532e4	113.57018	42.0239
4	74.515	BB	1.8827	1.47182e4	93.38760	42.2101

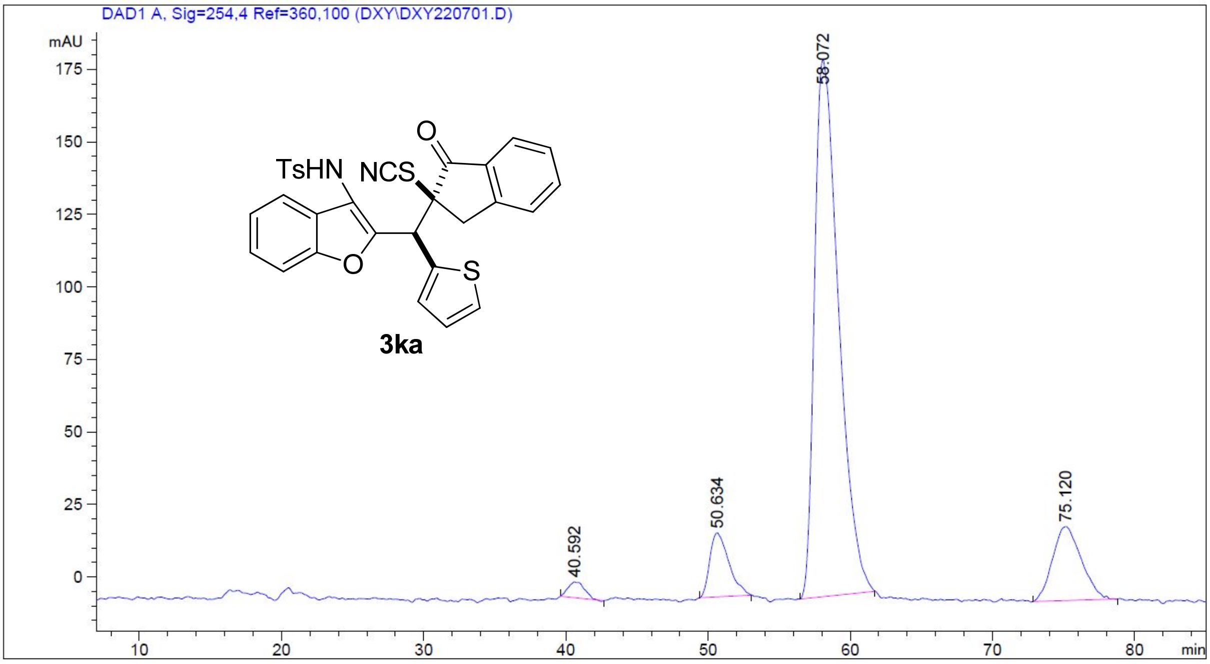

Peak #	RetTime [min]	Type	Width [min]	Area [mAU*s]	Height [mAU]	Area %
1	40.592	BB	0.9489	433.20798	5.42901	1.5509
2	50.634	BB	1.0990	2051.69653	22.06074	7.3453
3	58.072	BB	1.6054	2.18462e4	185.03654	78.2120
4	75.120	BB	1.6570	3600.90771	25.53785	12.8917

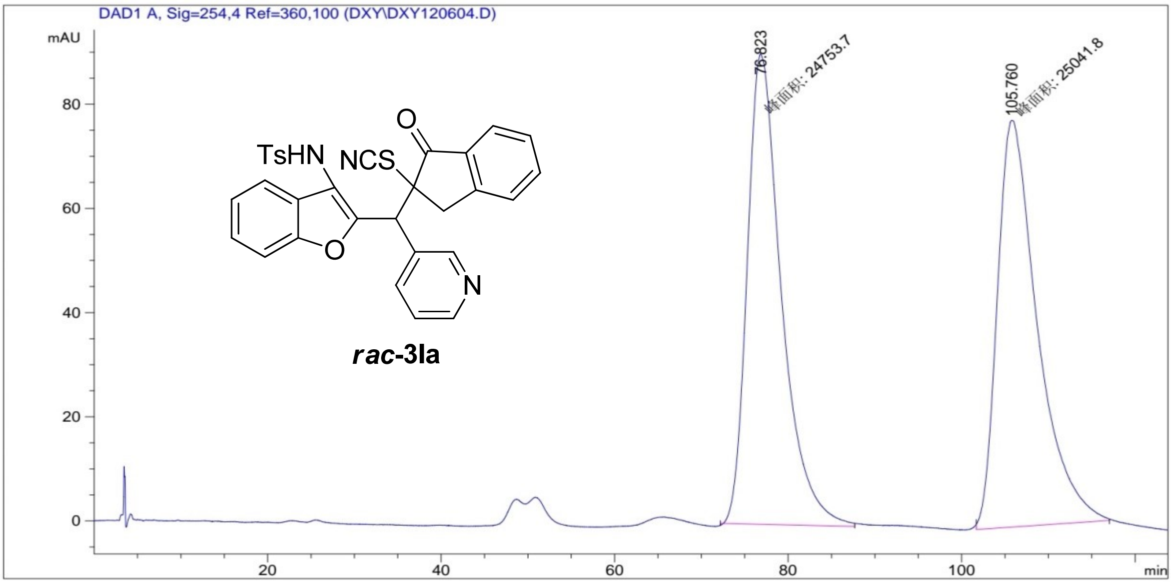

Peak #	RetTime [min]	Type	Width [min]	Area [mAU*s]	Height [mAU]	Area %
1	76.823	MM	4.5664	2.47537e4	90.34765	49.7107
2	105.760	MM	5.3463	2.50418e4	78.06610	50.2893

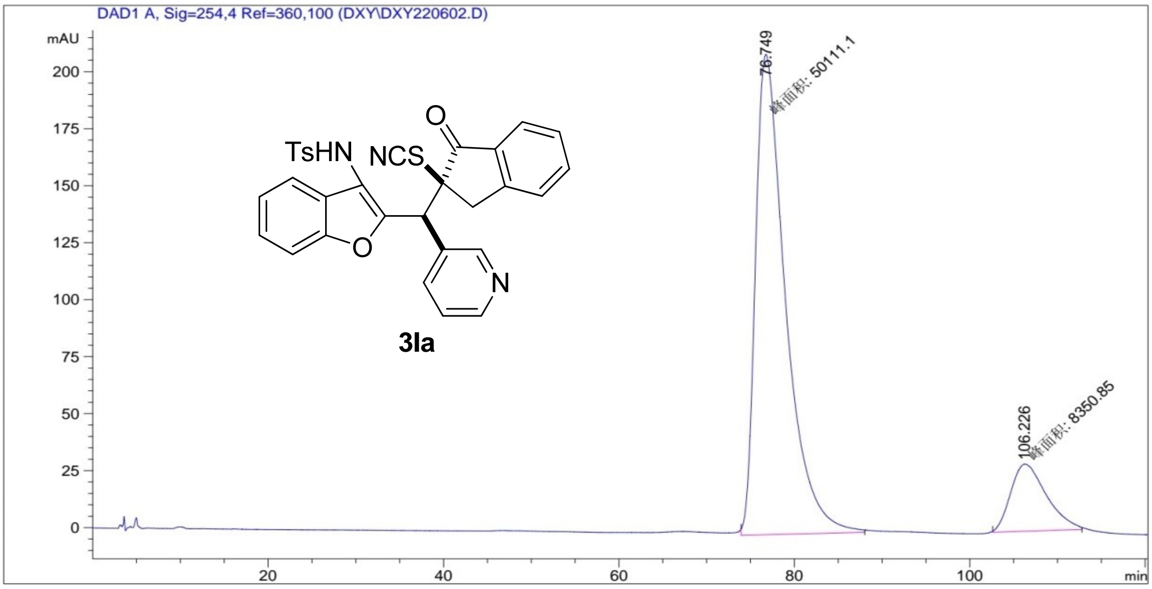

Peak #	RetTime [min]	Type	Width [min]	Area [mAU*s]	Height [mAU]	Area %
1	76.749	MM	3.9631	5.01111e4	210.74255	85.7158
2	106.226	MM	4.7173	8350.84668	29.50456	14.2842

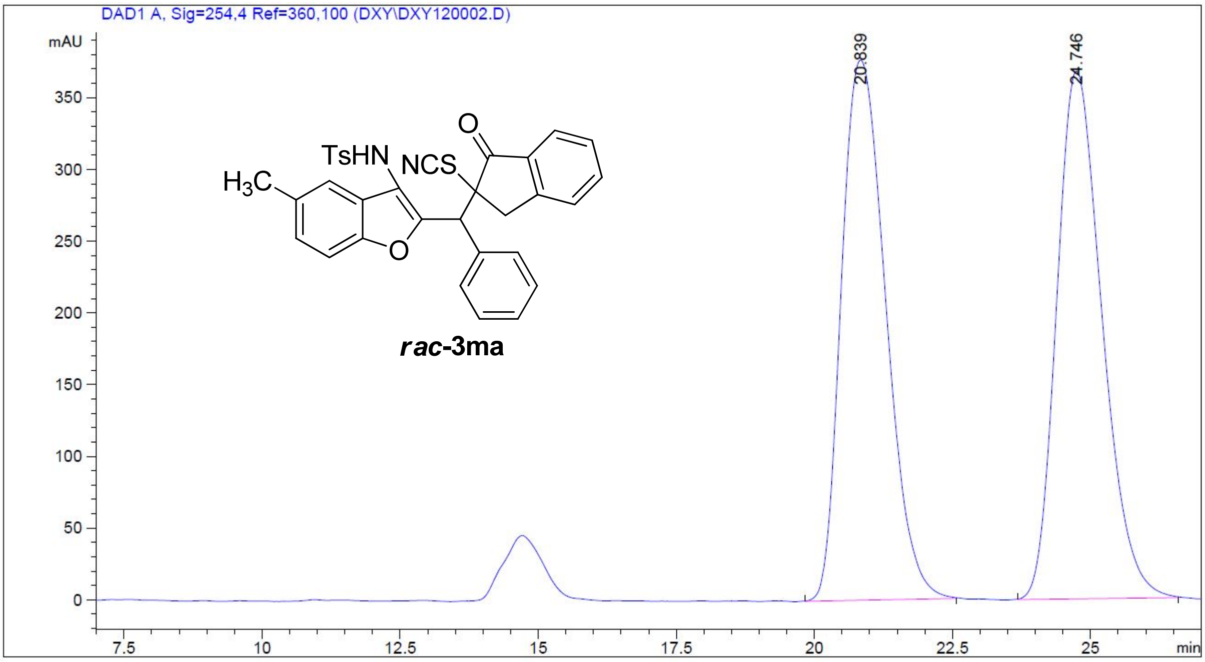

Peak #	RetTime [min]	Type	Width [min]	Area [mAU*s]	Height [mAU]	Area %
1	20.839	BB	0.8779	2.09753e4	376.52472	49.9655
2	24.746	BB	0.8705	2.10042e4	367.66370	50.0345

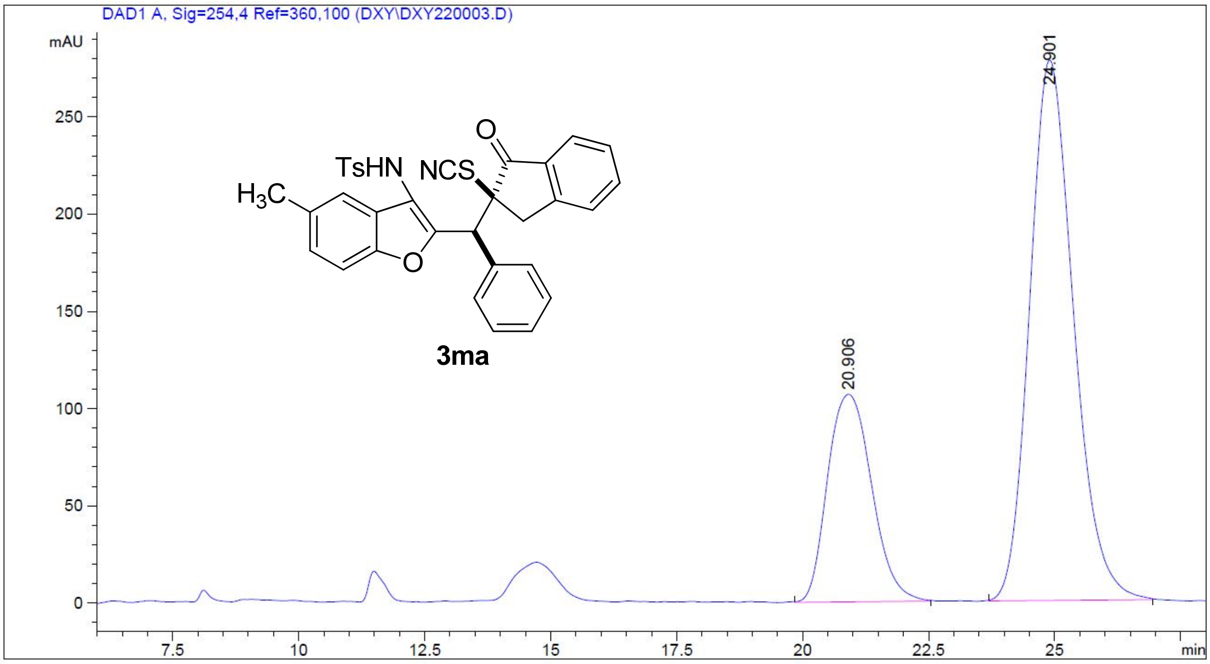

Peak #	RetTime [min]	Type	Width [min]	Area [mAU*s]	Height [mAU]	Area %
1	20.906	BB	0.9554	6505.36865	106.68855	27.7170
2	24.901	MM	1.0149	1.69653e4	278.59573	72.2830

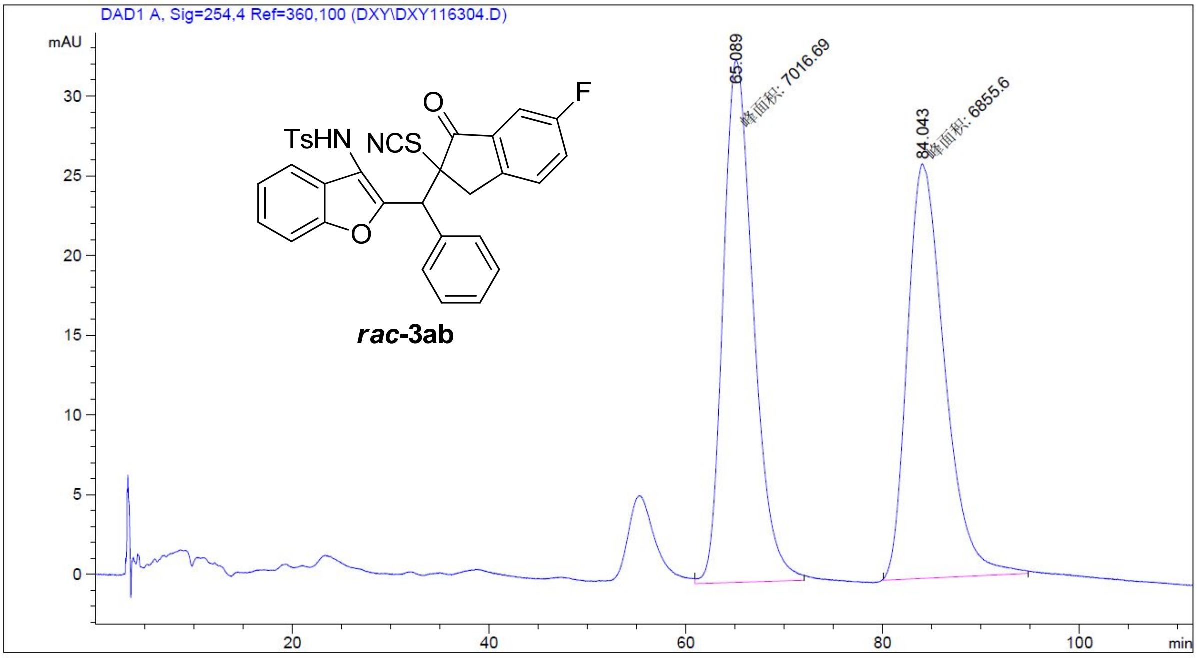

Peak #	RetTime [min]	Type	Width [min]	Area [mAU*s]	Height [mAU]	Area %
1	65.089	MM	3.5684	7016.69287	32.77230	50.5806
2	84.043	MM	4.3912	6855.59717	26.02042	49.4194

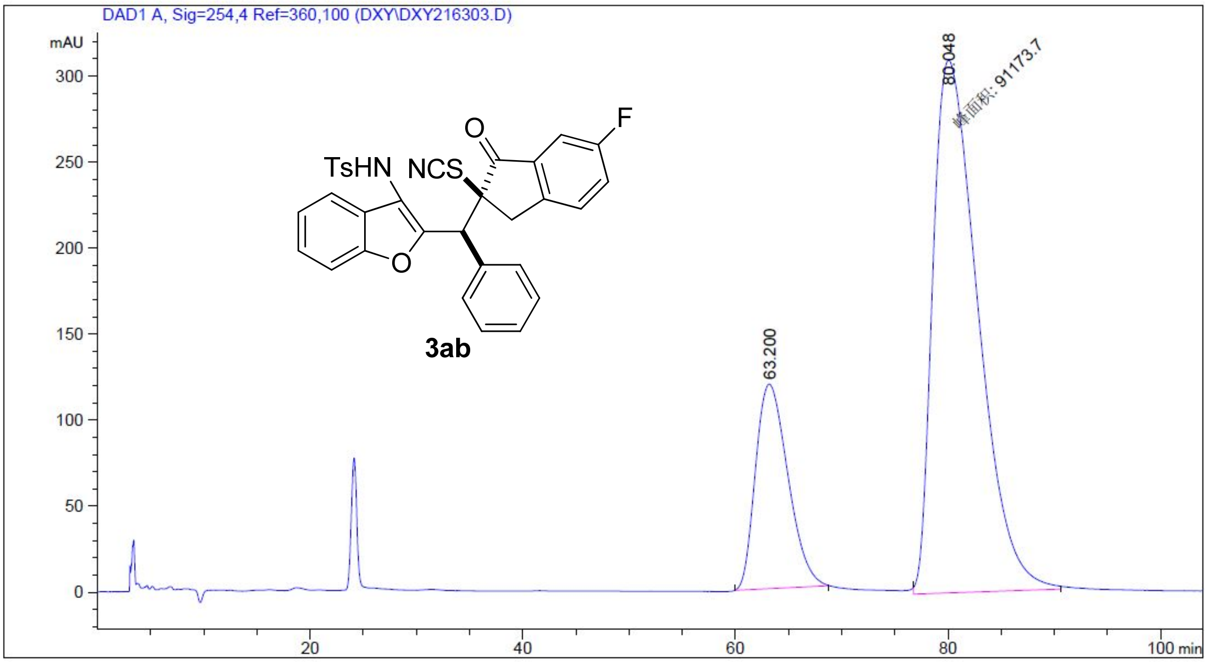

Peak #	RetTime [min]	Type	Width [min]	Area [mAU*s]	Height [mAU]	Area %
1	63.200	BB	2.4956	2.51861e4	118.88543	21.6450
2	80.048	MM	4.9098	9.11737e4	309.49326	78.3550

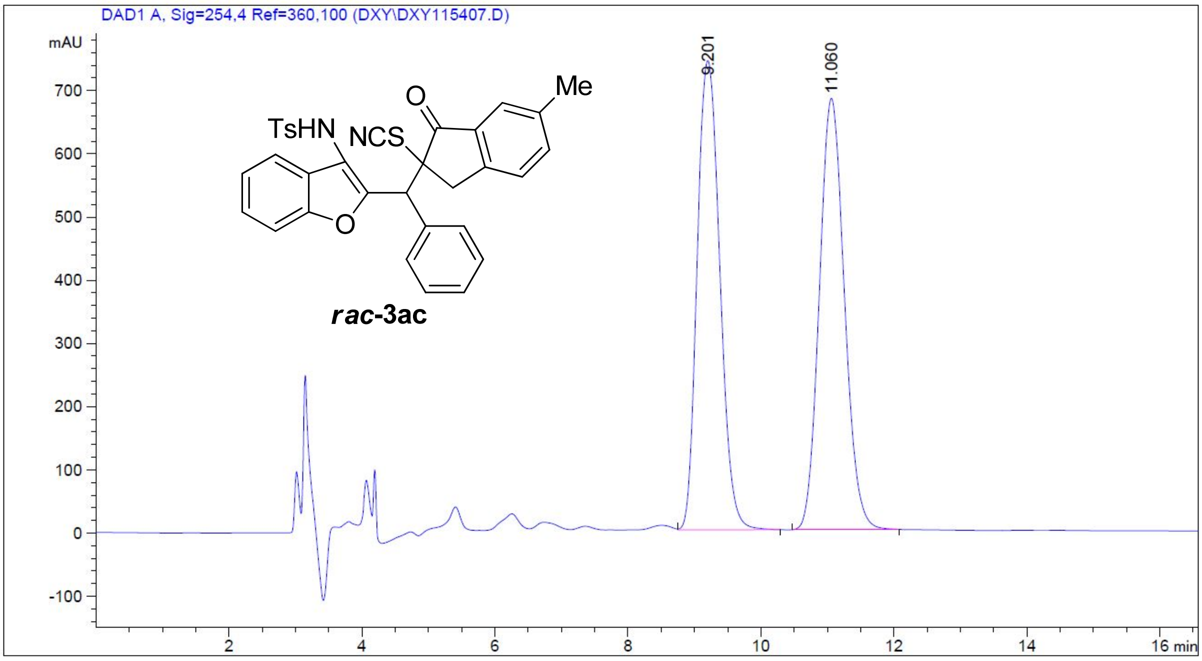

Peak #	RetTime [min]	Type	Width [min]	Area [mAU*s]	Height [mAU]	Area %
1	9.201	VB	0.3784	1.75882e4	742.50629	49.6146
2	11.060	BB	0.4072	1.78614e4	682.72522	50.3854

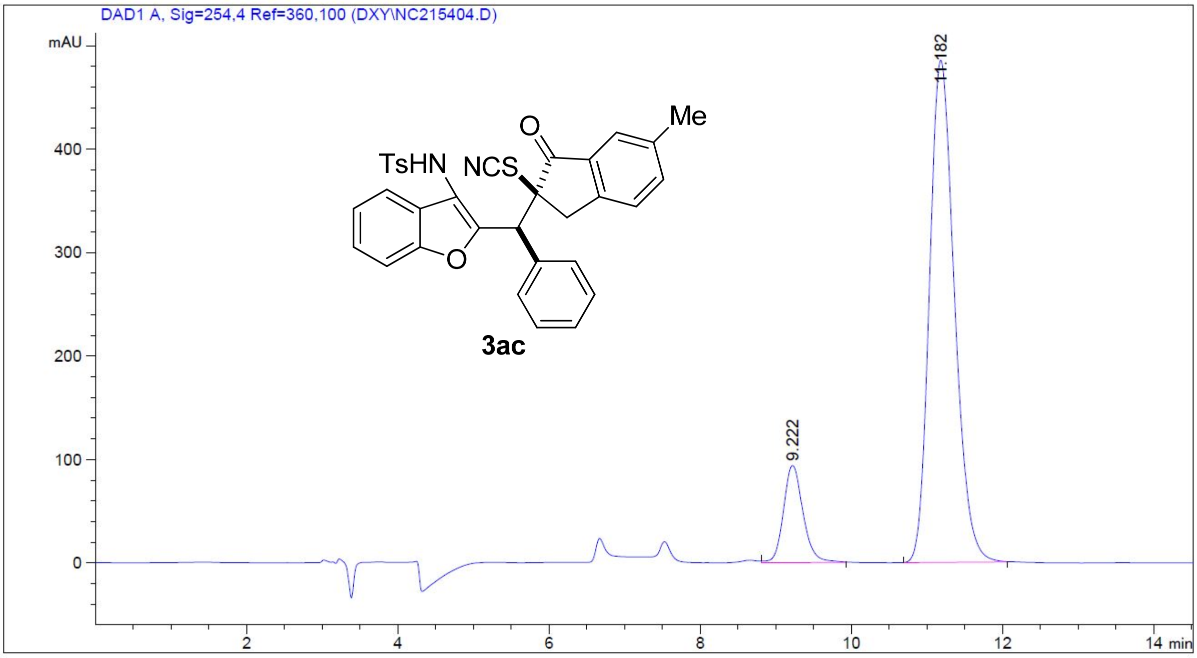

Peak #	RetTime [min]	Type	Width [min]	Area [mAU*s]	Height [mAU]	Area %
1	9.222	VB	0.2740	1690.63110	93.90810	13.1773
2	11.182	BB	0.3537	1.11392e4	485.49158	86.8227

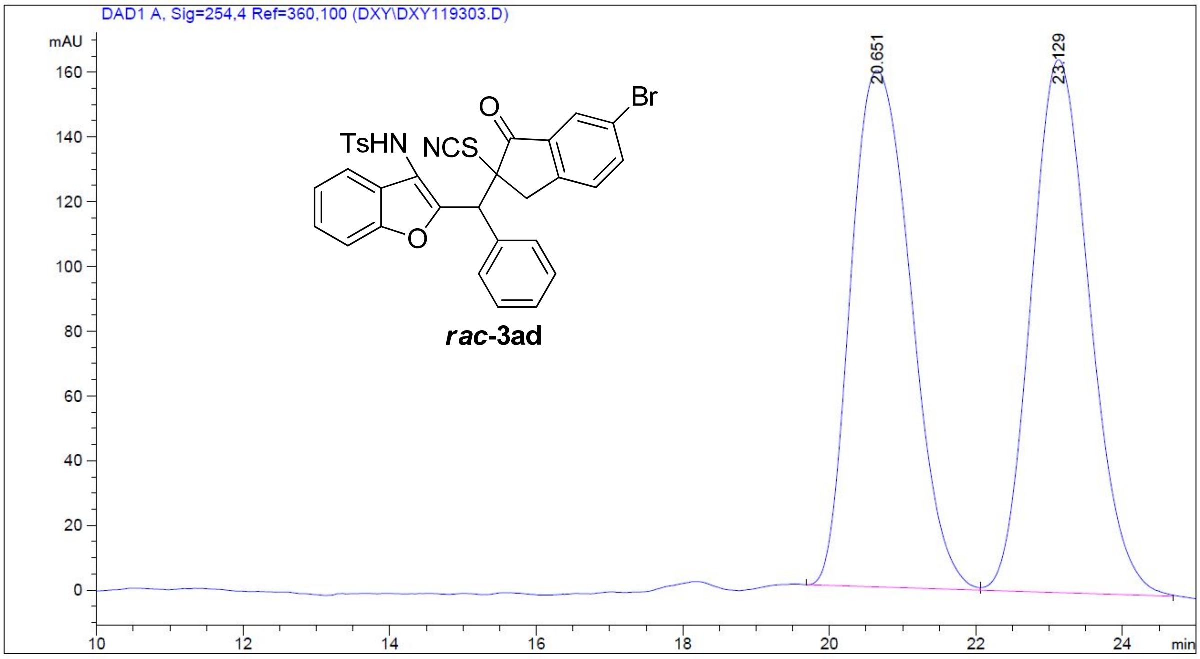

Peak #	RetTime [min]	Type	Width [min]	Area [mAU*s]	Height [mAU]	Area %
1	20.651	BV	0.9055	9072.13770	159.51590	49.7634
2	23.129	VB	0.8390	9158.41309	164.63664	50.2366

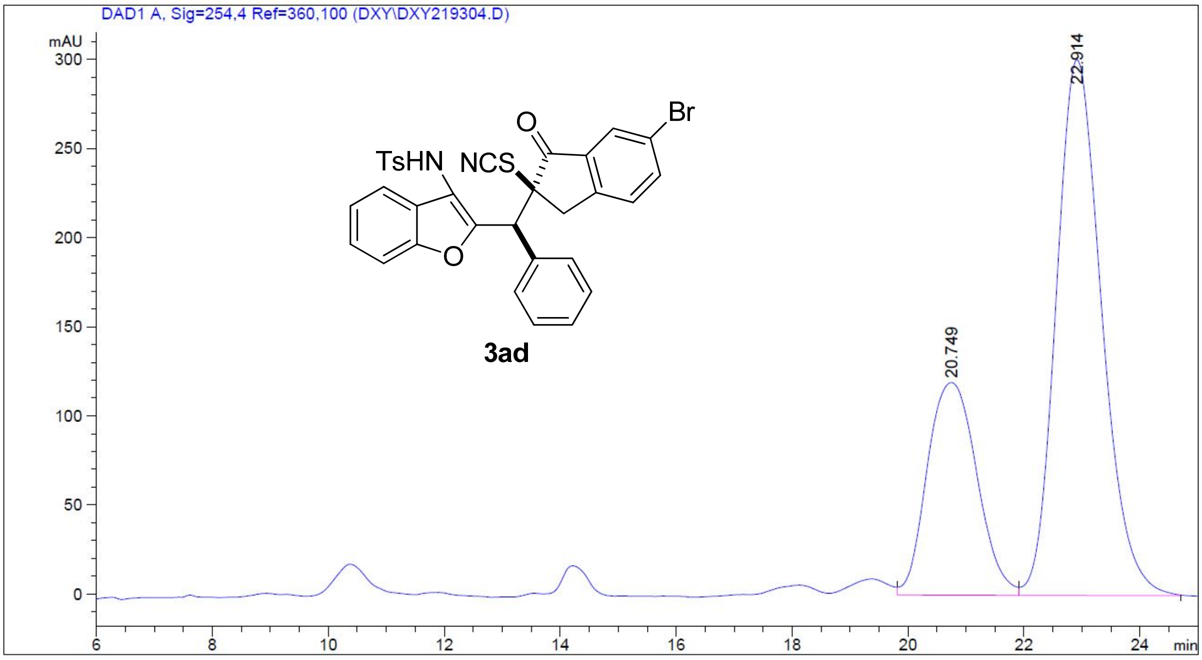

Peak #	RetTime [min]	Type	Width [min]	Area [mAU*s]	Height [mAU]	Area %
1	20.749	VV	0.9411	7103.33203	119.21996	30.2041
2	22.914	VB	0.8473	1.64144e4	300.51007	69.7959

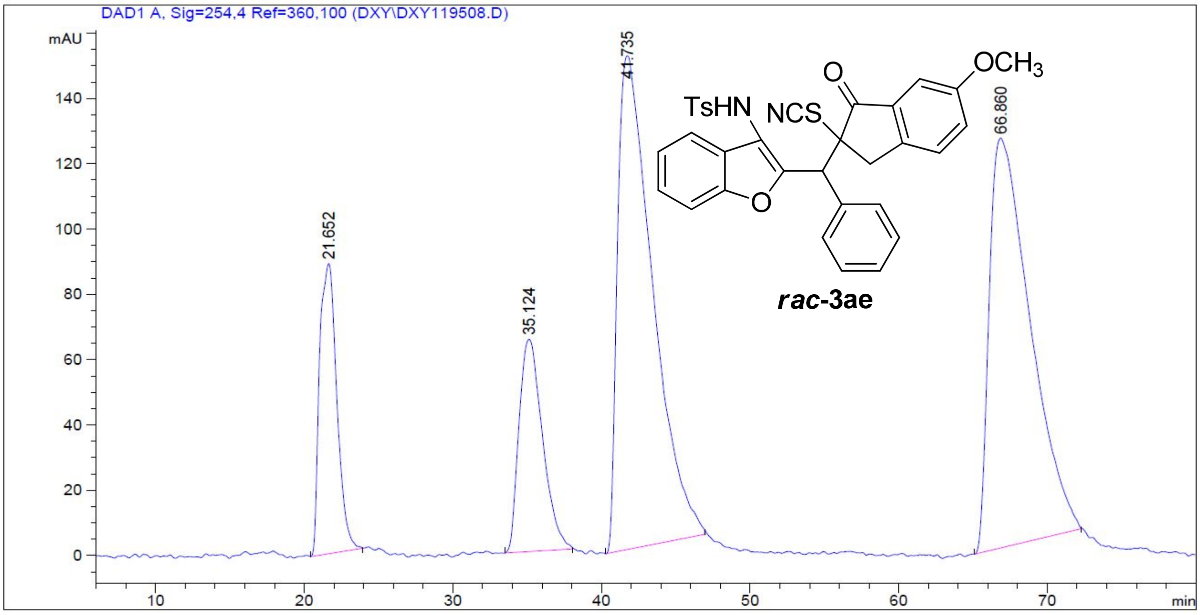

Peak #	RetTime [min]	Type	Width [min]	Area [mAU*s]	Height [mAU]	Area %
1	21.652	BB	1.0701	7157.16113	88.82510	11.3775
2	35.124	BB	1.2992	7160.34570	65.00050	11.3825
3	41.735	BB	1.9184	2.46821e4	151.20845	39.2362
4	66.860	BB	2.2592	2.39068e4	125.56573	38.0037

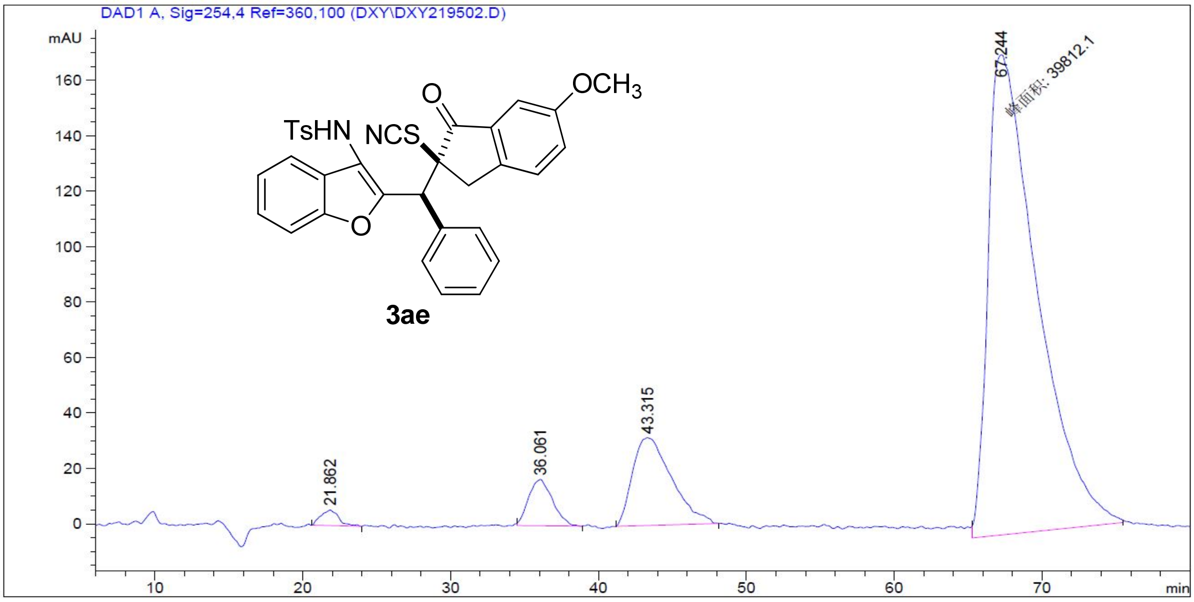

Peak #	RetTime [min]	Type	Width [min]	Area [mAU*s]	Height [mAU]	Area %
1	21.862	BB	0.9858	471.77490	5.66369	0.9831
2	36.061	BB	1.3291	1880.58130	16.68325	3.9189
3	43.315	BB	2.1578	5823.05762	31.68889	12.1345
4	67.244	MM	3.8298	3.98121e4	173.25388	82.9635

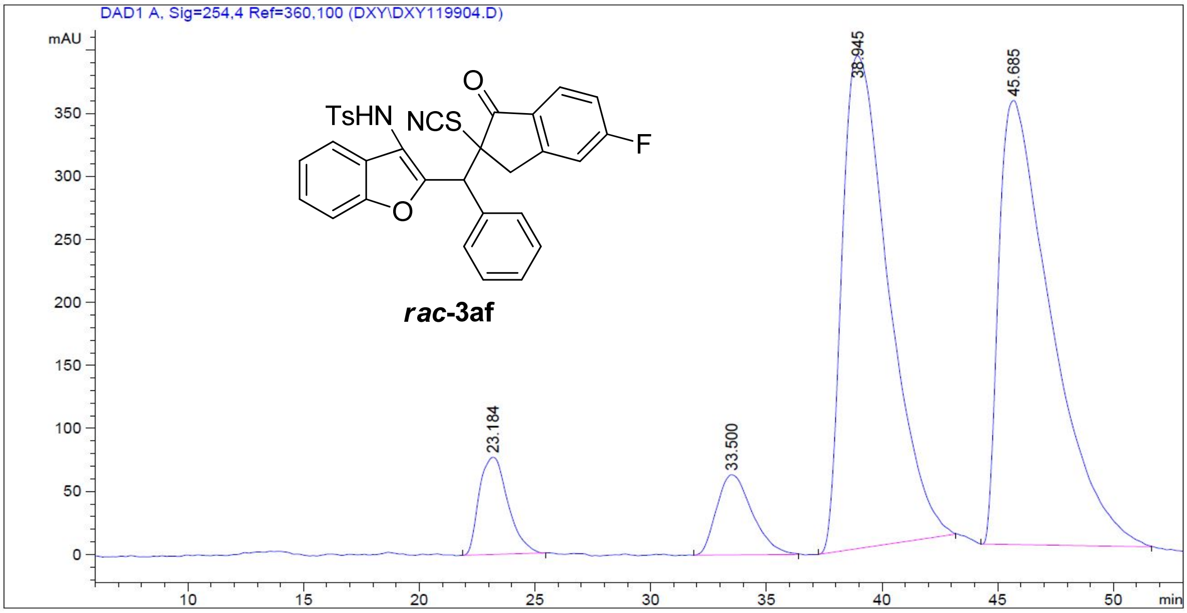

Peak #	RetTime [min]	Type	Width [min]	Area [mAU*s]	Height [mAU]	Area %
1	23.184	BB	1.1626	6634.78711	77.05020	5.3713
2	33.500	BB	1.2835	6786.89893	63.57086	5.4944
3	38.945	BB	1.6701	5.45059e4	391.05997	44.1258
4	45.685	BB	1.9070	5.55964e4	352.31879	45.0086

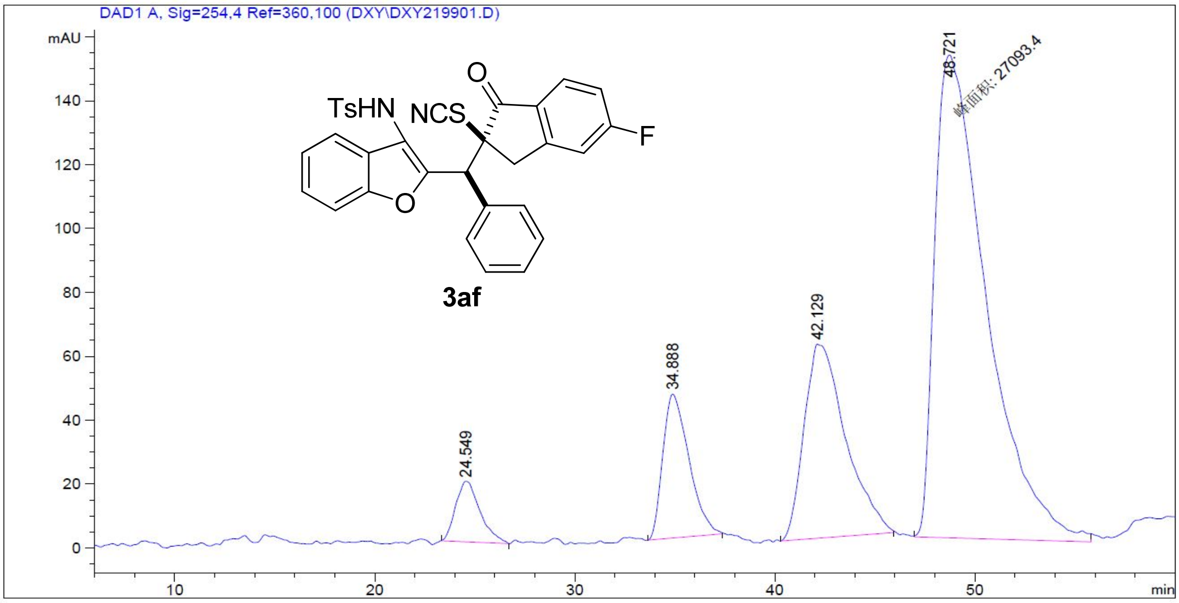

Peak #	RetTime [min]	Type	Width [min]	Area [mAU*s]	Height [mAU]	Area %
1	24.549	BB	1.0003	1604.62793	18.94220	3.8619
2	34.888	BB	1.0957	4092.61035	45.05836	9.8497
3	42.129	BB	1.6906	8759.80957	60.73634	21.0823
4	48.721	MM	2.9873	2.70934e4	151.16130	65.2060

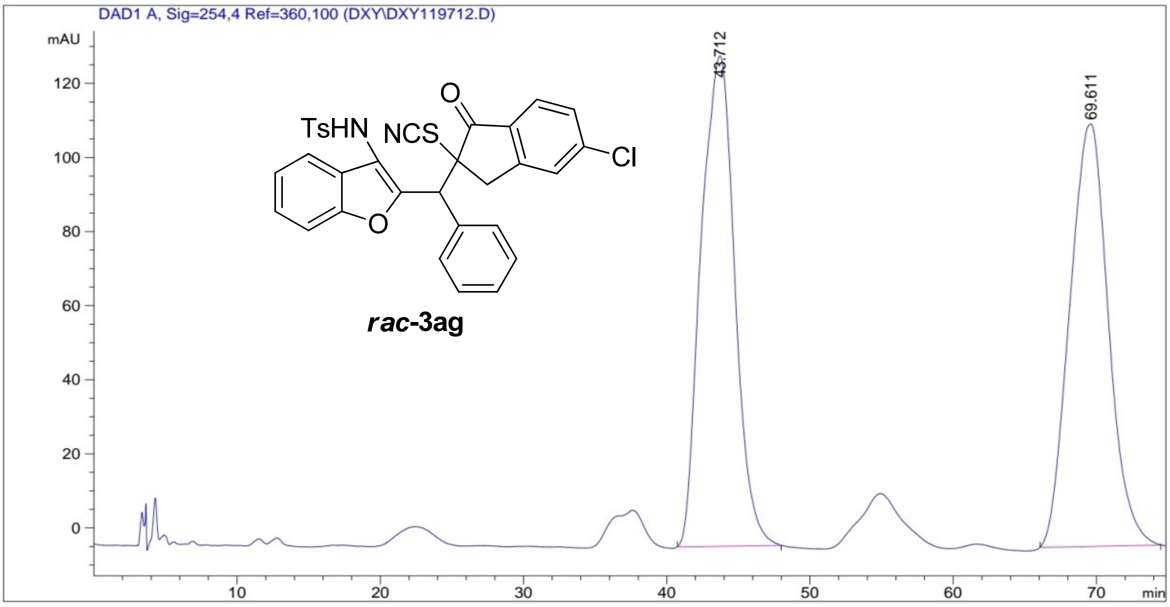

Peak #	RetTime [min]	Type	Width [min]	Area [mAU*s]	Height [mAU]	Area %
1	43.712	BB	1.9388	2.16176e4	132.40707	50.2710
2	69.611	BB	2.2139	2.13845e4	114.03066	49.7290

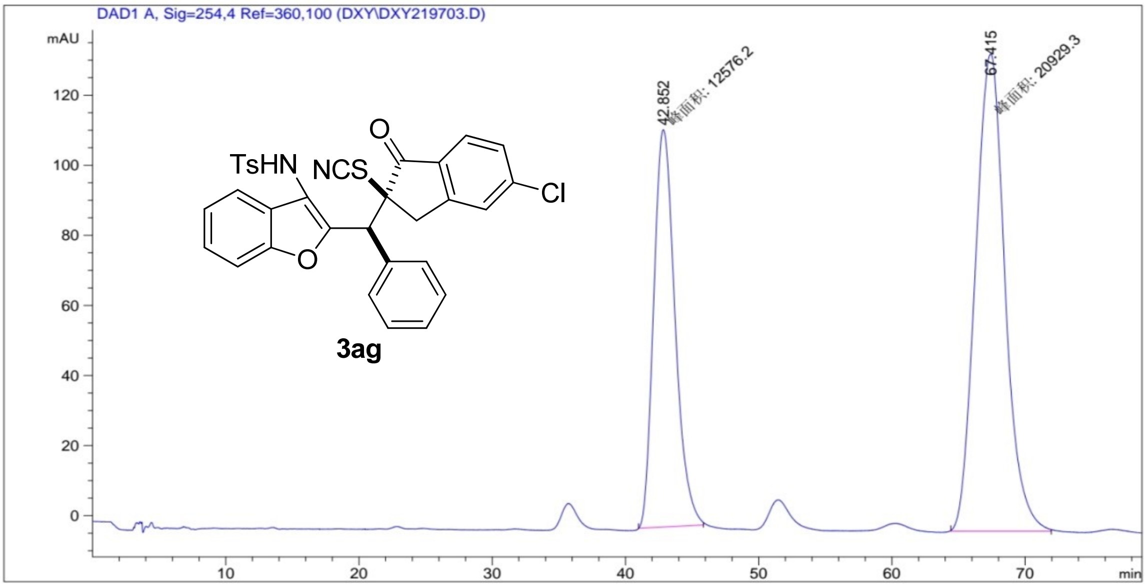

Peak #	RetTime [min]	Type	Width [min]	Area [mAU*s]	Height [mAU]	Area %
1	42.852	MM	1.8487	1.25762e4	113.37922	37.5348
2	67.415	MM	2.5612	2.09293e4	136.19647	62.4652

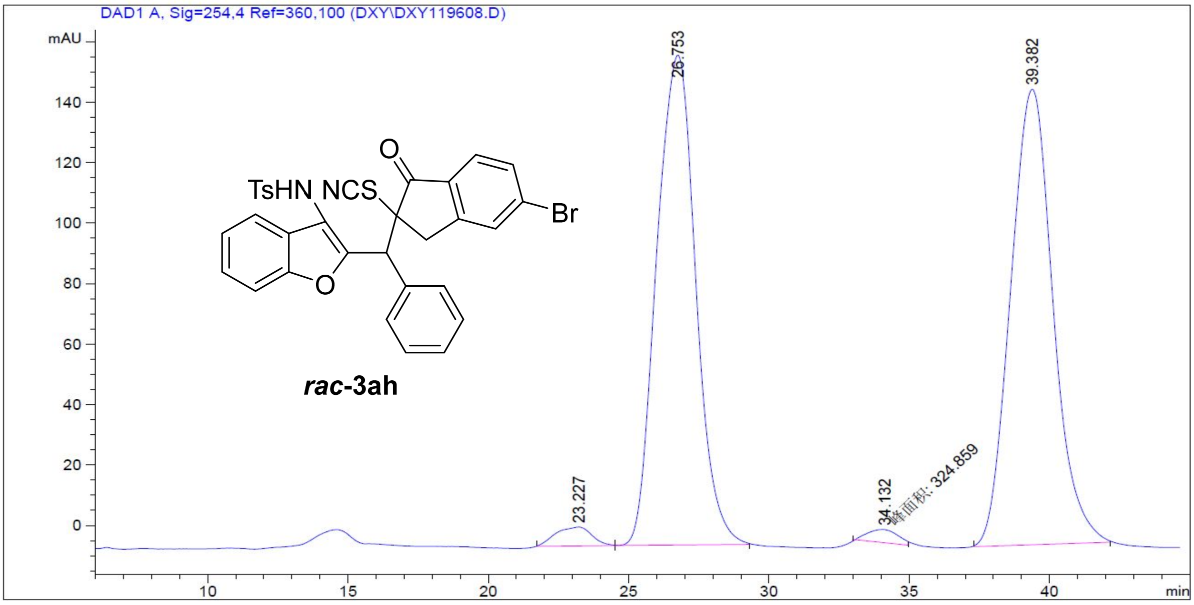

Peak #	RetTime [min]	Type	Width [min]	Area [mAU*s]	Height [mAU]	Area %
1	23.227	BV	1.0533	558.03552	6.25201	1.7222
2	26.753	VB	1.3646	1.56773e4	162.06383	48.3829
3	34.132	MM	1.2389	324.85886	4.37042	1.0026
4	39.382	BB	1.5207	1.58424e4	150.66936	48.8923

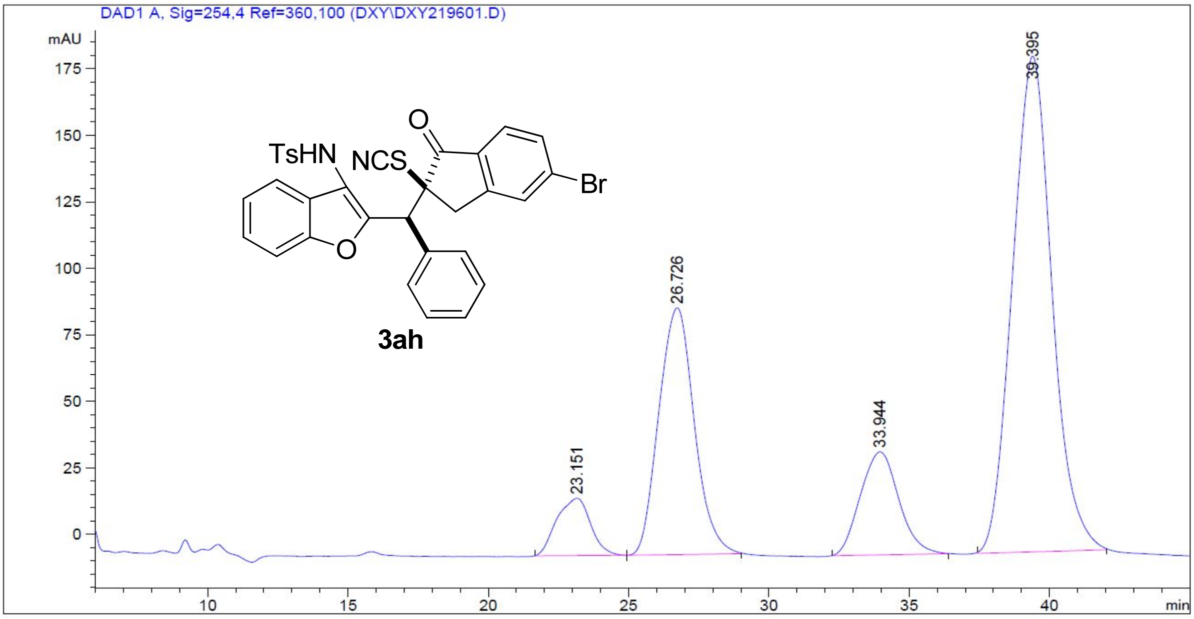

Peak #	RetTime [min]	Type	Width [min]	Area [mAU*s]	Height [mAU]	Area %
1	23.151	BV	1.0803	1800.66040	21.51786	5.6462
2	26.726	VB	1.2548	8147.34326	92.75043	25.5472
3	33.944	BB	1.1703	3690.00732	38.70226	11.5706
4	39.395	BB	1.4633	1.82533e4	186.26125	57.2360

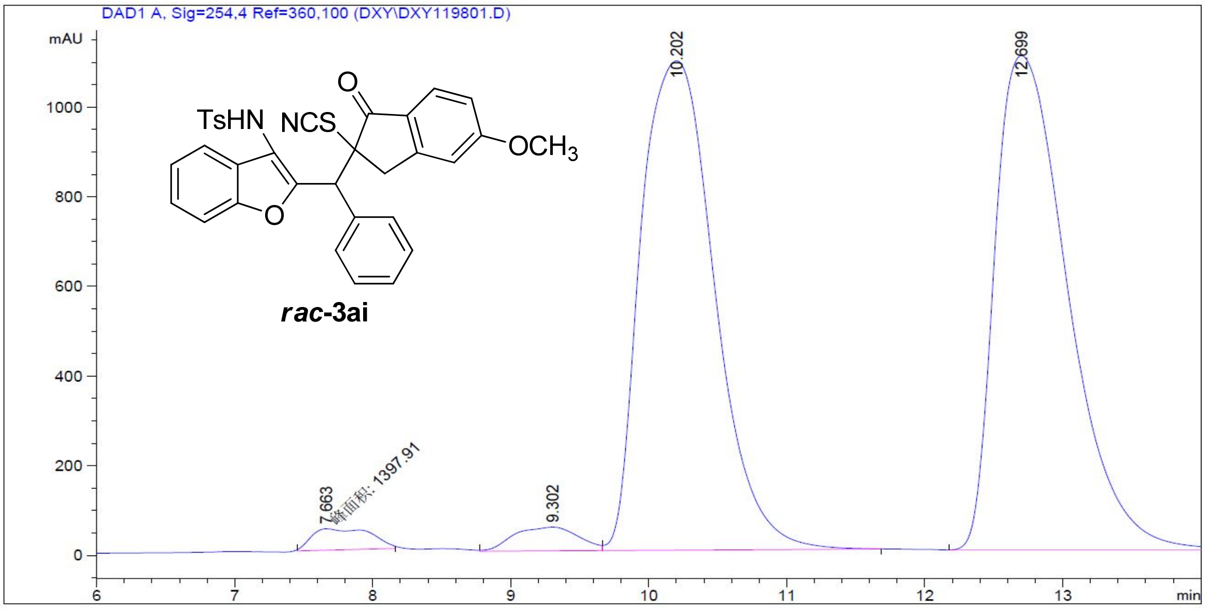

Peak #	RetTime [min]	Type	Width [min]	Area [mAU*s]	Height [mAU]	Area %
1	7.663	MM	0.4888	1397.91064	47.66300	1.6636
2	9.302	VV	0.4470	1719.60266	52.93014	2.0465
3	10.202	VB	0.5948	4.05016e4	1091.87708	48.2003
4	12.699	BBA	0.5676	4.04085e4	1102.39392	48.0896

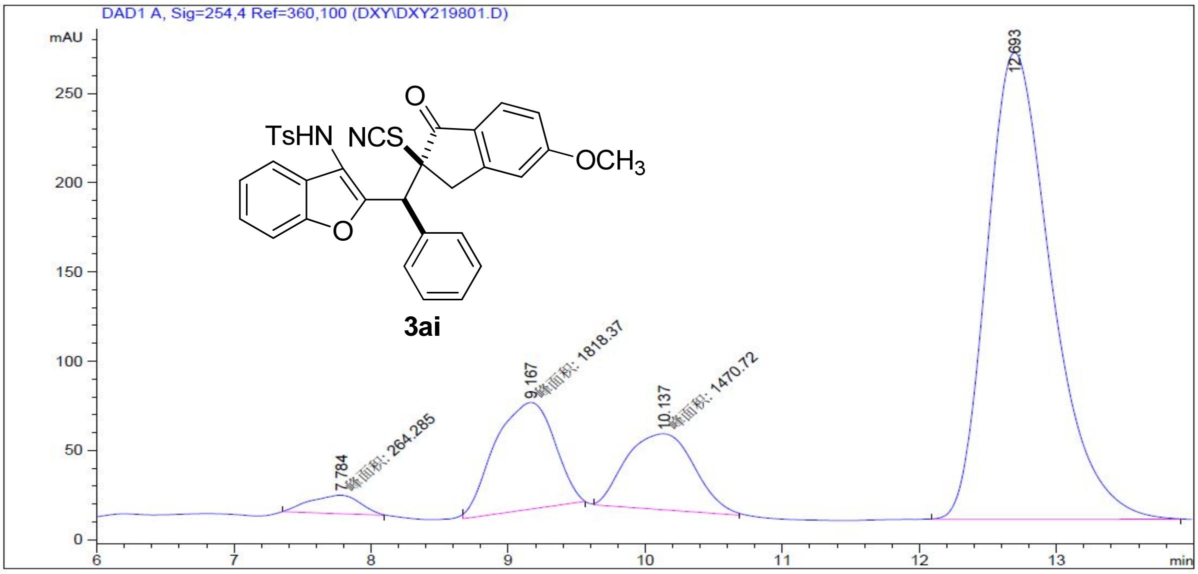

Peak #	RetTime [min]	Type	Width [min]	Area [mAU*s]	Height [mAU]	Area %
1	7.784	MM	0.4233	264.28476	10.40521	2.1826
2	9.167	MM	0.5067	1818.37415	59.80950	15.0172
3	10.137	MM	0.5746	1470.71570	42.66147	12.1460
4	12.693	BB	0.5038	8555.24219	261.43372	70.6542
